# Anomalous spin–lattice coupling in a 2D antiferromagnetic semiconductor revealed by surface acoustic Rayleigh waves

**DOI:** 10.1038/s41467-026-77234-4

**Published:** 2026-10-12

**Authors:** Zahra Ebrahim Nataj, Md Sabbir Hossen Bijoy, Vladislav Korostelev, Jordan Teeter, Dylan Wright, Mohammad Zeinolabedini, Konstantin Klyukin, Fariborz Kargar, Alexander A. Balandin

**Affiliations:** 1 Department of Materials Science and Engineering, University of California, Los Angeles, CA USA; 2 California NanoSystems Institute, University of California, Los Angeles, CA USA; 3 Department of Mechanical Engineering, Materials Research and Education Center, Auburn University, Auburn, AL USA

**Keywords:** Magnetic properties and materials, Two-dimensional materials

## Abstract

Magnetic order in van der Waals magnets can strongly influence their lattice dynamics, yet how it affects local and macroscopic manifestations of spin-phonon coupling remains unclear. Optical phonons probe bond-scale exchange modulation and short-range spin correlations, whereas long-wavelength acoustic modes couple to uniform strain fields and are sensitive to renormalization of the macroscopic elastic tensor associated with long-range magnetic order. Experimentally accessing these acoustic excitations in low-dimensional crystals is challenging due to their low energies and the small lateral dimensions of exfoliated samples. Here, we employ Brillouin–Mandelstam scattering spectroscopy to investigate surface acoustic phonons in exfoliated NiPS_3_ thin films across the antiferromagnetic transition. Our results show a single Rayleigh surface mode whose phase velocity decreases significantly across the Néel temperature. This anomaly reflects a strong magnetoelastic renormalization of the elastic constants triggered by the onset of antiferromagnetic order. First-principles calculations, combined with continuum finite-element modeling reproduce the Rayleigh-wave dispersion and its magnetic-order–induced shift. The results reveal how microscopic exchange interactions govern the macroscopic elastic response of two-dimensional antiferromagnetic semiconductors, enabling magnetically tunable phononic, magnonic, and strain-mediated spintronic devices.

## Abstract (web summary)

While there is now an extensive literature on spin-phonon coupling in van der Waals magnets, crucially, these have been limited to Raman-active optical modes that probe local spin correlations. Here, Nataj et al use angle resolved Brillouin–Mandelstam scattering spectroscopy to investigate the influence of long-range magnetic order on spin-phonon coupling.

## Introduction

Two-dimensional (2D) van der Waals (vdW) magnets offer a unique platform for probing and controlling the interplay between spin and lattice degrees of freedom at the atomic scale^1–3^. While the Mermin–Wagner theorem prohibits long-range magnetic order in the limit of a single isotropic layer^4^, real vdW magnets stabilize finite-temperature order through magneto-crystalline anisotropy arising from spin–orbit coupling, which lifts the degeneracy of spin orientations and opens a gap in the spin-wave spectrum^2,5–8^. The magnitude of this anisotropy is highly sensitive to lattice geometry since the exchange pathways and crystal-field environment depend on interatomic bond lengths and angles^8,9^. Spin–phonon, i.e., magnetoelastic, coupling provides the microscopic link between lattice dynamics and magnetic anisotropy, enabling atomic displacements and strain fields to modulate exchange interactions and crystal-field splitting^8–10^. Lattice distortions—whether static, e.g., strain, pressure, stacking, or dynamic, i.e., phonons—can renormalize the anisotropy and modify the magnetic excitation spectrum^8–12^. The reciprocal effect also holds when the onset of magnetic order reshapes the lattice through magnetostriction^13,14^. In the strict 2D limit, where the magnetic ground state is fragile, even small lattice distortions can strongly influence magnetic stability. Understanding phonon renormalization and elastic softening due to spin–phonon coupling provides insight into how exchange interactions and magnetic anisotropy evolve across the transition and how the lattice participates in stabilizing the ordered phase.

In many ferro- and antiferromagnets, the strength of spin–phonon coupling has traditionally been evaluated by tracking the temperature dependence of Raman-active optical phonons across the magnetic ordering transition^15–19^. In this approach, deviations of the phonon frequency below Néel temperature, ${T}_{N}$, from the expected anharmonic temperature trend are attributed to the influence of evolving spin correlations on local bonding environments^19^. Note that optical modes involve out-of-phase atomic motions within the unit cell and are therefore sensitive to short-range exchange pathways, making Raman spectroscopy an effective probe of local spin correlations^20^. Microscopically, this arises because the phonon frequency shift depends on the spin–spin correlation function $\langle {S}_{i}\cdot {S}_{j}\rangle$, which reflects nearest-neighbor exchange interactions and persists even above ${T}_{N}$ where only short-range magnetic correlations remain^20^. Optical phonons, therefore, primarily reflect bond-scale exchange modulation and do not directly capture the macroscopic elastic response of the lattice associated with long-range magnetic ordering. Acoustic phonons, in contrast, correspond to long-wavelength, in-phase lattice displacements governed by the macroscopic bulk and shear moduli. Their softening or hardening across the magnetic transition reflects the magnetoelastic renormalization of the elastic constants, providing a direct measure of coupling at collective, continuum length scales^21–24^. Despite their distinct importance at larger length scales, experimental studies have predominantly focused on optical phonons, and the behavior of acoustic phonons in vdW magnets remains largely unexplored due to the challenges of probing them in small-size exfoliated samples with conventional neutron or ultrasound methods.

In this work, we investigate how antiferromagnetic (AFM) ordering in NiPS_3_ influences its long-wavelength elastic response by directly probing surface acoustic phonons in exfoliated thin films on SiO_2_/Si substrates. Although the influence of magnetic ordering on local bond-scale dynamics in NiPS_3_ is well established, it remains unclear whether and how this ordering modifies its long-wavelength elastic response. NiPS_3_ is a quasi-two-dimensional antiferromagnet with XY-type spin anisotropy and dominant intralayer exchange interactions^9,25–27^. The Ni²⁺ spins order into a zigzag pattern below its Néel temperature, ${T}_{N} \sim 155\,{{\rm{K}}}$, producing two low-energy magnon gaps and a spin–orbit–entangled excitonic excitation observed in neutron and Raman experiments^28,29^. Prior Raman studies have revealed mode-selective spin–phonon coupling in specific optical modes of NiPS_3_, including pronounced Fano-like line-shape modifications and mode splitting below its Néel temperature^16,30^. However, the response of low-energy acoustic phonons to AFM ordering in NiPS_3_ remains unknown. We employ angle-resolved Brillouin–Mandelstam light scattering (BMS) spectroscopy, which allows access to Rayleigh-type surface acoustic modes confined in micron-scale supported films. Tracking these modes across the Néel temperature enables a direct measurement of how the elastic constants evolve with spin ordering. By combining the BMS measurements with first-principles elastic tensor calculations and continuum finite-element modeling, we establish a quantitative link between magnetic order and lattice elasticity in NiPS_3_. This approach provides a pathway for assessing magnetoelastic coupling in vdW AFM semiconductors and informs the design of phononic and spintronic devices that leverage spin–lattice interactions.

## Results and discussions

**Fig. 1:**
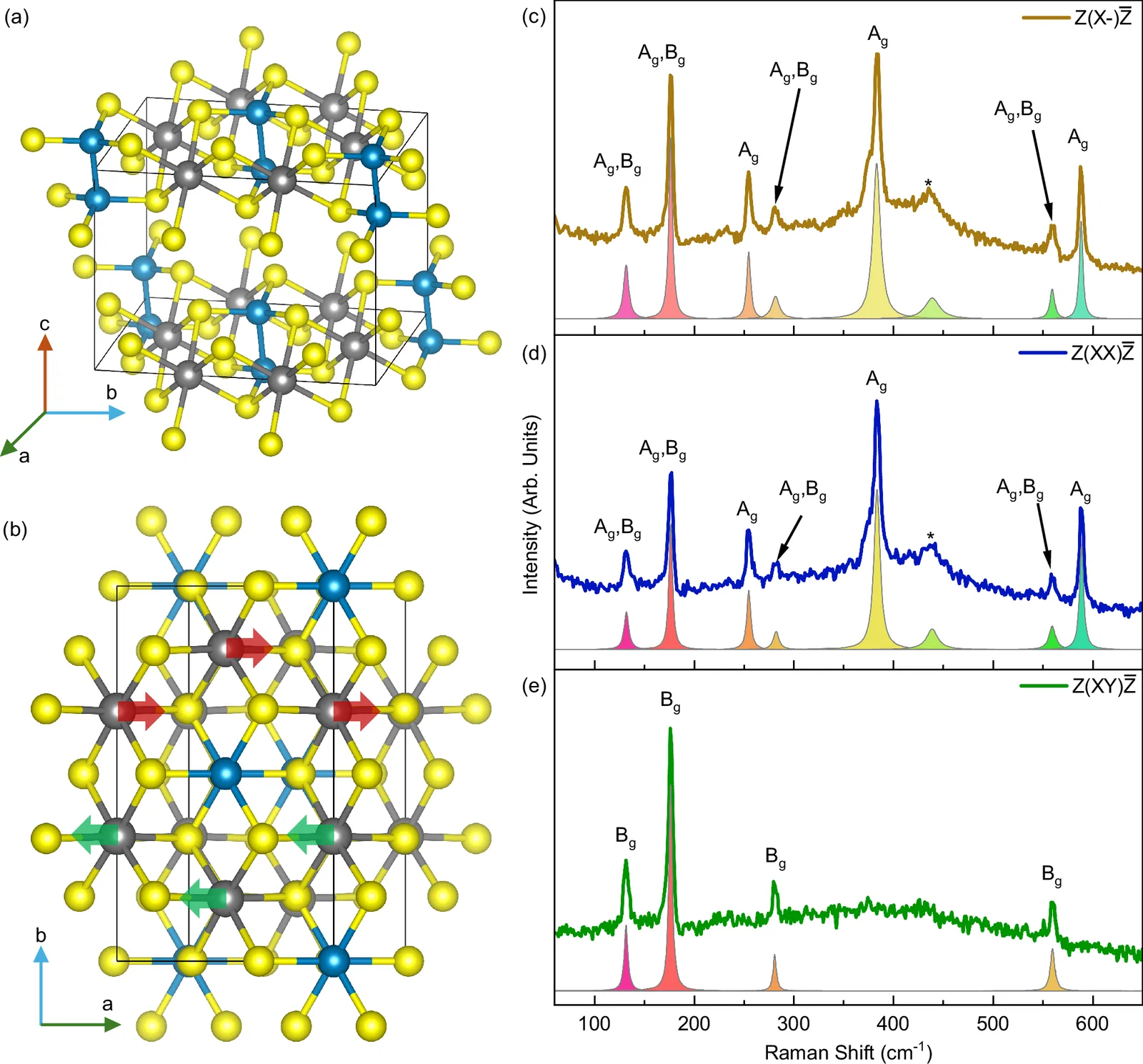
Crystal, magnetic, and vibrational structure of monoclinic NiPS_3_. **a** Crystal structure of monoclinic NiPS_3_. Ni atoms form a honeycomb lattice coordinated by P_2_S_6_ units. Ni, P, and S atoms are depicted with black, blue, and yellow spheres, respectively. **b** View of the structure along the *c*-axis. The arrows show the magnetic spin orientation lying predominantly within the *ab* plane. **c**–**e** Raman spectra collected in unpolarized, $z\left(x-\right)\bar{z}$, parallel $z\left({xx}\right)\bar{z}$, and cross-polarized $z\left({xy}\right)\bar{z}$ configurations, respectively. The polarization-selective measurements enable the identification of ${A}_{{{\rm{g}}}}$ and ${B}_{{{\rm{g}}}}$ phonon symmetries. Peaks are fitted using Lorentzian functions shown as shaded areas.

In this study, we used high-quality single crystals of NiPS_3_ (2D Semiconductors, USA). Thin films were prepared by mechanical exfoliation onto SiO_2_/Si substrates with a 300 nm oxide layer. Atomic force microscopy (AFM) measurements were performed to determine the thickness of the exfoliated samples, and two thin films with thicknesses of h~220 nm and ~260 nm were selected for this study (Supplementary Figs. 1 and 2). Figure 1a shows the bulk crystal structure of NiPS_3_. The compound crystallizes in the monoclinic space group C2/m. Within each layer, Ni atoms form a honeycomb structure coordinated by P_2_S_6_ molecular units^31^. Figure 1b illustrates the magnetic ground state. Below the Néel temperature, NiPS_3_ adopts a zigzag AFM order in which ferromagnetic chains run along the *a*-axis and are antiferromagnetically coupled along the *b*-axis with the magnetic moments lying predominantly within the *ab* plane and having only a small out-of-plane component. Adjacent layers are additionally coupled antiferromagnetically along the *c*-axis. This magnetic ground state is characterized by the spin propagation vector **k** = [010]^32^.

Raman spectroscopy was performed on the exfoliated NiPS_3_ films using a 532 nm laser excitation at room temperature (RT) to confirm the sample quality. Bulk NiPS_3_ belongs to *C*_2h_ point group and its irreducible Γ-point phonon representation is $\varGamma=8{A}_{{{\rm{g}}}}+7{B}_{{{\rm{g}}}}+6{A}_{{{\rm{u}}}}+9{B}_{{{\rm{u}}}}$, where ${A}_{{{\rm{g}}}}$ and ${B}_{{{\rm{g}}}}$ modes are Raman active and ${A}_{{{\rm{u}}}}$ and ${B}_{{{\rm{u}}}}$ modes are infrared active^25,33,34^. Figure 1c shows the unpolarized Raman spectrum in which all Raman-active modes are observed, confirming the structural quality of the sample. The low-frequency modes arise mostly from the vibration of the Ni atoms, whereas the higher-frequency modes above originate from the intermolecular vibrations of the ${\left({{{\rm{P}}}}_{2}{{{\rm{S}}}}_{6}\right)}^{-4}$ units. As seen in Fig. 1c, several nearly-degenerate pairs of ${A}_{{{\rm{g}}}}$ and ${B}_{{{\rm{g}}}}$ modes are observed. These modes originate from the splitting of the monolayer ${E}_{{{\rm{g}}}}$ phonons due to the monoclinic stacking in bulk NiPS_3_, which lifts the degeneracy present in the monolayer^25,34^. To distinguish ${A}_{{{\rm{g}}}}$ and ${B}_{{{\rm{g}}}}$ symmetries, we conducted polarization-resolved Raman spectroscopy. In backscattering geometry, both ${A}_{{{\rm{g}}}}$ and ${B}_{{{\rm{g}}}}$ modes are visible in the parallel configuration, $z\left({xx}\right)\bar{z}$, while only ${B}_{{{\rm{g}}}}$ modes are present in the cross-polarized configuration, $z\left({xy}\right)\bar{z}$, as shown in Fig. 1d, e. Lorentzian fitting was applied to all Raman peaks, with the fitted components shown as shaded areas in Fig. 1c–e. The weak feature marked by an asterisk at ∼450 cm⁻¹ may originate from higher-order Raman scattering and is therefore not assigned here. The positions and the full-width-at-half-maximums (FWHM) of the observed peaks are listed in Supplementary Table 1. The obtained Raman peaks are consistent with previously published data^25,33,35^.

**Fig. 2:**
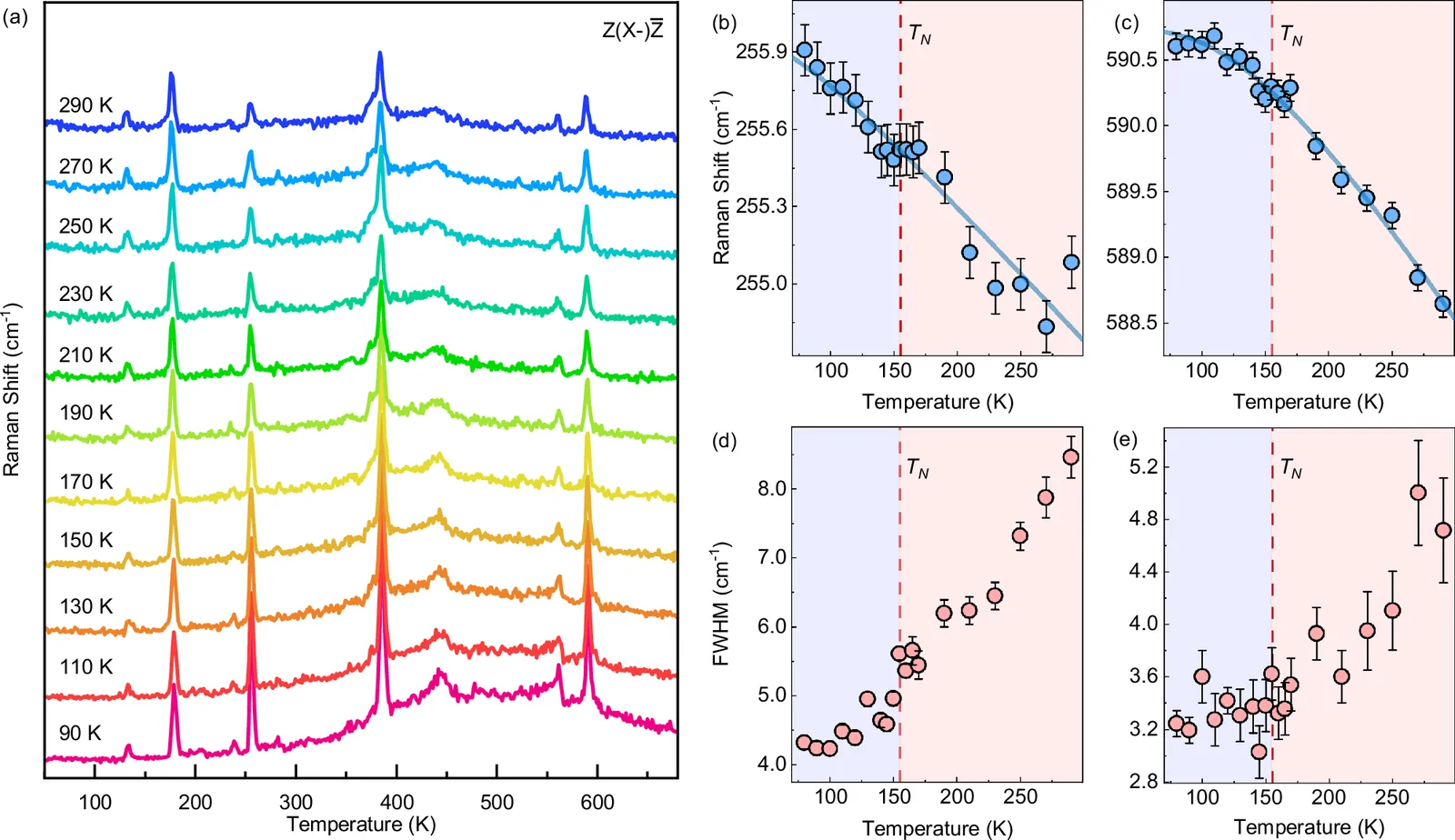
Temperature-dependent evolution of optical phonon modes in NiPS_3_. **a** Raman spectra collected in the $z\left(x-\right)\bar{z}$ polarization configuration from 90 to 290 K. **b**, **c** Temperature evolution of the phonon frequencies for the modes centered at ~255 cm^-1^ and ~588 cm^-1^, respectively. The solid curves represent fits to the anharmonic model, and the vertical dashed line marks the Néel temperature, ${T}_{N}$. **d**, **e** Full width at half maximum (FWHM) of the same two modes as a function of temperature. A small deviation from the anharmonic trend is observed near ${T}_{N}$ for the 255 cm^−1^ mode, whereas the 588 cm^−1^ mode exhibits no detectable anomaly. The error bars represent the uncertainty in the fitted peaks.

To assess the conventional spin-phonon coupling, we performed temperature-dependent Raman spectroscopy across ${T}_{N}$. Figure 2a shows the accumulated Raman spectra in the temperature range of 90 K to 290 K. A slight Fano asymmetry is visible in the ~255 cm^−1^ mode, consistent with earlier reports^16^ linking this feature to coupling between phonons and a magnetic excitation continuum^16,30^. Figure 2b–e presents the temperature dependence of the peak positions and linewidths for two representative modes at 255 cm^−1^ and 588 cm^−1^. As stated previously, the low-frequency modes primarily involve the vibration of Ni atoms, where the magnetic moments are localized^9^, whereas the higher frequency modes originate mainly from internal vibrations of P_2_S_6_ units. In principle, one should expect the low-frequency modes to be the most sensitive to magnetic ordering. However, the 255 cm^−1^ mode exhibits only a slight and narrow flattening around ${T}_{N}$ before resuming its anharmonic behavior (Fig. 2b), and its FWHM deviates by only ~0.3 cm^−1^ from the anharmonic trend. The higher-frequency mode at 588 cm^-1^ follows the expected anharmonic temperature evolution throughout the entire examined range with no detectable signature of magnetic ordering. The spectral position and FWHM of other peaks observed in Fig. 2a are provided in Supplementary Fig. 3. All observed changes for other peaks remain within fitting and instrumental uncertainty and therefore do not constitute a clear or systematic deviation at the transition. Prior polarization-resolved Raman studies have reported a small splitting of the peak at ~176 cm^−1^ across ${T}_{N}$ for NiPS_3_^25^ and also for other MPX_3_ families^36^, but our polarization-resolved measurements show no such splitting (Supplementary Fig. 4). The absence of a clearly resolved splitting in our measurements may be attributed, in part, to the limited spectral resolution and temperature range. Furthermore, the visibility of such subtle splitting can depend sensitively on experimental factors, including sample thickness, residual strain, and stacking faults, all of which can influence symmetry breaking and the corresponding Raman response. These observations suggest that the renormalization of the optical phonon frequencies driven by the onset of long-range magnetic order is small in NiPS_3_, and that other manifestations of spin-phonon coupling, such as changes in peak line-shape and intensity, are subtle and require higher spectral sensitivity to resolve reliably. Because the optical phonons do not provide a sufficiently sensitive probe of the spin–phonon coupling in NiPS_3_, we next examine the low-energy surface acoustic phonons using BMS spectroscopy, which directly probes magnetoelastic interactions.

**Fig. 3:**
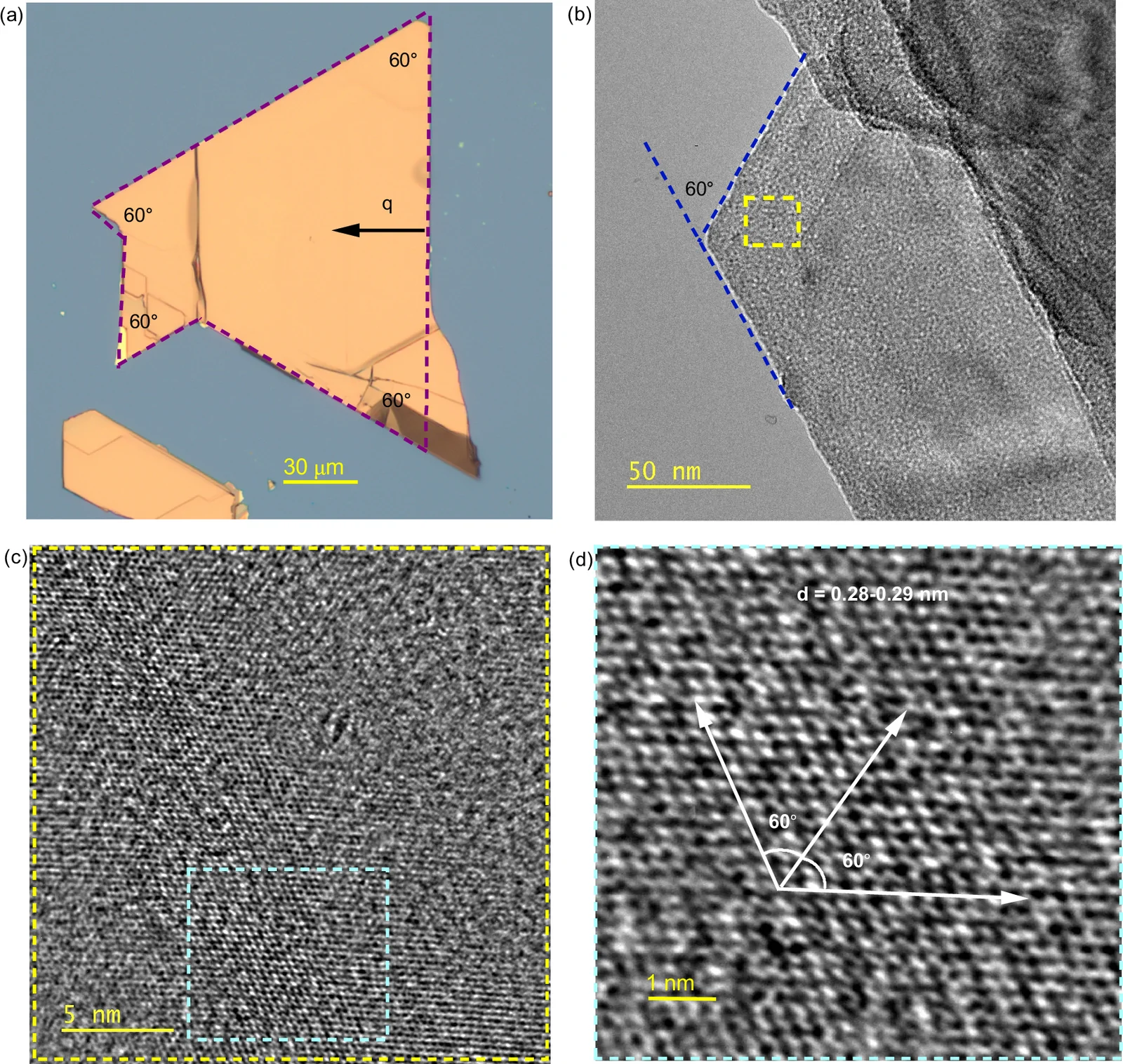
Morphology and crystallinity of NiPS_3_. **a** Optical image of an exfoliated NiPS_3_ flake on a SiO_2_/Si substrate. The lateral dimensions exceed 100 µm × 100 µm, and characteristic crystal cleavage angles of ~60° are indicated. The arrow labeled ${{\bf{q}}}$ denotes the in-plane wavevector of the surface acoustic phonon probed in the Brillouin–Mandelstam scattering (BMS) experiment. **b** TEM image of the liquid-phase exfoliated flake on the TEM grid, confirming the ~ 60° angular relationship. The region highlighted by the yellow dashed square is shown in (**c**), while the blue dashed region corresponds to (**d**). **c** High-resolution TEM image of the selected area. **d** Magnified lattice-resolved image with arrows indicating the (200), (130), and ($1\bar{3}0$) crystallographic planes with the distance *d *= 0.28–0.29 nm, forming ~60° angles consistent with the crystal symmetry.

Generally, understanding the crystallographic orientation of exfoliated films is important for surface acoustic phonon studies since it determines the probed phonon wavevector direction. The morphology and crystallinity of the exfoliated NiPS_3_ flakes are shown in Fig. 3. The optical image (see Fig. 3a) reveals large-area flakes with lateral dimensions exceeding 100 µm, exhibiting well-defined edges with characteristic ~60° angles, consistent with the underlying crystal symmetry. This angular relationship is further confirmed by high-resolution transmission electron microscopy (HR-TEM) imaging on a separate sample prepared via liquid-phase exfoliation (Fig. 3b). The high-resolution TEM images (Fig. 3c, d) resolve the lattice fringes with spacings of *d* = 0.28–0.29 nm and relative angles of ~60°, consistent with the flake geometry observed under optical microscopy. Based on these observations, edges forming 60° (or 120°) angles can be assigned to the $\left(200\right),$ which is aligned along the *b*-axis, ($1\bar{3}0$), and (130) planes, in agreement with prior TEM studies of NiPS_3_^37^. The phonon wavevector, **q**, in our measurements is oriented perpendicular to one of these edges. We performed detailed angle-resolved Raman measurements on the exfoliated samples, rotating the sample in a way that the polarization of the incident light is parallel to one of these edges. The details of the measurement and analysis are provided in Supplementary Section 4 (see Supplementary Figs. 5 and 6). The Raman response of the ${A}_{{{\rm{g}}}}$ modes, which shows nearly angle-independent intensity in parallel polarization (see Supplementary Fig. 5) and the suppression of their intensity in the cross-polarized signal, indicate that the anisotropy of the phonon modes within the *ab* plane is weak. This observation suggests that the phonon properties probed in our measurements exhibit only weak directional dependence in the *ab* plane, which will be further discussed next.

We performed BMS experiments in the conventional backscattering geometry to probe the low-energy acoustic phonons near the Brillouin-zone center. Similar to Raman spectroscopy, BMS relies on inelastic light scattering, but the energy range accessed is several orders of magnitude lower, corresponding to acoustic phonons with wavevectors close to the *Γ*-point. Further details of the technique can be found in prior work from our group and others^38–40^. Because the thickness of our exfoliated NiPS_3_ thin films (*h* = 220 nm, 260 nm) is smaller than the BMS laser excitation wavelength, λ=532 nm, the BMS spectra are dominated by scattering from surface acoustic waves^38,39^. In this thickness regime, the out-of-plane component of the phonon momentum is not uniquely defined, and only the in-plane component of momentum is conserved in the scattering process^39^. The in-plane phonon wavevector is given by ${{\bf{q}}}=(4\pi /\lambda )\sin \theta$, where θ is the angle of incidence measured relative to the surface normal. The in-plane phonon wavevector, ${{\bf{q}}}$, was aligned perpendicular to one of the film’s edges (Fig. 3a). Note that we performed additional BMS experiments in which the phonon wavevector, **q**, was oriented perpendicular to each of the three cleaved crystal edges in each measurement (Supplementary Fig. 7a). The results show no significant variation in the phonon phase velocity along these directions (Supplementary Fig. 7b), further confirming the weak anisotropy in the *ab* plane. Consequently, for surface phonon measurements, the exact in-plane orientation of the phonon wavevector ${{\bf{q}}}$ is of limited importance, and reliable information can be obtained without precise identification of its crystallographic direction. For this reason, after fixing the in-plane orientation, we varied θ to tune different magnitudes of ${{\bf{q}}}$ and extract the dispersion, i.e., energy vs. ${{\bf{q}}}$, of the surface acoustic mode.

**Fig. 4:**
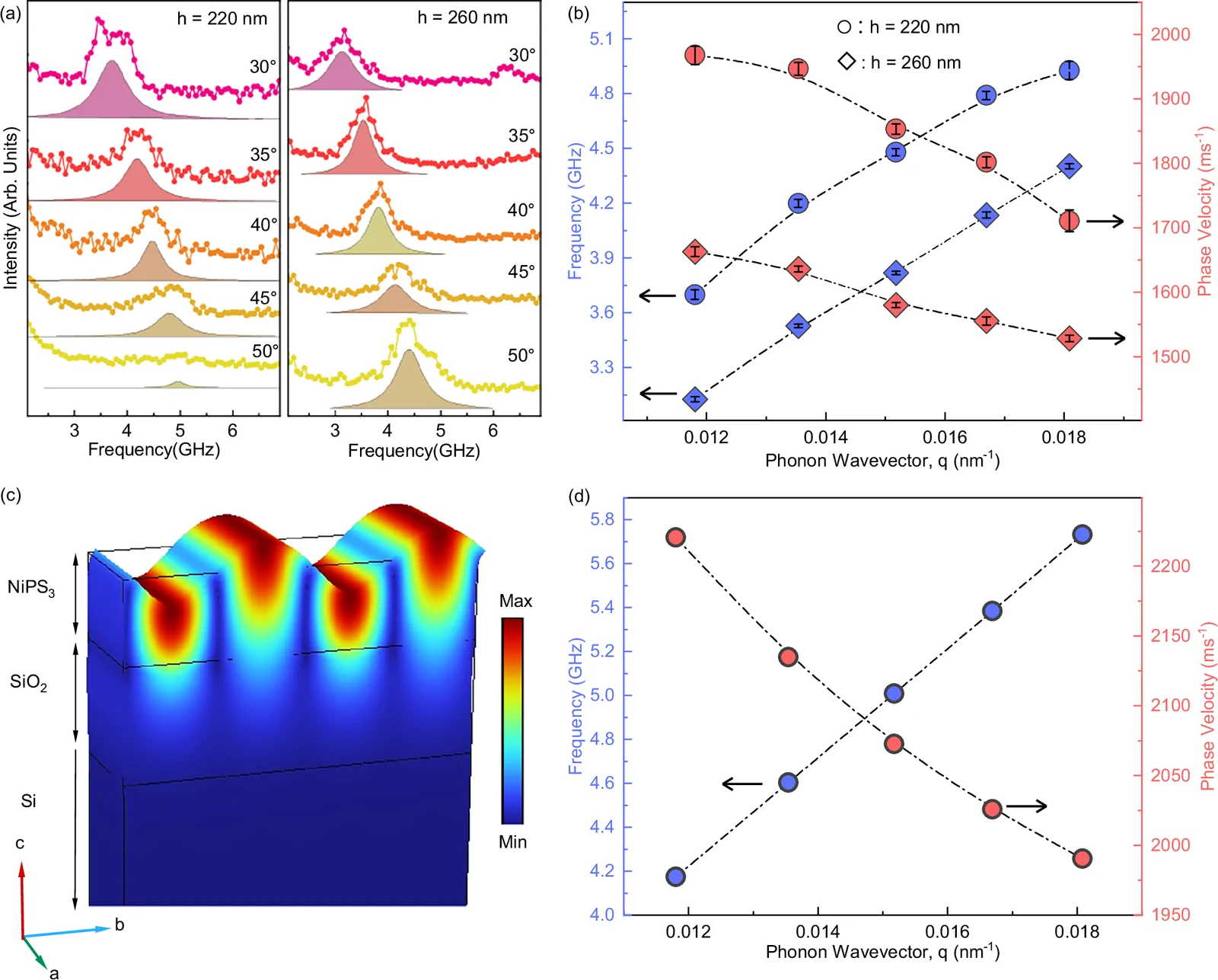
Experimental and theoretical Rayleigh-wave dispersion in NiPS_3_. **a** BMS spectra of the exfoliated NiPS_3_ film with the thickness of *h* = 220 nm and 260 nm collected at room temperature for incidence angles between 30° and 50°. A single dispersive peak is observed and assigned to the surface Rayleigh wave (RW). The shaded areas represent the individual Lorentzian fits used to extract the peak frequencies. **b** Spectral position, f, of the RW peak (blue symbols) obtained by Lorentzian fitting, together with the corresponding phase velocity (red symbols) as a function of wavevector, ${{\bf{q}}}$. Circle symbols denote the 220 nm film, while diamond symbols represent the 260 nm film. The dashed line marks the trend of decreasing phase velocity with increasing ${{\bf{q}}}$. The error bars correspond to the uncertainty in the fitted peak position. **c** Displacement field of the RW mode calculated using the continuum elastic model at ${{\bf{q}}}=0.0135\,{{\rm{n}}}{{{\rm{m}}}}^{-1}$ for the film with the thickness of 220 nm, showing surface-localized, elliptical motion predominantly within the NiPS_3_ layer. **d** Simulated RW dispersion and phase velocity in the non-magnetic (NM) state, extracted from finite-element eigenmode analysis. The simulated trend shows good agreement with the experimental results in (**b**).

The accumulated spectra obtained for θ ranging from 30° to 50° at RT for the films with the thicknesses, *h*, of 220 nm and 260 nm are shown in Fig. 4a. We identified a single well-defined dispersive peak, whose frequency increases with ${{\bf{q}}}$. This peak is attributed to the Rayleigh surface acoustic wave in NiPS_3_. To verify that the observed mode originates from the NiPS_3_ layer rather than the underlying SiO_2_/Si substrate, we also measured the BMS spectra of a bare SiO_2_/Si substrate under the same experimental geometry (Supplementary Fig. 8). The substrate exhibits multiple features associated with Rayleigh, Sezawa, and Lamb surface acoustic waves, consistent with the literature^41^. These modes exhibit different phase velocities and spectral positions than the single dispersive peak observed in NiPS_3_, confirming that the latter originates from the Rayleigh wave in the exfoliated NiPS_3_ layer. The assignment of this mode will be further corroborated through comparison with calculated surface acoustic wave dispersions later.

The spectral position, f, of this peak at different $\theta {{\rm{s}}}$ was obtained by fitting the experimental data with individual Lorentzian functions (shaded area in Fig. 4a). The resulting values are plotted as a function of the corresponding ${{\bf{q}}}$, as shown by blue circles and diamonds for h= 220 nm and h= 260 nm, respectively, in Fig. 4b. The phase velocity was then calculated using v=2πf/q, and is depicted by red circles and diamonds for respective thickness on the same plot. Note that, in thin films, the spectral position and phase velocity of Rayleigh waves are expected to depend on film thickness, a trend that is clearly reflected in our experimental results. The dispersion of the Rayleigh surface wave in a layered structure depends on whether the film is slower or faster than the substrate with respect to the relevant in–plane acoustic velocities^38,42^. In a slow-on-fast configuration, where the film has a lower in-plane shear acoustic velocity than the substrate, the Rayleigh mode at long wavelengths (small ${{\bf{q}}}$) penetrates into the substrate and therefore exhibits a higher effective phase velocity. As ${{\bf{q}}}$ increases, the acoustic wavelength becomes shorter and the surface wave becomes progressively more confined to the NiPS_3_ layer, reducing substrate participation. The decreasing phase velocity of the peak with increasing ${{\bf{q}}}$, shown in Fig. 4b for both thicknesses indicates that the NiPS_3_/SiO_2_/Si heterostructure behaves effectively as a slow-on-fast system for the surface acoustic wave.

To identify the nature of the observed dispersive mode, we calculated the elastic stiffness tensor, ${c}_{{ij}}$, of NiPS_3_ using density functional theory (DFT) with PBE-D3 correction in both the AFM and non-magnetic (NM) states. Since NiPS_3_ is paramagnetic (PM) above ${T}_{N}$, the NM state is used as an approximation to the high-temperature PM phase. The resulting elastic constants were incorporated into a continuum elastic model of the NiPS_3_/SiO_2_/Si heterostructure implemented using finite-element simulations in COMSOL Multiphysics. Additional computational details are provided in the Methods, and validation of the simulation setup is checked with SiO_2_/Si configuration shown in Supplementary Fig. 9. The eigenmode analysis yields several surface and guided acoustic branches, but only one mode displays surface-localized elliptical displacement predominantly within the NiPS_3_ layer (Fig. 4c), which is the characteristic vibrational signature of a Rayleigh surface acoustic wave. According to Loudon’s selection rule for surface Brillouin scattering, only modes with displacement components perpendicular to the phonon propagation direction contribute significantly to the scattered intensity^39,43,44^. Therefore, this Rayleigh mode is expected to dominate the BMS spectrum. The calculated frequency of this mode for sample with h= 220 nm at ${{\bf{q}}}=$ 0.0135 nm^−1^ is ∼4.6 GHz, which is in close agreement with the experimentally observed value of ∼4.2 GHz. We also calculated the dispersion and phase velocity of this surface mode, shown with blue and red circles, respectively, in Fig. 4d. As seen, the theoretical calculations follow the same decreasing phase-velocity trend observed experimentally in Fig. 4b. This agreement between experiment and modeling further validates the mode assignment and indicates that the Rayleigh wave becomes increasingly confined to the NiPS_3_ layer as the in-plane wavevector increases (Supplementary Fig. 10).

**Fig. 5:**
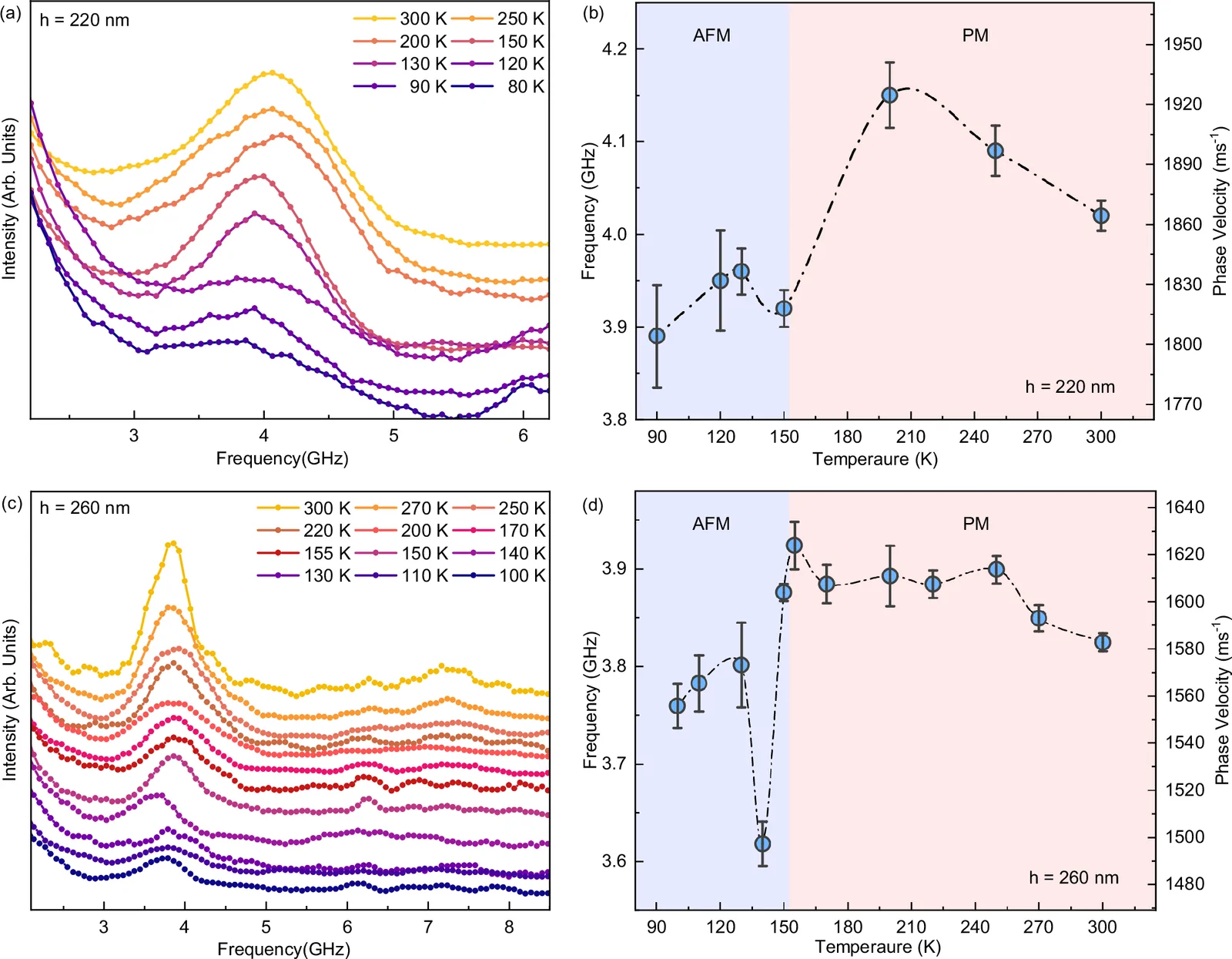
Temperature dependence of the Rayleigh surface acoustic wave in NiPS_3_. **a**, **c** BMS spectra of NiPS_3_ film exfoliated on SiO_2_/Si substrate measured between 300 K and 80 K at constant wavevector ${{\bf{q}}}=0.0135\,{{\rm{n}}}{{{\rm{m}}}}^{-1}$ and ${{\bf{q}}}=0.0152\,{{\rm{n}}}{{{\rm{m}}}}^{-1}$ for films with a thickness of 220 nm and 260 nm, respectively. Upon cooling, the Rayleigh-wave frequency initially increases due to lattice stiffening, then exhibits a pronounced drop at 150 K near the Néel temperature (~155 K), followed by saturation at lower temperatures. **b**, **d** Show the peak frequency on the left axis and the phase velocity of the Rayleigh-wave on the right axis as a function of temperature for the films with a thickness of 220 nm and 260 nm, respectively. The velocity in the 220 nm film decreases from ~1920 ${{\rm{m}}}{{{\rm{s}}}}^{-1}$ in the paramagnetic (PM) phase to ~1815 ${{\rm{m}}}{{{\rm{s}}}}^{-1}$ in the antiferromagnetic AFM phase, corresponding to a ~ 6% reduction across the transition, while in 260 nm film it reduced by ~7.3%. The shaded regions mark the PM and AFM phases. The anomaly in velocity reflects the magnetoelastic softening associated with the onset of zigzag AFM order in NiPS_3_. The error bars correspond to the uncertainty in the fitting.

The Rayleigh surface acoustic wave is governed by the elastic constants of the crystal, making its frequency highly sensitive to changes in lattice stiffness associated with magnetic ordering^45,46^. Therefore, the temperature dependence of the Rayleigh mode provides a direct probe of magnetoelastic coupling across the AFM–PM transition. To track the Rayleigh wave evolution with temperature, BMS measurements were conducted from 300 K to 80 K while maintaining a constant in-plane wavevector, ${{\bf{q}}}$, for each sample. Due to the highly time-intensive nature of temperature-dependent BMS measurements, we first investigated the 220 nm-thick NiPS_3_ flake using relatively large temperature intervals. The obtained spectra, accumulated at a constant incident angle of $\theta=35^\circ$, corresponding to the wavevector ${{\bf{q}}}=0.0135\,{{\rm{n}}}{{{\rm{m}}}}^{-1}$, are shown in Fig. 5a. These measurements revealed a pronounced softening of the Rayleigh mode in the vicinity of the Néel temperature at ~150 K. To verify the reproducibility of this behavior and resolve the transition with higher precision, we repeated the measurements on a 260 nm-thick flake using a much denser temperature grid around the magnetic transition. The measurements were conducted at $\theta=40^\circ$ corresponding to ${{\bf{q}}}=0.0152\,{{\rm{n}}}{{{\rm{m}}}}^{-1}$. The obtained spectra are presented in Fig. 5c. Each spectrum in Fig. 5a, c was fit using a single Lorentzian function. As seen, the spectral position of the Rayleigh peak increases gradually upon cooling from RT to ~200 K, followed by a distinct drop at around ${T}_{N}$, and then remains nearly constant at lower temperatures. The initial increase reflects the expected reduction of anharmonic lattice vibrations upon cooling, which leads to lattice stiffening and thus a higher acoustic phase velocity. The drop in frequency at ~150 K is associated with the onset of AFM order, where spin rearrangement modifies the elastic constants through magnetoelastic coupling, resulting in a measurable softening. Note that although the two datasets may appear somewhat different at first glance, this primarily reflects the different temperature intervals used during data acquisition rather than an intrinsic difference in the underlying behavior. The more sparsely sampled 220 nm dataset gives the impression of a broader anomaly, whereas the finer temperature resolution of the 260 nm measurements resolves the same feature as a sharper transition. Nevertheless, closer inspection shows that the two datasets exhibit remarkably similar quantitative behavior, with phonon hardening above ${T}_{N}$ of approximately 0.13 GHz for the 220 nm and 0.10 GHz for the 260 nm flake and magnetoelastic softening across the transition of approximately 0.23 GHz and 0.30 GHz for the 220 nm and 260 nm films, respectively. Both samples show nearly temperature-independent frequencies deep in the antiferromagnetic phase with only ~0.05–0.07 GHz variation. The similar softening of the Rayleigh mode is quantitatively reflected in the phase velocity of the Rayleigh waves, plotted in Fig. 5b, d, which decreases from ~1920 ${{\rm{m}}}{{{\rm{s}}}}^{-1}$ in the PM state to ~1815 ${{\rm{m}}}{{{\rm{s}}}}^{-1}$ in the AFM state, corresponding to a ~ 6 % reduction across the transition for the 220 nm film. The phase velocity for 260 nm film reduces from 1610 ${{\rm{m}}}{{{\rm{s}}}}^{-1}$ to 1500 ${{\rm{m}}}{{{\rm{s}}}}^{-1}$ which corresponds to ~7.3 % decrease in phase velocity. Once the magnetic order is established, the elastic constants stabilize, resulting in a nearly temperature-independent frequency at low temperatures. These results further support the weak anisotropy nature of the basal plane, as the phonon wavevector – probed perpendicular to random crystal edges in the two films—yields phonon velocity softening that varies by only ~1%.

To further understand the origin of the Rayleigh-wave anomaly across the magnetic transition, we extended our calculation of the elastic stiffness tensor ${c}_{{ij}}$ to the AFM ground state and compared it with the previously obtained NM phase used in our simulations. The ${c}_{{ij}}$ tensors in both phases are summarized in Table 1 in standard Voigt notation. NiPS_3_ with a monoclinic C2/m structure possesses 13 independent elastic constants. As seen in the table, several ${c}_{{ij}}$ elements undergo pronounced changes upon magnetic ordering. The most notable variation occurs in the in-plane shear stiffness ${c}_{66}$, which increases from 47.4 GPa in the NM phase to 55.4 GPa in the AFM phase. In contrast, interlayer and in-plane coupling terms such as ${c}_{12}$ and ${c}_{23}$ decrease, reflecting a magnetically driven softening of off-diagonal couplings. Note the sign reversal of the shear–normal coupling constant ${c}_{35}$, which changes from –0.5 GPa (NM) to +0.4 GPa (AFM). Because the Rayleigh surface wave involves a mixed displacement of in-plane longitudinal (LA) and out-of-plane transverse (TA) components, its frequency is governed by a complex combination of elastic coefficients rather than a single stiffness term^46^. In an anisotropic heterostructure such as NiPS_3_/SiO_2_/Si, additional boundary constraints and elastic mismatch further influence the effective velocity^46^. To quantify this effect, we implemented the AFM-phase elastic constants in finite-element simulations of the continuum elastic model in COMSOL Multiphysics. The calculated Rayleigh mode at ${{\bf{q}}}=0.0135\,{{\rm{n}}}{{{\rm{m}}}}^{-1}$ shows a frequency of 4.578 GHz, corresponding to a 0.54 % decrease relative to the NM-phase value of 4.603 GHz. Although this reduction is smaller than the experimentally observed drop (~6 %), the trend confirms that the AFM ordering leads to elastic softening of the Rayleigh mode. The smaller calculated change likely arises because DFT captures both AFM and NM phases at 0 K, whereas finite-temperature effects further renormalize the elastic response in experiments. Additionally, the PM phase was approximated assuming NM configuration, which neglects spin fluctuations and thus introduces additional uncertainty into the comparison.

**Table 1:** Elastic constant ${c}_{{ij}}$ (GPa) of NiPS_3_ obtained from DFT calculation

	${{{\boldsymbol{c}}}}_{{{\boldsymbol{11}}}}$	${{{\boldsymbol{c}}}}_{{{\boldsymbol{22}}}}$	${{{\boldsymbol{c}}}}_{{{\boldsymbol{33}}}}$	${{{\boldsymbol{c}}}}_{{{\boldsymbol{12}}}}$	${{{\boldsymbol{c}}}}_{{{\boldsymbol{13}}}}$	${{{\boldsymbol{c}}}}_{{{\boldsymbol{23}}}}$	${{{\boldsymbol{c}}}}_{{{\boldsymbol{44}}}}$	${{{\boldsymbol{c}}}}_{{{\boldsymbol{55}}}}$	${{{\boldsymbol{c}}}}_{{{\boldsymbol{66}}}}$	${{{\boldsymbol{c}}}}_{{{\boldsymbol{15}}}}$	${{{\boldsymbol{c}}}}_{{{\boldsymbol{25}}}}$	${{{\boldsymbol{c}}}}_{{{\boldsymbol{35}}}}$	${{{\boldsymbol{c}}}}_{{{\boldsymbol{46}}}}$
NM	152.3	148.4	44.8	50.7	9.0	12.3	9.7	9.9	47.4	3.4	−4.2	−0.5	−2.5
AFM	150.2	151.2	41.3	39.5	8.4	9.4	9.7	10.1	55.4	2.1	−1.9	0.4	−2.1

From an atomistic perspective, the contrast between the weak, mode-selective frequency shifts of optical phonons and the pronounced softening of the Rayleigh surface acoustic wave reflects two distinct forms of spin–lattice coupling. Optical phonons involve out-of-phase atomic displacements within the unit cell that locally perturb super-exchange pathways^20^. Their magnetic renormalization is governed by the derivative of the exchange integral with respect to an atomic displacement ${u}_{\alpha }$, such that the optical phonons frequency shift obeys $\Delta {\omega }_{{opt}}\propto \left(\partial {J}_{{ij}}/\partial {u}_{\alpha }\right) < {S}_{i}.{S}_{j}{{\boldsymbol{ > }}}$^15,19,47^. Here, ${J}_{{ij}}$ is the exchange interaction between spins ${S}_{i}$ and ${S}_{j}$. Because different bonds contribute with opposite signs of $\partial {J}_{{ij}}/\partial {u}_{\alpha }$, these effects are strongly mode-dependent and can yield either softening or hardening across the transition^15,25^. In contrast, acoustic and Rayleigh waves correspond to in-phase, long-wavelength displacements that generate a slowly varying strain field ${\varepsilon }_{\alpha \beta }=(1/2)[\left(\partial {u}_{\alpha }/\partial {x}_{\beta }\right)+\left(\partial {u}_{\beta }/\partial {x}_{\alpha }\right)]$ [ref. ^48^]. This coherently modulates all exchange interactions through ${J}_{{ij}}={J}_{{ij}}^{(0)}+(\partial {J}_{{ij}}/\partial {\varepsilon }_{\alpha \beta }){\varepsilon }_{\alpha \beta }$, giving rise to a magnetoelastic energy density ${E}_{{me}}=-{\sum}_{\alpha \beta }{B}_{\alpha \beta }{\varepsilon }_{\alpha \beta }{S}_{\alpha }{S}_{\beta }$, where ${B}_{\alpha \beta }$ quantifies the collective derivative $\partial {J}_{{ij}}/\partial {\varepsilon }_{\alpha \beta }$ [ref. ^49^]. When long-range AFM order sets in, this coupling renormalizes the elastic tensor as ${c}_{\alpha \beta }^{{eff}}={c}_{\alpha \beta }^{(0)}-{\lambda }_{\alpha \beta }^{2}{\chi }_{m}$, with ${\chi }_{m}$ the magnetic susceptibility and ${\lambda }_{\alpha \beta }\propto {B}_{\alpha \beta }$. Consequently, the Rayleigh-wave velocity, ${v}_{R}\propto \sqrt{{c}_{{eff}}/\rho }$ decreases by ${\Delta }{v}_{R}/{v}_{R}\simeq 6\%$, implying an effective modulus reduction of $\Delta {c}_{{eff}}/{c}_{{eff}}\simeq (2)(\Delta {v}_{R}/{v}_{R})\simeq 12\%$. This quantitatively links the macroscopic elastic softening to the same exchange–striction mechanisms responsible for optical phonon renormalization but now integrated coherently over the entire lattice. The observed Rayleigh anomaly thus represents the continuum limit of spin–phonon coupling, where the collective modulation of exchange by uniform strain yields a measurable renormalization of the elastic constants across the magnetic transition.

The substantial softening of the Rayleigh-wave velocity across the AFM–PM transition demonstrates that the acoustic elastic response of NiPS_3_ is strongly renormalized by magnetic ordering. Since the Rayleigh mode in the NiPS_3_/SiO_2_/Si heterostructure depends on a complex combination of elastic constants of the film and the substrate, we performed a systematic sensitivity analysis by individually increasing each of the 13 independent ${c}_{{ij}}$ values by 20 % in the continuum elastic model while keeping all others fixed (see Table 2). The calculated response revealed that the Rayleigh-wave velocity is most sensitive to ${c}_{33}$ and ${c}_{44}$, with weaker contributions from ${c}_{22}$, ${c}_{23}$, and ${c}_{46}$, whereas the remaining elastic constants have a negligible impact. The experimentally observed ∼6 % drop in phase velocity at ${T}_{N}$ therefore implies a significant reduction in the shear- and out-of-plane-related elastic coefficients arising from magnetoelastic renormalization triggered by the emergence of zigzag antiferromagnetic order. The observation is consistent with the previously reported spin-shear deformation in NiPS_3_ [ref. ^50^].

**Table 2:** Sensitivity analysis of the 13 independent elastic constants (${c}_{{ij}}$) of monoclinic NiPS_3_

20 % increase in:	${{{\boldsymbol{c}}}}_{{{\boldsymbol{11}}}}$	${{{\boldsymbol{c}}}}_{{{\boldsymbol{22}}}}$	${{{\boldsymbol{c}}}}_{{{\boldsymbol{33}}}}$	${{{\boldsymbol{c}}}}_{{{\boldsymbol{12}}}}$	${{{\boldsymbol{c}}}}_{{{\boldsymbol{13}}}}$	${{{\boldsymbol{c}}}}_{{{\boldsymbol{23}}}}$	${{{\boldsymbol{c}}}}_{{{\boldsymbol{44}}}}$	${{{\boldsymbol{c}}}}_{{{\boldsymbol{55}}}}$	${{{\boldsymbol{c}}}}_{{{\boldsymbol{66}}}}$	${{{\boldsymbol{c}}}}_{{{\boldsymbol{15}}}}$	${{{\boldsymbol{c}}}}_{{{\boldsymbol{25}}}}$	${{{\boldsymbol{c}}}}_{{{\boldsymbol{35}}}}$	${{{\boldsymbol{c}}}}_{{{\boldsymbol{46}}}}$
Changes in phase velocity (%)	0	+0.47	+1.94	0	0	−0.14	+4.85	0	0	0	0	0	+0.15

Recent studies have identified interlayer shear as a primary structural degree of freedom that couples directly to the magnetic order parameter and exhibits critical behavior near ${T}_{N}$ [refs. ^50,51^]. In this framework, the elastic softening inferred from our measurements can be understood as a continuum-scale manifestation of this spin–shear coupling, where the same interaction that drives shear distortions and dynamical critical behavior also renormalizes the effective elastic constants governing long-wavelength acoustic modes^50^. Thus, the previously reported spin–shear deformation and the elastic softening observed here represent two complementary aspects of the same underlying spin–lattice interaction. In this picture, the magnetically driven shear distortions modify the elastic tensor, which in turn renormalizes the velocity of long-wavelength acoustic modes such as the Rayleigh wave measured in our BMS experiment. These results show that the spin-driven reconfiguration of the elastic tensor in NiPS_3_ is sufficiently large to significantly alter the propagation of surface acoustic phonons, revealing pronounced spin–lattice interaction at long wavelengths. Materials such as FePS_3_, CrBr_3_, and Cr_2_Ge_2_Te_6_ have been shown to exhibit strong spin–lattice coupling manifested through optical phonon renormalization, magnon–phonon hybridization, and pronounced thermal-expansion anomalies^52–58^. In contrast, the Rayleigh-wave anomaly observed here highlights a complementary manifestation of spin–lattice coupling in NiPS_3_ in the long-wavelength acoustic (continuum) regime, providing a direct long-wavelength spectroscopic signature of spin–phonon coupling. Our findings establish NiPS_3_ as a promising platform for studying magnetoelastic interactions and phonon-mediated functionalities in low-dimensional antiferromagnets.

In summary, we have demonstrated that long-wavelength acoustic phonons provide a uniquely sensitive probe of spin–lattice coupling in the quasi-two-dimensional antiferromagnet NiPS_3_. Our Brillouin–Mandelstam measurements reveal a pronounced softening of the Rayleigh surface mode across the Néel temperature, corresponding to ~6% to ~7% reduction in phase velocity. The latter directly demonstrates that the onset of zigzag AFM order renormalizes the long-wavelength elastic constants. First-principles calculations of the elastic tensor, together with continuum modeling, corroborate the magnetic-order–induced changes in elasticity and link them to exchange-striction at the microscopic level. These results establish the continuum limit of spin–phonon interactions in NiPS_3,_ where microscopic exchange interactions coherently reshape the macroscopic stiffness of the lattice. By revealing how magnetic order governs the elastic energy landscape, this work provides a foundation for lattice-controlled spin phenomena and offers opportunities for magnetically tunable phononic, magnonic, and strain-mediated spintronic devices in low-dimensional materials.

## Methods

### Raman spectroscopy measurements

Raman spectroscopy measurements were performed on exfoliated NiPS_3_ film using a Renishaw inVia Qontor system with a λ= 532 nm excitation laser over the temperature range from 77 K to 300 K. The measurements were conducted in backscattering geometry. The scattered light was collected under different polarization configurations to distinguish the vibrational symmetries of the phonon modes. The spectrometer was calibrated before each measurement using an undoped SiO_2_/Si wafer, with the Si peak fixed at 520 cm^−1^. A low laser power of 0.024 mW was used to ensure a high signal-to-noise ratio while minimizing laser-induced heating of the sample.

### Brillouin-Mandelstam spectroscopy measurements

The Brillouin-Mandelstam spectroscopy measurements were performed in a backscattering geometry using a continuous-wave solid-state diode-pumped laser (Spectra-Physics, Excelsior, USA) at the excitation wavelength of λ= 532 nm. The scattered light was directed into a high-resolution, high-contrast 3 + 3 tandem Fabry-Perot interferometer (The Table Stable, Switzerland), coupled to a detector and spectrum analyzer.

### Density functional theory (DFT) calculations

All first-principles calculations were performed within the framework of DFT using the Vienna ab initio Simulation Package (VASP)^59–61^. The plane-wave energy cutoff was set to 600 eV. Gaussian smearing method with a smearing width of 0.01 eV was used. The exchange–correlation functional was treated using the generalized-gradient approximation in the PBE form^62^, augmented with Grimme’s D3 correction to account for long-range van der Waals interactions^63^. Structural relaxations were performed with force and total-energy convergence thresholds of 0.005 eV/Å and 1 × 10−^9^ eV, respectively. Elastic constants were obtained from stress–strain relations using finite differences with a strain amplitude of 0.015 and four displacements. A Γ-centered *k*-point mesh of 3×2×3 was employed for Brillouin zone sampling. The simulations were carried out for a 1×4×2 supercell of NiPS_3_ (lattice dimensions (AFM): 11.499 × 19.914 × 12.308 Å), consistent with its monoclinic (C2/m) symmetry. All calculations were performed for both the AFM ground state and the NM (paramagnetic proxy) state, ensuring full relaxation in the respective magnetic configuration prior to elastic tensor evaluation. Phonon dispersion in the AFM and NM states is shown in Supplementary Figs. 11 and 12.

### Finite element simulations

Finite-element simulation was performed by using COMSOL Multiphysics with the Solid Mechanics Module. The system is modeled with the small-strain, linear electrodynamic equation in the frequency domain, $-\rho {\omega }^{2}u=\nabla .s$, where u is the displacement vector, ρ is the density, ω is angular frequency, and s is the stress tensor related to the strain by the stiffness tensor ${c}_{{ij}}$. Simulations were carried out in 3D by applying an eigenfrequency study to reflect the experimental excitation of the system. The geometric parameters used in the simulations were aligned with the actual characteristics of the samples studied experimentally. The model consisted of a rectangular block of NiPS_3_ layer of thickness 220 nm on top of a SiO_2_ layer (300 nm) over a Si half-space, represented numerically by a finite thickness. Floquet periodic boundary conditions (PBC) were applied along the x and y axes of the unit cell with a phase shift $u\left(x+{L}_{x}\right)=u\left(x\right){e}^{{ik}{L}_{x}},$ where $u\left(x\right)$ is the displacement along *x* direction, ${L}_{x}$ is the model length, and k is the periodic phase set to the experimentally studied phonon wavevector, **q**. These PBCs were implemented on both faces of the unit cell to ensure consistent values of the elastic tensor components and density throughout the entire modeled structure. The top surface of NiPS_3_ is set to be free and the bottom surface of the Si was fixed. NiPS_3_ was treated as a linear elastic, anisotropic solid.

## Supplementary information

Supplementary Information

Transparent Peer Review file

Peer Review File

## Data Availability

Any additional data is available from the authors upon request.
