# Supplementary material for: Anomalous spin–lattice coupling in a 2D antiferromagnetic semiconductor revealed by surface acoustic Rayleigh waves
Source: Nat Commun. 2026 Oct 12;17:10741. doi: 10.1038/s41467-026-77234-4 (PMC13661896; doi:10.1038/s41467-026-77234-4)
Supplement: Supplementary file 1 [file 41467_2026_77234_MOESM1_ESM.pdf]

Supporting Information

Anomalous spin–lattice coupling in a 2D antiferromagnetic semiconductor revealed by surface acoustic Rayleigh waves

Zahra Ebrahim Nataj,[1,2] Md. Sabbir Hossen Bijoy[3], Vladislav Korostelev[3], Jordan Teeter[1,2], Dylan Wright[1,2], Mohammad Zeinolabedini[3], Konstantin Klyukin[3], Fariborz Kargar [3,*], Alexander A. Balandin[1,2,*]

[1]Department of Materials Science and Engineering, University of California, Los Angeles, California 90095 USA

[2]California NanoSystems Institute, University of California, Los Angeles, California 90095 USA

[3]Materials Research and Education Center, Department of Mechanical Engineering, Auburn University, Auburn, Alabama 36849 USA

[*] Corresponding author: fkargar@auburn.edu (F.K.); balandin@seas.ucla.edu (A.A.B.)

Contents

1. Atomic force microscopy characterization 2
2. Raman peaks analysis at different polarization configurations 3
3. Temperature-dependent Raman spectroscopy of $NiPS_3$ film................................ 3
4. Angle-resolved Raman and BLS measurements on the exfoliated film 5
5. Brillouin–Mandelstam spectroscopy of bare SiO_2/Si substrate 9
6. Surface acoustic phonon simulation of SiO_2/Si substrate................................11
7. Surface acoustic phonon simulation for $NiPS_3$ at higher **q**................................11
8. Phonon dispersion for $NiPS_3$ 12

1. Atomic force microscopy characterization

AFM inspection of the exfoliated $NiPS_3$ sample was used to determine the film thickness. Supplementary Fig. 1 and 2 (a) shows the thin film imaged with AFM. Supplementary Fig. 1 and 2 (b) presents the topographic, i.e., height, AFM image acquired at the location indicated in Supplementary Fig. 1 and 2 (a), which shows the average thickness of 220 nm and 260 nm, respectively.

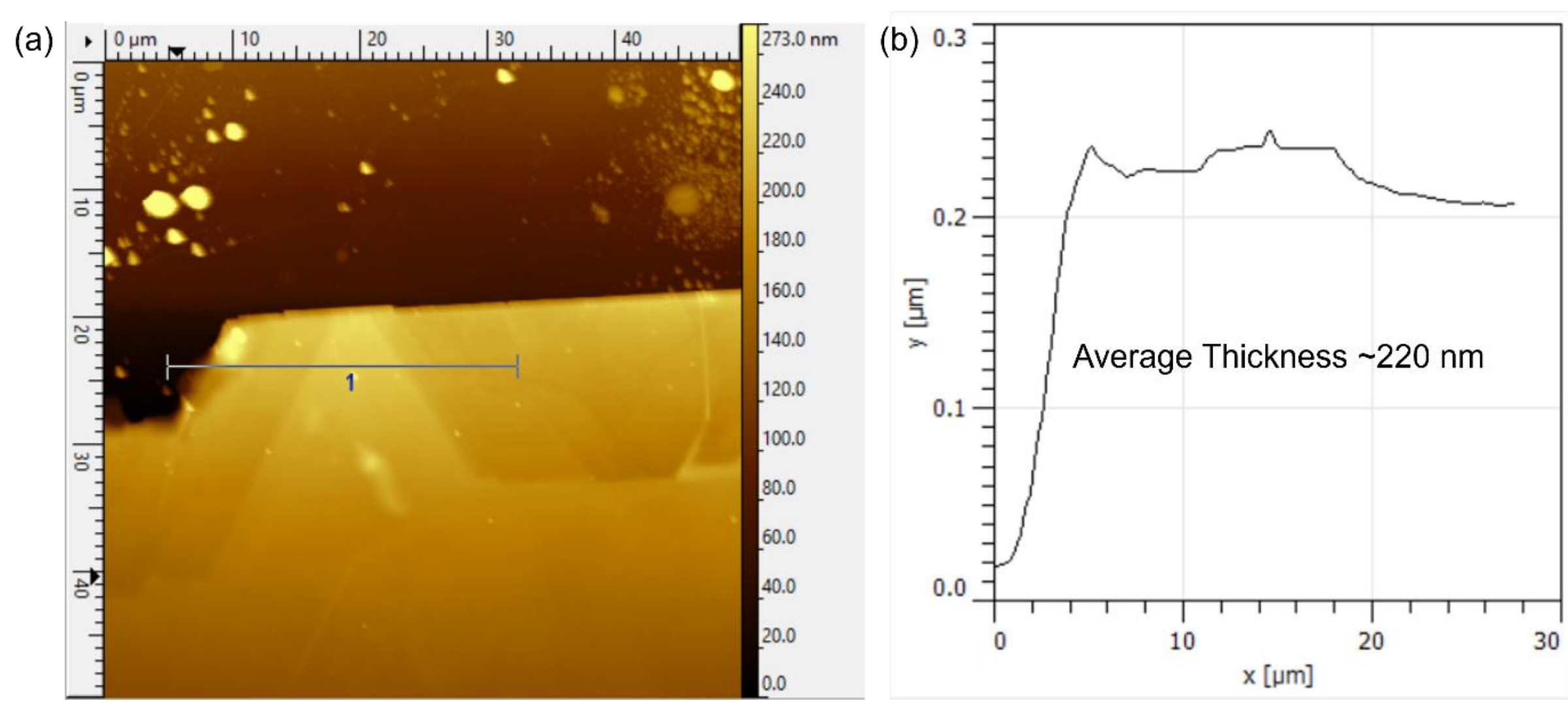

Supplementary Fig. 1: (a) AFM image of the $NiPS_3$ film with the indicated scan direction. (b) AFM height profile of the exfoliated film showing an average thickness of 220 nm.

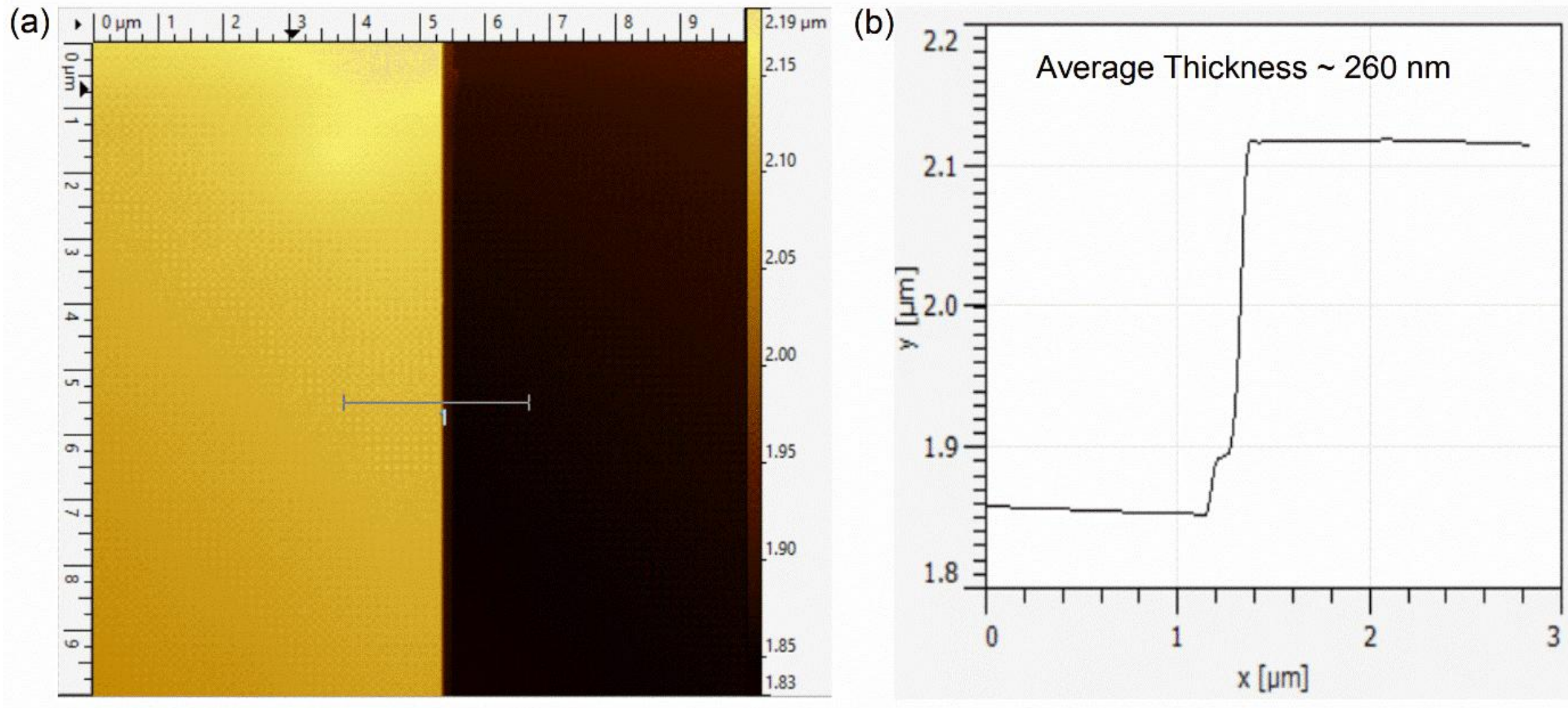

Supplementary Fig. 2: (a) AFM image of the $NiPS_3$ film with the indicated scan direction. (b) AFM height profile of the exfoliated film showing an average thickness of 260 nm.

2. Raman peaks analysis at different polarization configurations

The positions and the full-width-at-half-maximums (FWHM) of the observed Raman peaks in the different polarization configurations are listed in Supplementary Table 1.

Supplementary Table 1. Frequency and FWHM of Raman peaks at different polarization configurations.

	Symmetry		A_g, B_g	A_g, B_g	A_g	A_g, B_g	A_g	A_g, B_g	A_g
Polarization	$z(x\text{-})\bar{z}$	Frequency	131.6	176.3	254.7	281.6	383.1	559.5	588.2
		FWHM	6.3	4.4	4.7	9.4	9.1	5.5	4.2
	$z(xx)\bar{z}$	Frequency	131.9	176.3	254.7	282.2	383.5	559.4	588.6
		FWHM	5.7	4.3	4.8	6.8	7.4	7.7	4.8
	$z(xy)\bar{z}$	Frequency	131.5	176.22	-	280.8	-	559.7	-
		FWHM	4.4	4.2	-	3.9	-	5.1	-

3. Temperature-dependent Raman spectroscopy of $NiPS_3$ film

Supplementary Fig. 3 shows the temperature-dependent peak center and FWHM of the peaks at ~ 179 cm^{-1} and 386 cm^{-1}. In our measurements, the mode at 176 cm^{-1} exhibits a small change of ~0.3

cm-1 near T_N, and the mode at ~383 cm-1 displays a slight flattening within a narrow temperature interval around the transition before continuing its anharmonic trend. Though the FWHM of the peaks is not increasing monotonically with temperature. All observed variations remain within fitting and instrumental uncertainty and therefore do not constitute a clear or systematic deviation at the transition.

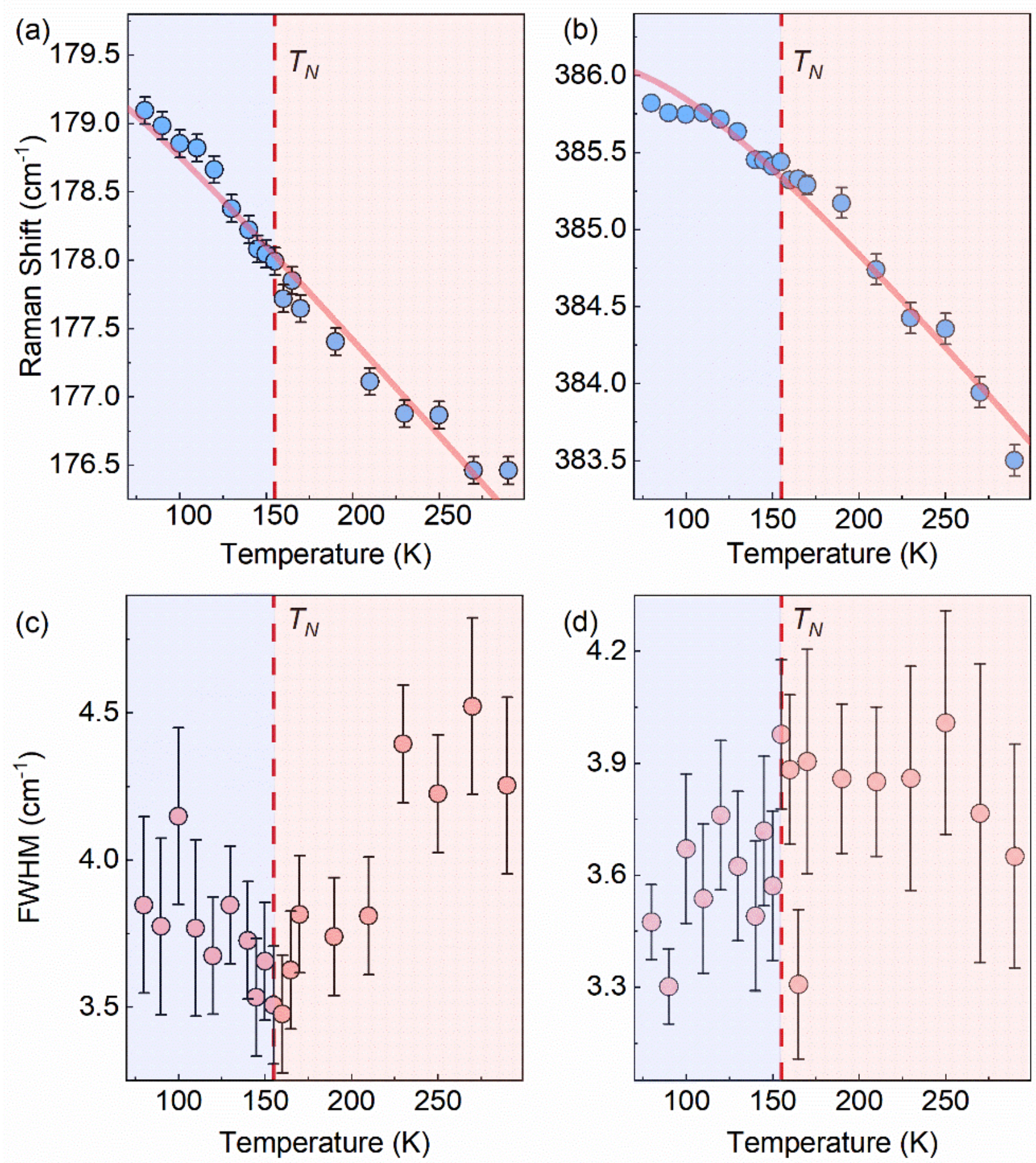

Supplementary Fig. 3: Temperature-dependent phonon modes and FWHM from 80 K to 290 K (a-b) phonon modes at 179 cm-1 and 386 cm-1 showing anharmonic trend. The blue circles represent the experimentally measured Raman peaks at different temperatures, and the solid lines show the fittings using the theoretical anharmonic model. The vertical dashed line marks the Néel temperature, T_N. No significant deviation from the anharmonic trend is observed across T_N. (c-d) Evolution of the FWHM of the associated peaks in the same temperature range. No significant deviation is observed. The error bars represent the uncertainty in the fitted peaks.

Supplementary Fig. 4 shows the polarization-resolved Raman mode at 176 cm^{-1} collected in parallel and cross polarization in the temperature range of 80 K to 270 K. No splitting is observed for this phonon mode between the two polarizations.

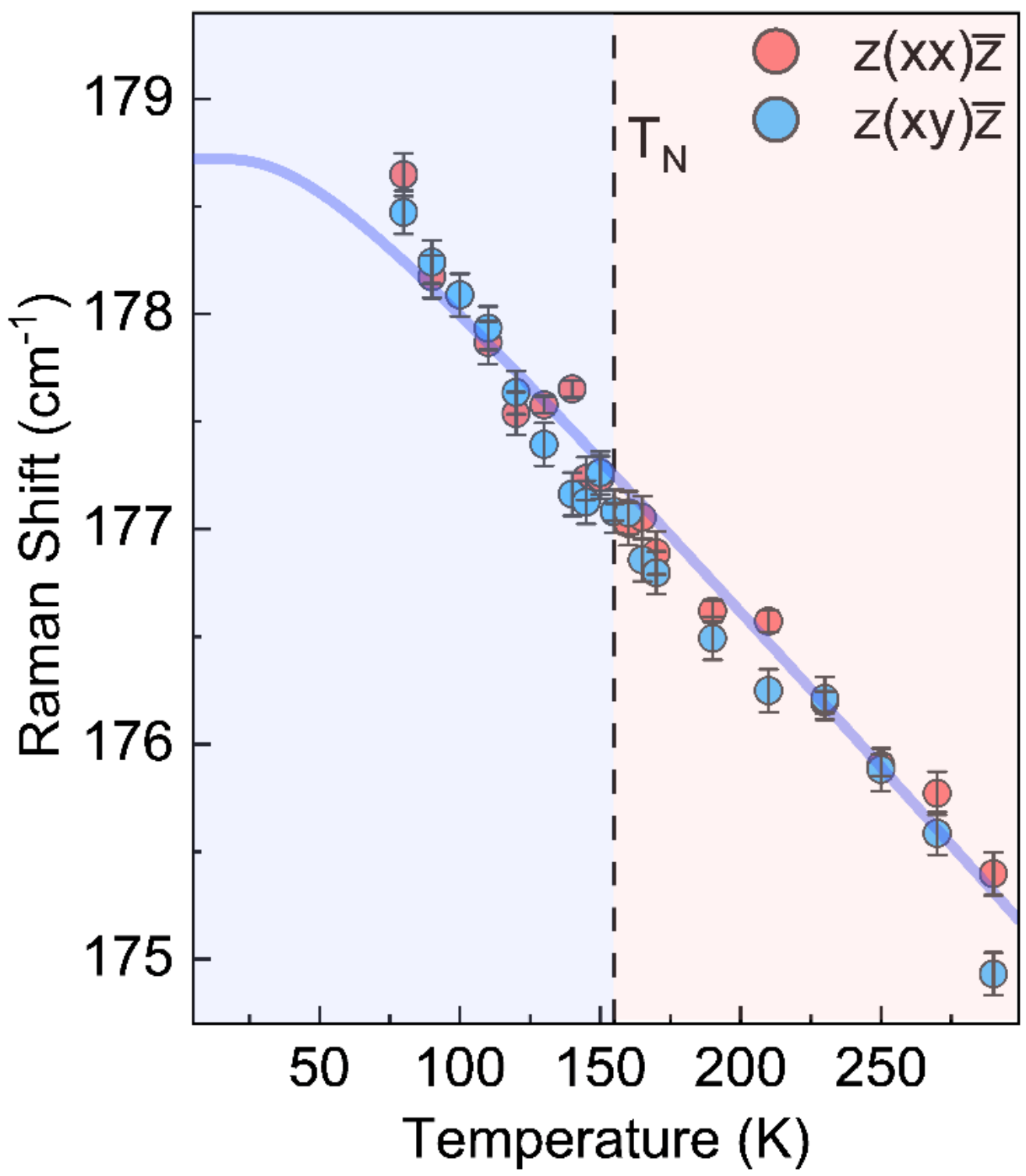

Supplementary Fig. 4: Temperature-dependent phonon mode at 176 cm^{-1} collected in parallel ($z(xx)\bar{z}$) and cross ($z(xy)\bar{z}$) polarization from the temperature 80 K to 270 K. Solid lines show the fittings using the theoretical anharmonic model. The vertical dashed line marks the Néel temperature, T_N. No significant deviation from the anharmonic trend is observed across T_N and mode splitting is not observed between the different polarization configurations. The error bars represent the uncertainty in the fitted peaks.

4. Angle-resolved Raman and BLS measurements on the exfoliated film

In our BLS experiments and scattering geometry, the surface phonon vector, **q**, lies within the (001) plane and perpendicular to the edge. Thus, the edge is the interception of the (001) plane with either (200), $(1\bar{3}0)$, and (130) planes. A plane (hkl) has normal $G_{hkl} = ha^* + kb^* + lc^*$, so the edge direction can be simply calculated as $d = G_{(001)} \times G_{(hkl)}$. Following this equation, one would obtain that the edges are either in the [010] (b-axis in the real space), [310], or $[3\bar{1}0]$ directions. we performed detailed angle-resolved Raman measurements on the exfoliated samples,

rotating the sample in a way the polarization of the incident light is parallel to one of these edges. $NiPS_3$ belongs to the C2/m space group with b-axis being the unique axis. the A_g modes are described by:

$$R(A_g) = \begin{pmatrix} a & 0 & d \\ 0 & b & 0 \\ d & 0 & c \end{pmatrix}, \qquad .R(AB_g) = \begin{pmatrix} 0 & e & 0 \\ e & 0 & f \\ 0 & f & 0 \end{pmatrix}$$

The Raman intensity is given by $I \propto |e_s.R.e_i|^2$, where e_i and e_s are the polarization vectors of the incident and scattered light, respectively. In the Raman spectra of $NiPS_3$, many of the peaks are degenerate A_g, B_g modes. The two well-defined peaks at 381 cm^{-1} and 588 cm^{-1} are purely A_g modes. The polar intensity Raman signatures of these modes are shown in Supplementary Fig. 5:

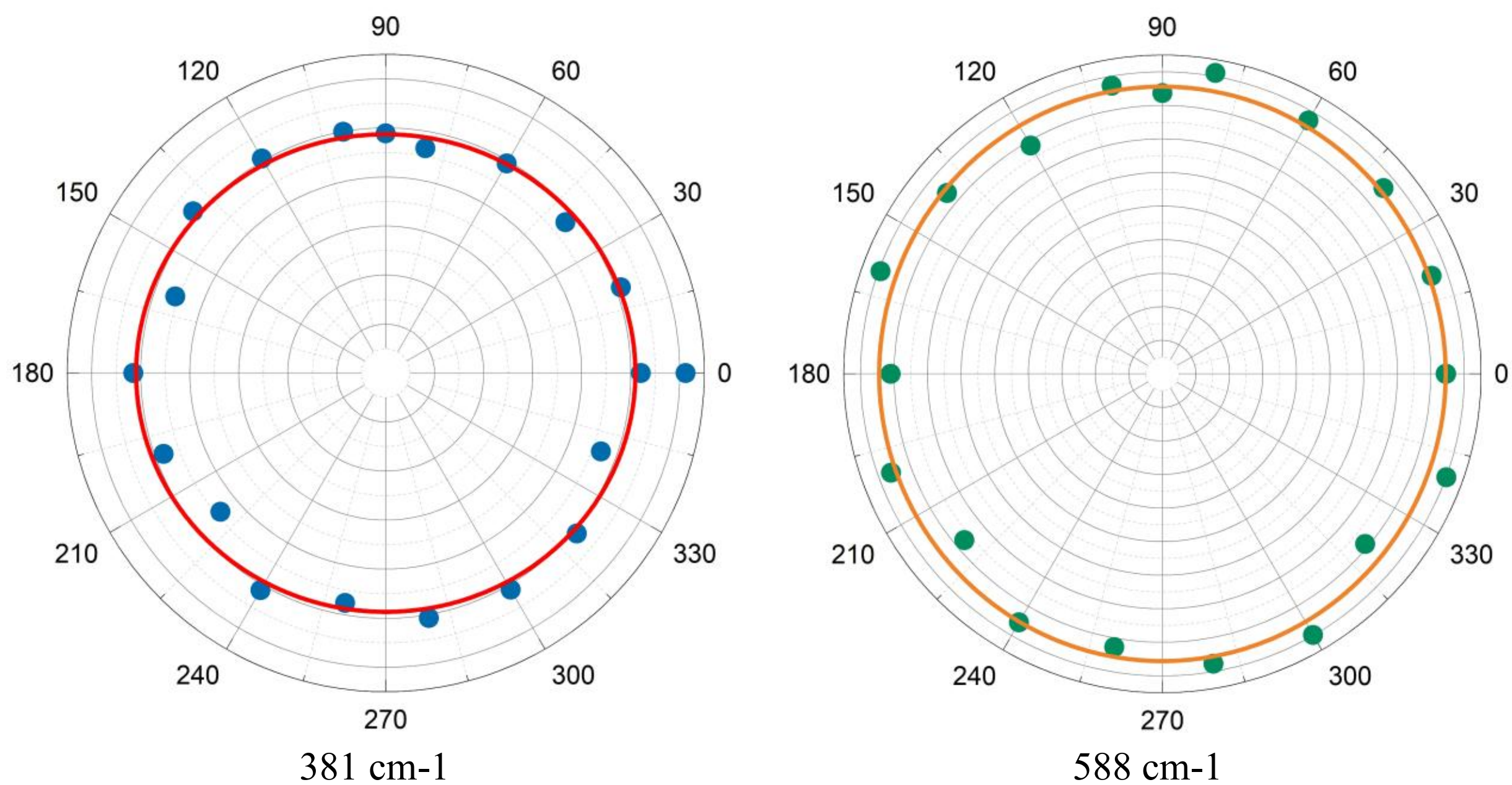

Supplementary Fig. 5: Polar plots of the Raman intensity for the A_g modes at 381 cm^{-1} (left) and 588 cm^{-1} (right), measured in the parallel polarization configuration. The experimental data are shown as symbols, while the solid red and orange lines represent fitting to the expected angular dependence, confirming the fully symmetric A_g character of both modes.

In the backscattering geometry from the basal (001) surface, the measurement is primarily sensitive to the in-plane components of the tensor, i.e. R_{aa} and R_{bb}. The calculated angular dependence shows that, if these components were significantly different, the A_g intensity would

exhibit pronounced modulation in parallel polarization and a finite signal in cross polarization upon rotating the crystal relative to the laboratory frame. Experimentally, however, the A_g modes display nearly angle-independent intensity in parallel polarization and are strongly suppressed in cross polarization for all rotation angles (Fig. 1 of the main text where Raman signatures have been obtained in random in-plane rotation). This behavior indicates that the in-plane Raman tensor elements are nearly equal, $R_{aa} = R_{bb}$, resulting in an effectively isotropic Raman response within the basal plane. Consequently, the in-plane anisotropy of the A_g modes is weak, and polarization-resolved Raman measurements alone cannot reliably distinguish the crystallographic a and b axes or assign the observed flake edges to specific the planes.

The B_g modes are associated with the off-diagonal Raman tensor element R_{ab}, which in principle enables identification of crystallographic orientation through polarization-dependent measurements. However, in $NiPS_3$, each B_g mode is spectrally degenerated with an A_g mode (Fig. 1 of the main text), so the measured Raman intensity at a given frequency always contains contributions from both symmetries (see the Supplementary Fig. 6 for the intensity map of all B_g modes). Consequently, although the B_g tensor would ideally produce angular-dependent intensity with zeros in specific polarization configurations, these features are obscured by the simultaneous presence of the B_g contribution. As a result, the polarization dependence of the measured peaks does not reach the expected zeros and cannot be used reliably to distinguish the crystallographic a and *b* axes or to assign the flake edges to the (200), (130), and $(1\bar{3}0)$ facets.

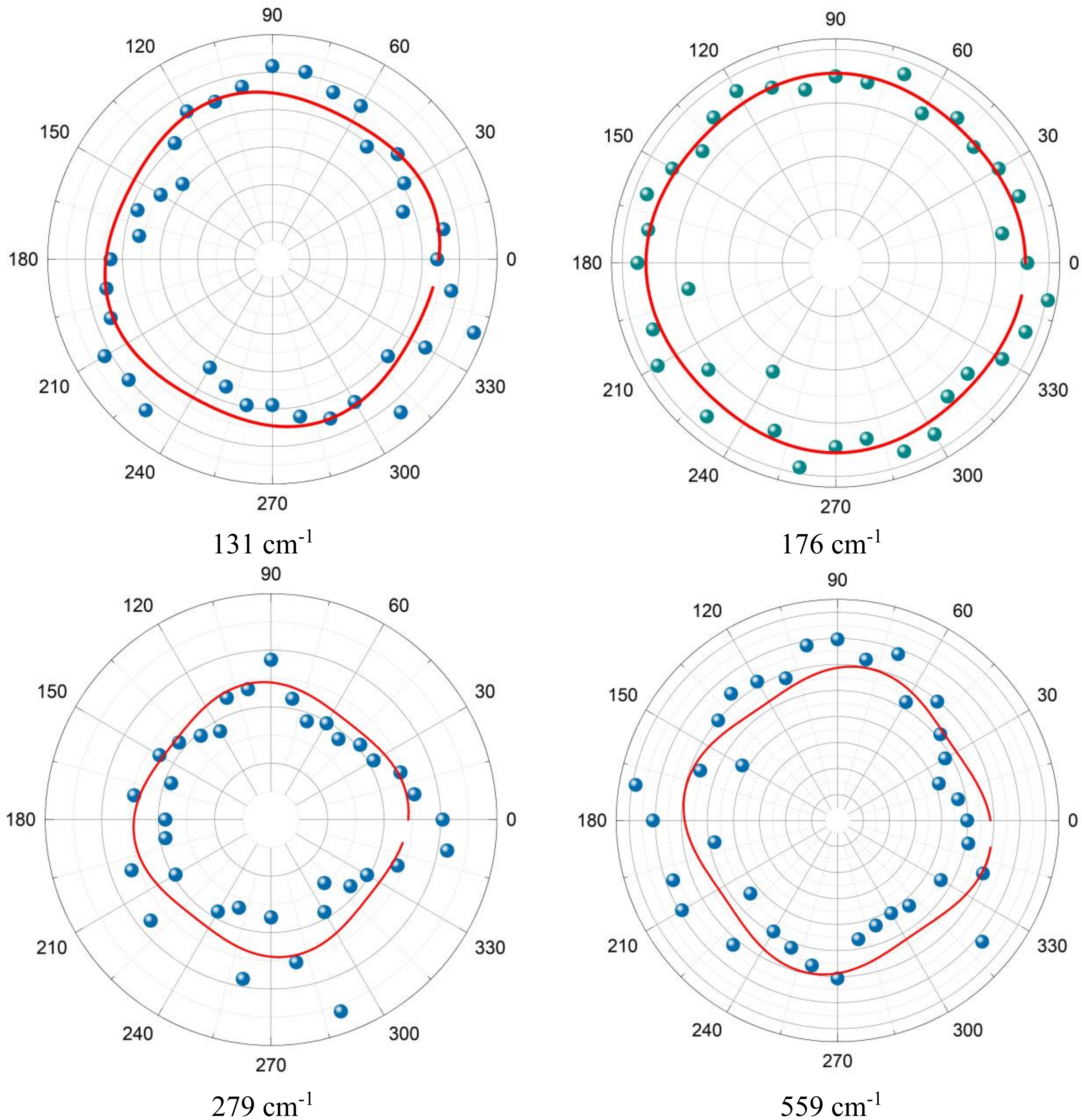

Supplementary Fig. 6: Polar plots of the Raman intensity for the B_g modes at 131, 176, 279, and 559 cm^{-1}, measured in the cross-polarization configuration. The experimental data are shown as symbols, while the solid red lines represent fits to the expected angular dependence.

However, the Raman response of the A_g modes provide an important insight: the anisotropy of the phonon modes within the *ab* plane is weak. As discussed above, the nearly angle-independent intensity in parallel polarization and the suppression of the cross-polarized signal indicate that the in-plane Raman tensor elements satisfy $R_{aa} \approx R_{bb}$, leading to an effectively isotropic response

within the basal plane. This observation suggests that the phonon properties probed in our measurements exhibit only weak directional dependence in the *ab* plane. In addition to Raman measurements, we performed BLS experiments in which the phonon wavevector, **q**, was oriented perpendicular to one of the three cleaved crystal edges in each measurement (Supplementary Fig. 7(a)). The results show no significant variation in the phonon phase velocity along these directions (Supplementary Fig. 7(b)), further confirming the weak anisotropy in the *ab* plane.

Consequently, for surface phonon measurements, the exact in-plane orientation of the phonon wavevector **q** is of limited importance, and reliable information can be obtained without precise identification of its crystallographic direction.

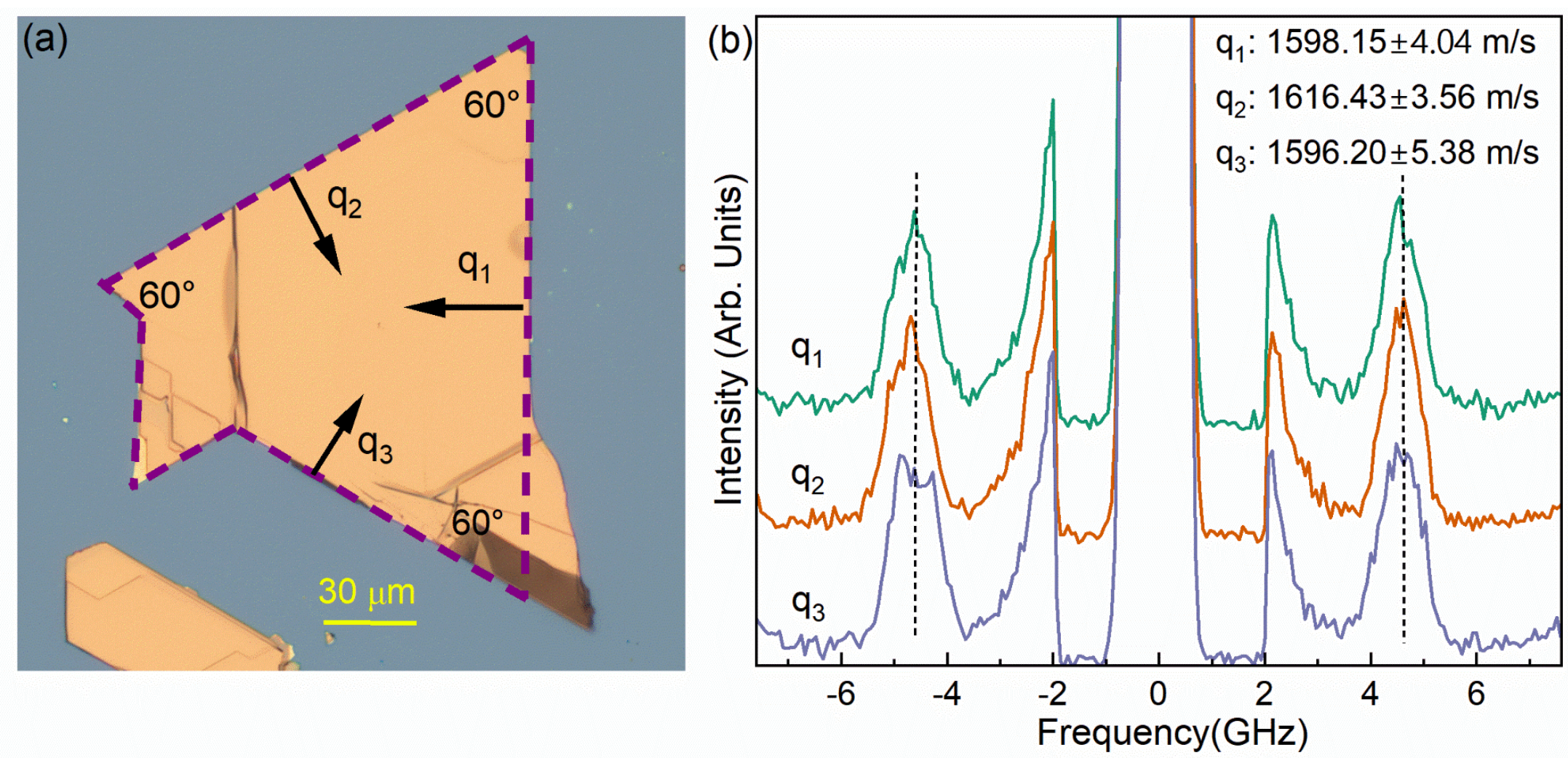

Supplementary Fig. 7: (a) Optical image of an exfoliated $NiPS_3$ flake showing three representative measurement directions ($\mathbf{q}_1$, $\mathbf{q}_2$, and $\mathbf{q}_3$), each oriented perpendicular to different crystal edges separated by ~60°. (b) Corresponding BLS spectra for the three directions, with Rayleigh wave peaks highlighted (dashed lines). The extracted phase velocities are 1598.15 ± 4.04 m/s, 1616.43 ± 3.56 m/s, and 1596.20 ± 5.38 m/s along the $\mathbf{q}_1$, $\mathbf{q}_2$, and $\mathbf{q}_3$, respectively.

5. Brillouin–Mandelstam spectroscopy of bare SiO_2/Si substrate

Supplementary Fig. 8 shows the Brillouin–Mandelstam light scattering (BMS) spectra of surface acoustic phonon from a 300 nm-thick SiO_2 layer deposited on a Si substrate, measured at different incident angles. The observed peaks correspond to the Rayleigh wave (RW), Sezawa wave (SW), and the Lamb wave (LW), consistent with the previous report[1]. These results confirm that the

phonon mode observed at ~4 GHz originates from the $NiPS_3$ layer, and not from the underlying SiO_2/Si substrate.

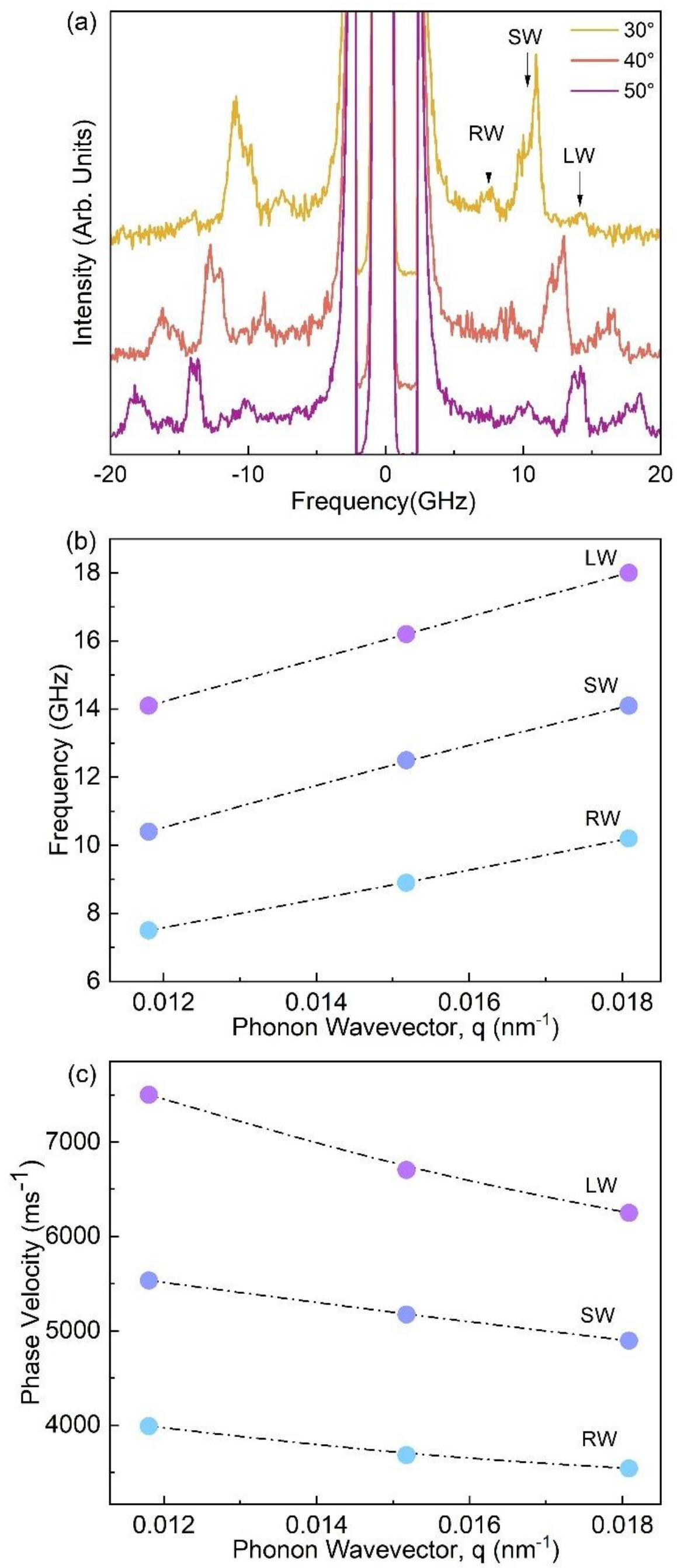

Supplementary Fig. 8: (a) Brillouin spectrum of 300 nm SiO_2 film on Si substrate performed at room temperature at different incidence angles from 30° to 50°. The peaks are associated with the Surface Rayleigh wave (RW), Sezawa wave (SW), and Lamb wave (LW). (b) Spectral position of the surface peaks identified by fitting individual Lorentzian functions and (c) the calculated phase velocity as a function of phonon wavevector, **q**.

6. Surface acoustic phonon simulation of SiO_2/Si substrate

To validate the simulation setup, COMSOL simulations were first performed for the SiO_2/Si configuration. The SiO_2 thickness was set to 225 nm based on previously reported experimental BMS data for SiO_2 film on Silicon[1]. For a chosen laser wavelength of 514.5 nm and an incident angle of 67°, the simulated mode displacement profiles are depicted in Supplementary Fig. 9. The simulation model shows the Rayleigh wave (RW) at 12.1 GHz, Sezawa wave (SW) at 17.1 GHz, and first Lamb wave (LW1) at 19.5 GHz in Supplementary Fig. 9 (a)-(c), respectively. These modes are consistent with the experimentally reported results, confirming the accuracy of our simulation model.

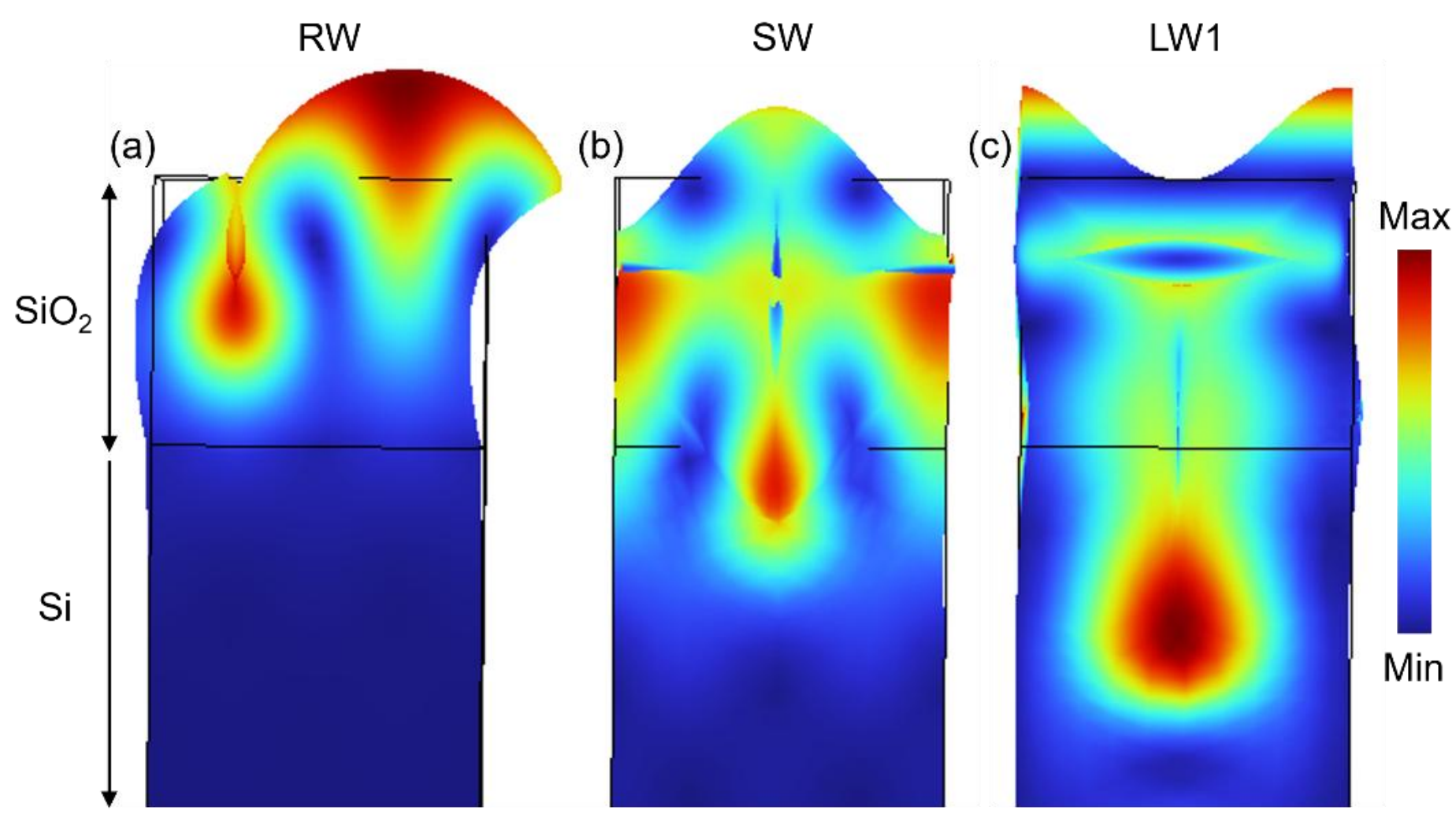

Supplementary Fig. 9: COMSOL simulation of the SiO_2/Si structure used to validate the modeling framework. Shown are the displacement profiles of surface acoustic waves: (a) Rayleigh wave (RW) at 12.1 GHz, (b) Sezawa wave (SW) at 17.1 GHz, and (c) first Lamb wave (LW_1) at 19.5 GHz. The simulated frequencies and mode shapes are in excellent agreement with reported experimental results.

7. Surface acoustic phonon simulation for $NiPS_3$ at higher q

Supplementary Fig. 10 presents the displacement profile of a 220 nm-thick $NiPS_3$ film on a SiO_2/Si substrate at $\mathbf{q}$ = 0.0222 nm^{-1}. The Rayleigh mode displacement reveals that, at higher $\mathbf{q}$, the mode penetrates less into the substrate compared to the displacement profile at $\mathbf{q}$ = 0.0135 nm^{-1} shown

in the main text. This trend is consistent with the expected behavior of Rayleigh waves, which become increasingly confined to the $NiPS_3$ layer as the in-plane wavevector increases.

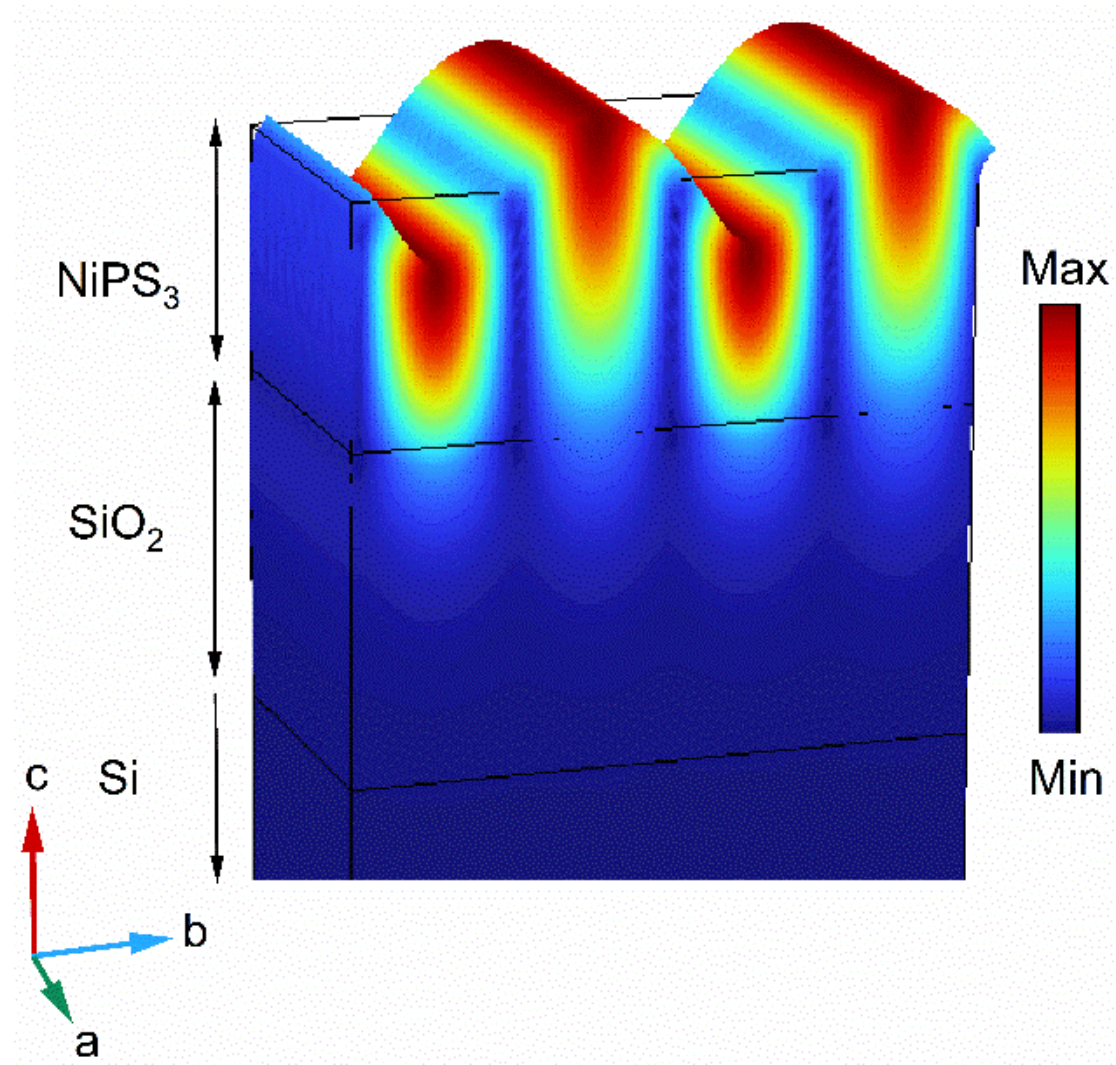

Supplementary Fig. 10: Rayleigh mode displacement profile of 220 nm-thick $NiPS_3$ film on a SiO_2/Si substrate at $\mathbf{q} = 0.0222\ nm^{-1}$.

8. Phonon dispersion for $NiPS_3$

Supplementary Fig. 11 and 12 show the phonon dispersion for antiferromagnetic (AFM) and non-magnetic (NM) phase configuration of $NiPS_3$, respectively. The calculations show no imaginary frequencies (no negative modes), confirming the dynamic stability of the structure. The band structure is calculated along the high-symmetry path Γ-C-Y2-Γ-M2-D-A-Γ-L2-Y4 in the Brillouin zone.

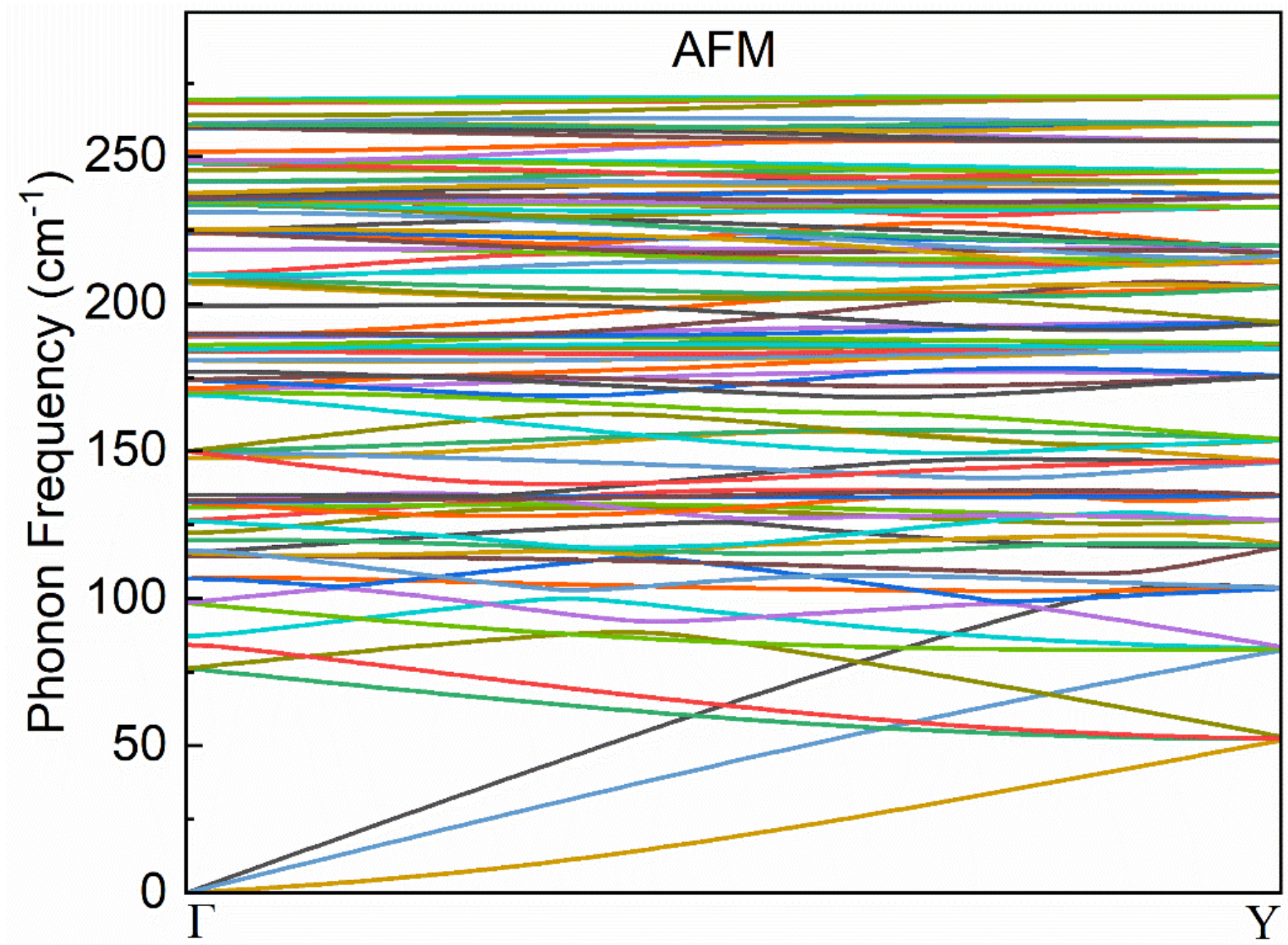

Supplementary Fig. 11: Phonon dispersion for $NiPS_3$ in the antiferromagnetic (AFM) configuration.

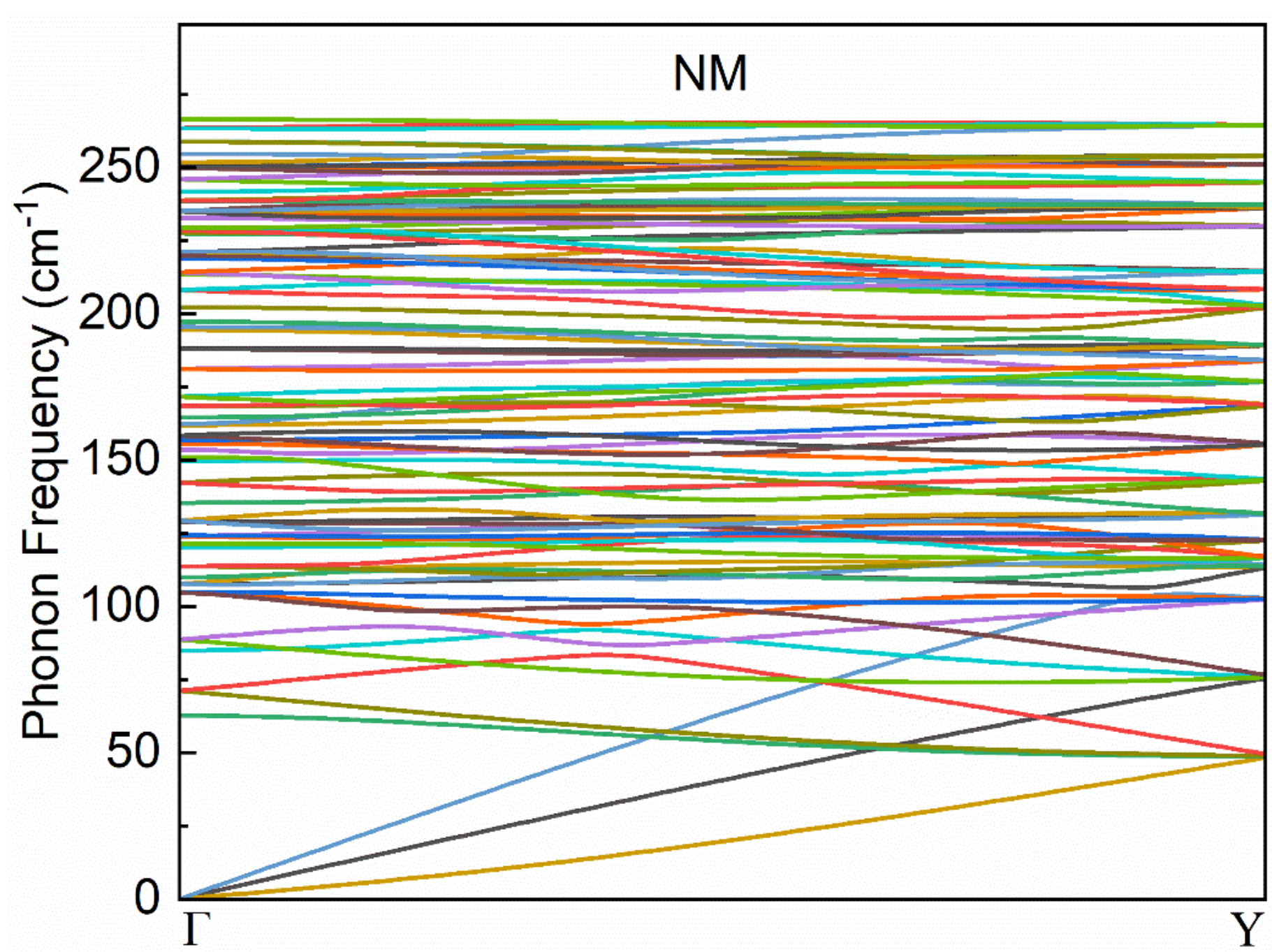

Supplementary Fig. 12: Phonon dispersion for $NiPS_3$ in the non-magnetic (NM) configuration.

REFRENCES

1. Bortolani, V., Nizzoli, F. , & Santoro, G. Strong interference effects in surface Brillouin scattering from a supported transparent film. *Phys. Rev. B* **25**, (1982).
