# Supplementary material for: Anomalous spin–lattice coupling in a 2D antiferromagnetic semiconductor revealed by surface acoustic Rayleigh waves
Source: Nat Commun. 2026 Oct 12;17:10741. doi: 10.1038/s41467-026-77234-4 (PMC13661896; doi:10.1038/s41467-026-77234-4)
Supplement: Supplementary file 2 [file 41467_2026_77234_MOESM3_ESM.pdf]

nature portfolio

Peer Review File

Anomalous spin–lattice coupling in a 2D antiferromagnetic semiconductor revealed by surface acoustic Rayleigh waves

Corresponding Author: Professor Fariborz Kargar

Parts of this Peer Review File have been redacted as indicated to remove third-party material.

This file contains all reviewer reports in order by version, followed by all author rebuttals in order by version.

Version 0:

Reviewer comments:

Reviewer #1

(Remarks to the Author)
The manuscript by Alexander A. Balandin et al., entitled Anomalous spin–lattice coupling in a 2D antiferromagnetic semiconductor revealed by surface acoustic Rayleigh waves, investigates spin-lattice coupling in 2D antiferromagnetic NiPS3 via long-wavelength acoustic phonons, with acoustic phonon softening linked to antiferromagnetic ordering and supported by theoretical calculations. The work sheds light on spin-mediated elastic property modulation, relevant to phononics and spintronics research. However, the manuscript in its current form fails to meet the publication criteria of Nature Communications, mainly due to inadequate research systematicity to support the conclusion of spin-lattice coupling in 2D antiferromagnets.
The key concerns to be thoroughly addressed are as follows:
1. The study is restricted to quasi-2D NiPS3 films (~220 nm) with no monolayer/few-layer measurements performed, thus lacking clarification of the dimensional dependence of elastic softening for strictly 2D systems, which is a core characteristic of 2D materials.
2. The optical microscopy image in Fig. S1 shows that three directions differing by 60° (or 120°) all exhibit distinct long edges, which is consistent with the threefold symmetry of the monolayer NiPS3 crystal structure. However, it cannot be determined from the figure which one of the three directions is significantly different from the other two, and how to identify which direction corresponds to the a-axis. In addition, how to determine whether the regular edges on the exfoliated sample are along the zig-zag rather than the armchair direction?
3. As noted in Fig. S4: "No significant deviation from the anharmonic trend is observed across TN, and mode splitting is not detected among different polarization configurations." This contradicts previous reports (Nat. Commun. 2019, 10, 345); please elaborate on this discrepancy.
4. Fig. 3a reveals a pronounced discrepancy between the experimental data and fitting results, particularly for the 30° and 50° datasets, where the noise amplitude is comparable to the signal intensity. Furthermore, a fitted curve is still yielded at the 50° azimuthal angle, at which no distinct sample-related peak is discernible, thereby raising critical concerns regarding the potential for excessively large fitting errors associated with this procedure.
5. The temperature points selected in Fig. 4 are inappropriate with an excessively large temperature step size, and insufficient data points are available near the phase transition point. This fails to provide adequate support for the conclusion that the phonon frequency reduction is associated with the antiferromagnetic order. In addition, the temperature-dependent variation trends of the phonon frequency and phase velocity are completely different, which precludes a direct validation of the aforementioned conclusion. The authors are therefore requested to supplement more experimental data around the phase transition temperature and conduct a more comprehensive analysis to substantiate this conclusion.
6. In Fig. 4a, the authors only present data acquired at a fixed azimuthal angle of $\theta = 35°$. Why was this specific angle selected? Is it feasible to perform the BMS measurements along the a- and b-axes of the crystal? Given the strong in-plane spin anisotropy of NiPS3, supplementing additional experimental data at different azimuthal angles would likely reveal the anisotropic characteristics of the acoustic phonon mode, which is critical for a comprehensive understanding of the system.

Reviewer #2

(Remarks to the Author)

Kargar, F., Balandin, A. A. et al report a detailed study of spin-lattice coupling in NiPS3 using Raman and Brillouin-Mandelstam scattering spectroscopies, which trigger renormalization of elastic tensor and thus a change in macroscopic mechanical properties of the antiferromagnet.

I find this work of excellent quality, which presents very valuable findings in spin-lattice coupling mechanisms in MPS3s, and which will help research communities related to 2D materials and magnetism to better understand experiments in spectroscopy, phase transition dynamics, magnetostriction and nanomechanics of MPS3 and other antiferromagnets. I believe the quality of experimental data and its interpretation, supported by DFT and analytical modeling, will be well received and appreciated by the broad readership of Nature Communications. I recommend publishing this work with a minor rework, which is mostly related to discussions and interpretations of the findings with respect to the literature.

1. As authors mention in the main text, in NiPS3 near ~180 cm-1 the phonon frequency difference of two intertwined modes is known to exhibit a rather sharp feature (splitting) at TN, resembling an antiferromagnetic order parameter-like contribution. This splitting is often observed for MPS3 family [Nat Commun 10, 345 (2019), Phys Rev B 103, 235411 (2021)] and in case of NiPS3 is likely originating in a change in monoclinic stacking of the layers, i.e. there is a certain small change in stacking/interlayer shear that is associated with the TN [Nat Commun 10, 345 (2019), Nat Commun 13, 6598 (2022)]. This spin-shear coupled phenomenon is also often found in case for other materials of the same family and similar spin configuration [Nat Commun 13, 6598 (2022), Nature 620, 988–993 (2023)].

The fact that authors do not observe this splitting is rather unexpected and quite worrying. Thus, more in-depth discussion should be provided that explains the possible reasons behind this absence, as one naively may think it may even be related to sample geometry that suppresses interlayer shear due to clamping (substrate-supported flakes vs bulk vs free-standing membranes, etc)

2. Although findings presented in Fig4 are rather trustful, I find it confusing in how Fig4 is presented. In particular, guide-to-an-eye dashed lines in Fig.4a and b suggest that the transition is continuous (expected from the second order nature of it). Normally this indeed would be the case for an antiferromagnetic-to-paramagnetic transition. However, there is an ongoing discussion in the community of whether these transitions in MPS3s (incl. NiPS3), which involve lattice deformations, can be considered purely of second-order (continuous) [Nat Commun 13, 6598 (2022), Nature 620, 988–993 (2023), Phys. Rev. B 107, L220407 (2023)] or have a first-order (discontinuous) component to it [J. Phys.: Condens. Matter 37 055801 (2025), Phys. Rev. 126, 104 (1962), Hyperfine Interact 16, 625–628 (1983)].

Thus, in case authors have any arguments towards whether this transition is continuous or discontinuous, it would be beneficial to make it clear in the manuscript or comment on that more explicitly.

3. According to DFT calculations performed by authors, the largest variation in elastic constants is associated with c66 - the in-plane shear component, which also goes in-line with c11, c12 and c22 change. This is probably due to some small in-plane spontaneous magnetostriction in NiPS3 which was studied previously both experimentally and by DFT [Nat Commun 14, 8503 (2023)]. This effect does deform a lattice in-plane but, although measurable, is usually considered rather poorly pronounced, especially in comparison to CoPS3 or FePS3. The most interesting observation here I believe streams from the sensitivity analysis of elastic constants performed by the authors, which shows that the largest sensitivity to the Rayleigh-wave velocity is from coming from c33 and c44. This suggests a sizable shear- and out-of-plane elastic coefficient softening, which indeed may lead to the reduction in phase velocity, as it is connected to rich and pronounced shear-mode dynamics in NiPS3 - a significant mechanism that is known to couple spins to lattice in this material and even to produce a substantial critical slowdown in relaxation dynamics of both mechanical and magnetic parameters [Nat Commun 13, 6598 (2022)].

I kingly suggest authors to discuss further on these arguments and possibly expand the analysis further on whether these phenomena, i.e. spin-shear deformation and elastic softening (and thus reduction of phase velocity), are “two sides of the same coin”.

4. In the second-to-last sentence of the discussion section authors state that "NiPS3 exhibits an exceptionally large spin-phonon coupling constant" which "places NiPS3 among the strongest spin-phonon-coupled materials", surpassing even FePS3, CrBr3, or CGT by an order of magnitude. I believe this statement cannot be correct: lattice constants of NiPS3 exhibit only minor anomalies near TN, while its in-plane thermal expansion coefficient show a more-or-less normal behavior as a function of temperature, somewhat resembling the trend set by the temperature dependence of its specific heat [Nat Commun 11, 2698 (2020)]. In contrast, both CrBr3 and CGT exhibit extraordinally large anomalies in their thermal expansion coefficients, even turning it negative below Tc [npj Quantum Mater. 6, 19 (2021), Phys. Rev. Research 4, L022040 (2020)]. Also, even if we only consider optical evidence, in case of FePS3 almost the whole low-frequency part of the Raman spectrum is nenormalised below TN, which suggests extremely high spin-phonon coupling [Nano Lett. 16, 12, 7433–7438 (2016), Phys. Rev. Lett. 127, 097401 (2021)]. All this suggests that spins are coupled much weaker to lattice (and thus phonons) in NiPS3 than in the named compounds. Authors may kindly consider to either clarify/rephrase or remove this statement.

Version 1:

Reviewer comments:

Reviewer #1

(Remarks to the Author)
The authors have addressed most of my concerns in a substantive manner. In particular, they added measurements on an additional $NiPS_3$ flake with a different thickness, provided denser temperature-dependent BMS data near the Néel temperature, and clarified the scattering geometry and the meaning of the incidence angle θ. These additions significantly improve the systematicity and reproducibility of the experimental evidence supporting Rayleigh-wave softening across the magnetic transition.
The authors have also supplemented the crystallographic and directional analyses using TEM, angle-resolved Raman, and BMS measurements along different edge-related directions. The additional TEM measurements reassign the sample edges from the a-axis to the b-axis. Although the revised results still do not identify which of the three edge-related directions corresponds to the b-axis, the authors have removed the previous statement implying direct identification of the crystallographic axis. Given that this remaining ambiguity appears to have limited impact on the main BMS results, I consider this issue acceptable. The added analyses and more cautious wording improve the rigor of the paper.
One remaining point concerns the comparison between the two temperature-dependent datasets. The additional measurements on the 260 nm flake are appreciated and clearly expand the dataset. However, the detailed temperature evolution of the anomaly does not appear entirely identical in the two samples. In the 220 nm sample, the frequency initially follows the expected lattice-stiffening trend upon cooling from room temperature to approximately 200 K, followed by a distinct drop near TN and remaining nearly constant at lower temperatures. In contrast, the 260 nm sample exhibits a much sharper and more localized anomaly around TN, rather than a gradual evolution over a broader temperature range. While the manuscript treats the two datasets as demonstrating the same qualitative behavior, the apparent differences in the evolution and character of the anomaly deserve some discussion. Since both the sample thickness and phonon wavevector differ between the two measurements, it remains unclear whether these distinctions arise from thickness effects, wavevector dependence, sample variation, or different manifestations of the underlying magnetoelastic response.

Reviewer #2

(Remarks to the Author)
I believe the authors have significantly improved the manuscript following this round of revisions. Their responses to my comments are clear, thorough, and convincing, and they have adequately addressed all of my concerns. I have no further objections and recommend this work for publication.

Open Access This Peer Review File is licensed under a Creative Commons Attribution 4.0 International License, which permits use, sharing, adaptation, distribution and reproduction in any medium or format, as long as you give appropriate credit to the original author(s) and the source, provide a link to the Creative Commons license, and indicate if changes were made.
In cases where reviewers are anonymous, credit should be given to 'Anonymous Referee' and the source.
The images or other third party material in this Peer Review File are included in the article’s Creative Commons license, unless indicated otherwise in a credit line to the material. If material is not included in the article’s Creative Commons license and your intended use is not permitted by statutory regulation or exceeds the permitted use, you will need to obtain

permission directly from the copyright holder.
To view a copy of this license, visit https://creativecommons.org/licenses/by/4.0/

REVIEWER COMMENTS

Reviewer #1 (Remarks to the Author):

The manuscript by Alexander A. Balandin et al., entitled Anomalous spin–lattice coupling in a 2D antiferromagnetic semiconductor revealed by surface acoustic Rayleigh waves, investigates spin-lattice coupling in 2D antiferromagnetic NiPS3 via long-wavelength acoustic phonons, with acoustic phonon softening linked to antiferromagnetic ordering and supported by theoretical calculations. The work sheds light on spin-mediated elastic property modulation, relevant to phononics and spintronics research. However, the manuscript in its current form fails to meet the publication criteria of Nature Communications, mainly due to inadequate research systematicity to support the conclusion of spin-lattice coupling in 2D antiferromagnets.

The key concerns to be thoroughly addressed are as follows:

1. The study is restricted to quasi-2D NiPS3 films (~220 nm) with no monolayer/few-layer measurements performed, thus lacking clarification of the dimensional dependence of elastic softening for strictly 2D systems, which is a core characteristic of 2D materials.

Response: We thank the reviewer for this comment regarding the dimensional dependence of the magnetoelastic response. We agree that investigating the evolution of elastic softening from bulk to few-layer and monolayer limits would provide valuable insight into the role of dimensionality in 2D magnetic systems. However, such a systematic study presents significant experimental challenges in the context of Brillouin–Mandelstam spectroscopy (BMS).

For exfoliated samples with thickness below the wavelength of light, scattering of light by the ripple mechanism becomes dominant, leading to efficient detection of surface acoustic waves. In this regime, the BMS spectra are primarily governed by scattering from surface acoustic modes[1]. The characteristics of these modes are influenced not only by the elastic properties of the flake itself, but also by the underlying substrate. In the limit of single- and bilayer samples, the BMS signal from the material is overwhelmed by scattering originating from the substrate surface. As a result, meaningful measurements are effectively restricted to samples with thicknesses on the order of ~100 nm or greater. One may consider performing measurements on suspended samples to mitigate substrate contributions. However, this approach introduces additional challenges. Unlike Raman spectroscopy, which is typically conducted in a backscattering geometry with the optical axis normal to the sample surface, BMS requires rotation of the sample to access and select the probed phonon wavevector (please refer to Figure R9 in this response letter.) This experimental requirement makes measurements on suspended exfoliated samples – especially mono- and bilayer samples with lateral dimensions less than 5 µm – impractical.

In addition, temperature-dependent measurements require relatively long acquisition times for each spectrum, and the reduced signal-to-noise ratio in ultrathin samples further increases the required measurement time. These factors make systematic temperature-dependent BMS

measurements on monolayer or few-layer $NiPS_3$ currently very challenging. Indeed, the present study represents one of the few recent investigations able to access ultra-low-energy phonon spectra in exfoliated layered materials, enabled by the state-of-the-art instrumentation developed by the team.

The ~220 nm and 260 nm films studied here provide a practical compromise, enabling reliable detection of Rayleigh surface acoustic modes while still probing a quasi-two-dimensional system with layered character. Importantly, the observed magnetoelastic softening reflects intrinsic spin–lattice coupling within the layers, which is expected to persist in thinner samples, although its magnitude and detailed behavior may evolve with thickness. Note that we performed additional measurements on flakes with ~260 nm thickness per reviewers' suggestions and presented the new results in the revised manuscript. The new results are combined with previous results shown as Figure 4 in the revised manuscript.

2. The optical microscopy image in Fig. S1 shows that three directions differing by 60° (or 120°) all exhibit distinct long edges, which is consistent with the threefold symmetry of the monolayer NiPS3 crystal structure. However, it cannot be determined from the figure which one of the three directions is significantly different from the other two, and how to identify which direction corresponds to the a-axis. In addition, how to determine whether the regular edges on the exfoliated sample are along the zig-zag rather than the armchair direction?

Response: We thank the reviewer for this comment. Indeed, optical microscopy alone cannot uniquely determine which of the three equivalent edge directions corresponds to a specific crystallographic axis. To address this point, we have performed high-resolution transmission electron microscopy (HRTEM) measurements, which are now presented as Figure 3 in the revised manuscript. The optical images were acquired on mechanically exfoliated flakes on Si/SiO_2 substrates. However, HRTEM cannot be reliably performed on such substrate-supported samples. Therefore, we prepared separate samples via liquid-phase exfoliation for TEM characterization to determine the crystallographic orientation of the edges, specifically to identify the relevant slip planes and directions (i.e., the planes and in-plane directions along which the edges are aligned).

The TEM analysis reveals lattice fringes with spacings of ~0.28–0.29 nm and relative angles of ~60°, consistent with the flake geometry observed under optical microscopy. Based on these observations, edges forming 60° (or 120°) angles can be assigned to the (200), $(1\bar{3}0)$, and (130) planes, in agreement with prior TEM studies of $NiPS_3$ [2].

The following figure has been added to the revised manuscript per the reviewer's suggestion:

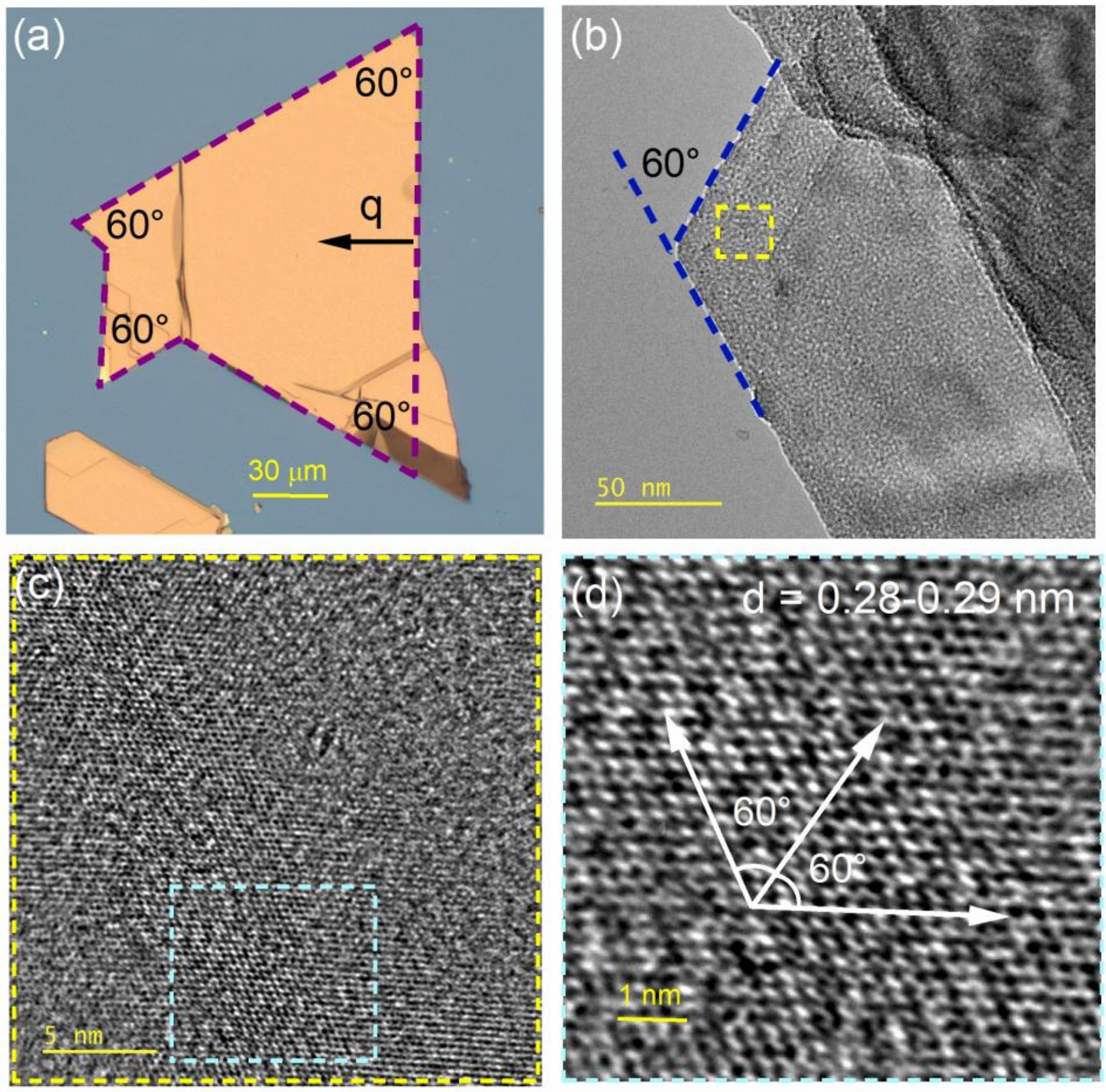

[**Fig. R1: morphology and crystallinity of $NiPS_3$.** (a) Optical image of an exfoliated $NiPS_3$ flake on a SiO_2/Si substrate. The lateral dimensions exceed 100 μm × 100 μm, and characteristic crystal cleavage angles of ~ 60° are indicated. (b) TEM image of the liquid phase exfoliated flake on the TEM grid, confirming the ~ 60° angular relationship. The region highlighted by the yellow dashed square is shown in (c), while the blue dashed region corresponds to (d). (c) High-resolution TEM image of the selected area. (d) Magnified lattice-resolved image with arrows indicating the (200), $(1\bar{3}0)$, and (130) crystallographic planes, forming ~ 60° angles consistent with the crystal symmetry.**]**

In our experiments and scattering geometry, the surface phonon vector, q, lies within the (001) plane and perpendicular to the edge. Thus, the edge is the interception of the (001) plane with either (200), $(1\bar{3}0)$, and (130) planes. A plane (hkl) has normal $G_{hkl} = ha^* + kb^* + lc^*$, so the edge direction can be simply calculated as $d = G_{(001)} \times G_{(hkl)}$. Following this equation, one would obtain that the edges are either in the [010] (b-axis in the real space), [310], or $[3\bar{1}0]$ directions. we performed detailed angle-resolved Raman measurements on the exfoliated samples, rotating the sample in a way the polarization of the incident light is parallel to one of these edges. $NiPS_3$ belongs to the C2/m space group with b-axis being the unique axis. The A_g and B_g modes are described by:

$$R(A_g) = \begin{pmatrix} a & 0 & d \\ 0 & b & 0 \\ d & 0 & c \end{pmatrix}, \qquad .R(B_g) = \begin{pmatrix} 0 & e & 0 \\ e & 0 & f \\ 0 & f & 0 \end{pmatrix}$$

The Raman intensity is given by $I \propto |\mathbf{e_s}.R.\mathbf{e_i}|^2$, where $\mathbf{e_i}$ and $\mathbf{e_s}$ are the polarization vectors of the incident and scattered light, respectively. In the Raman spectra of $NiPS_3$, many of the peaks are degenerate A_g, B_g modes. The two well-defined peaks at 381 cm^{-1} and 588 cm^{-1} are purely A_g modes. The polar intensity Raman signatures of these modes are shown in Fig. R2:

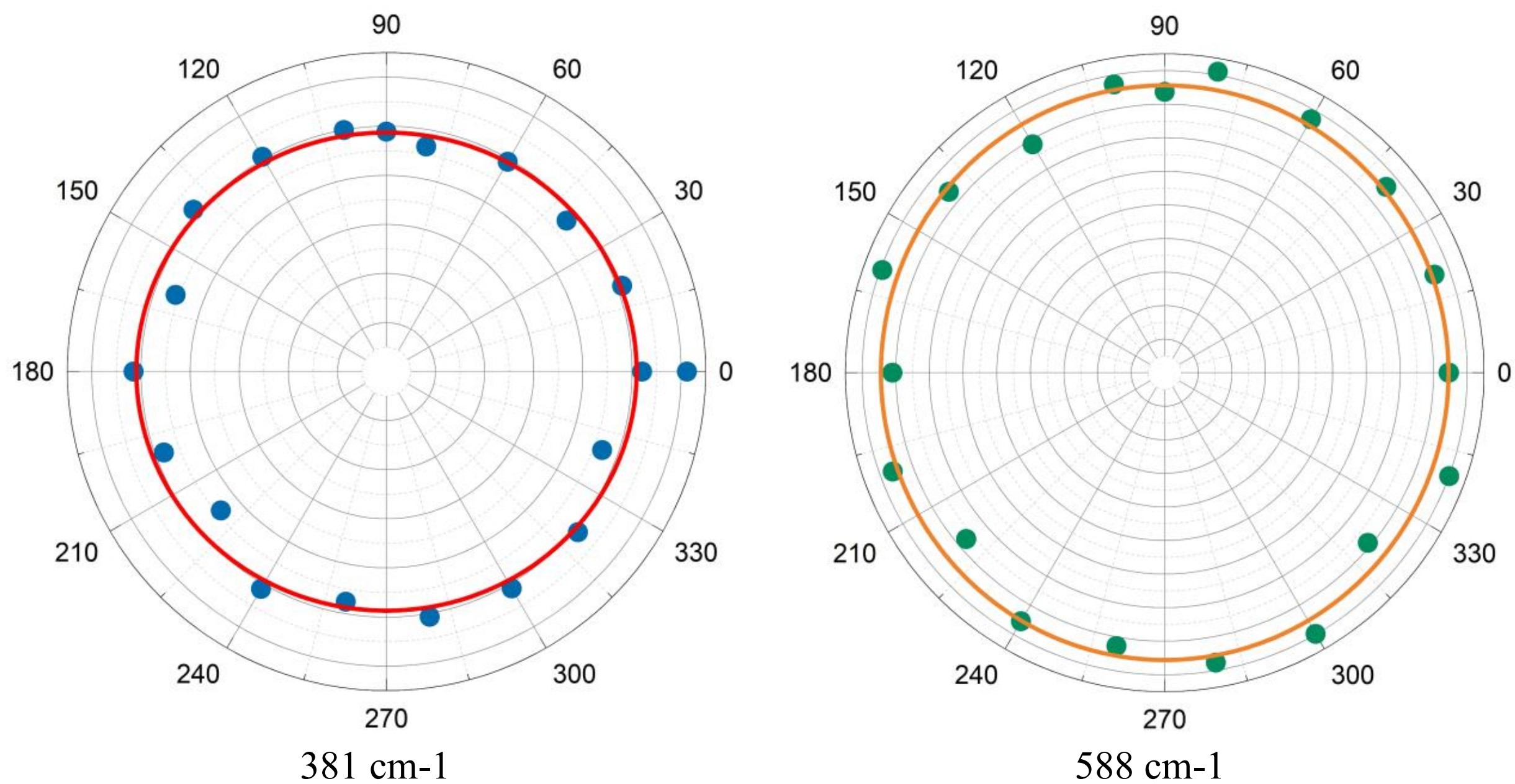

[Fig. R2: Polar plots of the Raman intensity for the A_g modes at 381 cm^{-1} (left) and 588 cm^{-1} (right), measured in the parallel polarization configuration. The experimental data are shown as symbols, while the solid red and orange lines represent fitting to the expected angular dependence, confirming the fully symmetric A_g character of both modes.]

In the backscattering geometry from the basal (001) surface, the measurement is primarily sensitive to the in-plane components of the tensor, i.e. R_{aa} and R_{bb}. The calculated angular dependence shows that, if these components were significantly different, the A_g intensity would exhibit pronounced modulation in parallel polarization and a finite signal in cross polarization upon rotating the crystal relative to the laboratory frame. Experimentally, however, the A_g modes display nearly angle-independent intensity in parallel polarization and are strongly suppressed in cross polarization for all rotation angles (Fig. 1 of the main text where Raman signatures have been obtained in random in-plane rotation). This behavior indicates that the in-plane Raman tensor elements are nearly equal, $R_{aa} = R_{bb}$, resulting in an effectively isotropic Raman response within the basal plane. Consequently, the in-plane anisotropy of the A_g modes is weak, and polarization-resolved Raman measurements alone cannot reliably distinguish the crystallographic a and b axes or assign the observed flake edges to specific the planes.

The B_g modes are associated with the off-diagonal Raman tensor element R_{ab}, which in principle enables identification of crystallographic orientation through polarization-dependent measurements. However, in $NiPS_3$, each B_g mode is spectrally degenerated with an A_g mode (Fig. 1 of the main text), so the measured Raman intensity at a given frequency always contains contributions from both symmetries (see the Fig. R3 for the intensity map of all B_g modes). Consequently, although the B_g tensor would ideally produce angular-dependent intensity with zeros in specific polarization configurations, these features are obscured by the simultaneous presence of the A_g contribution. As a result, the polarization dependence of the measured peaks does not reach the expected zeros and cannot be used reliably to distinguish the crystallographic a and b axes or to assign the flake edges to the (200), (130), and $(1\bar{3}0)$ facets.

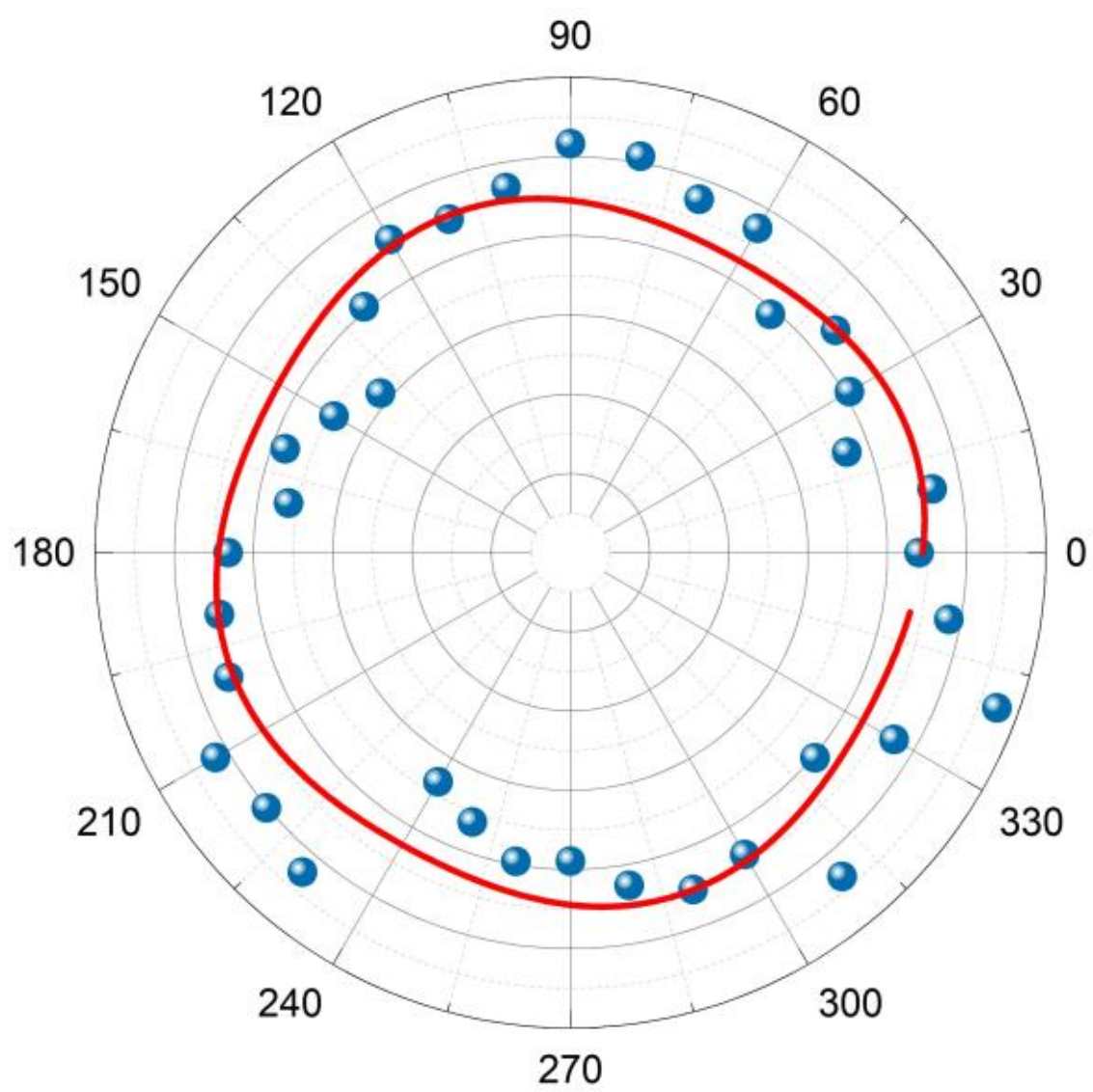

131 cm^{-1}

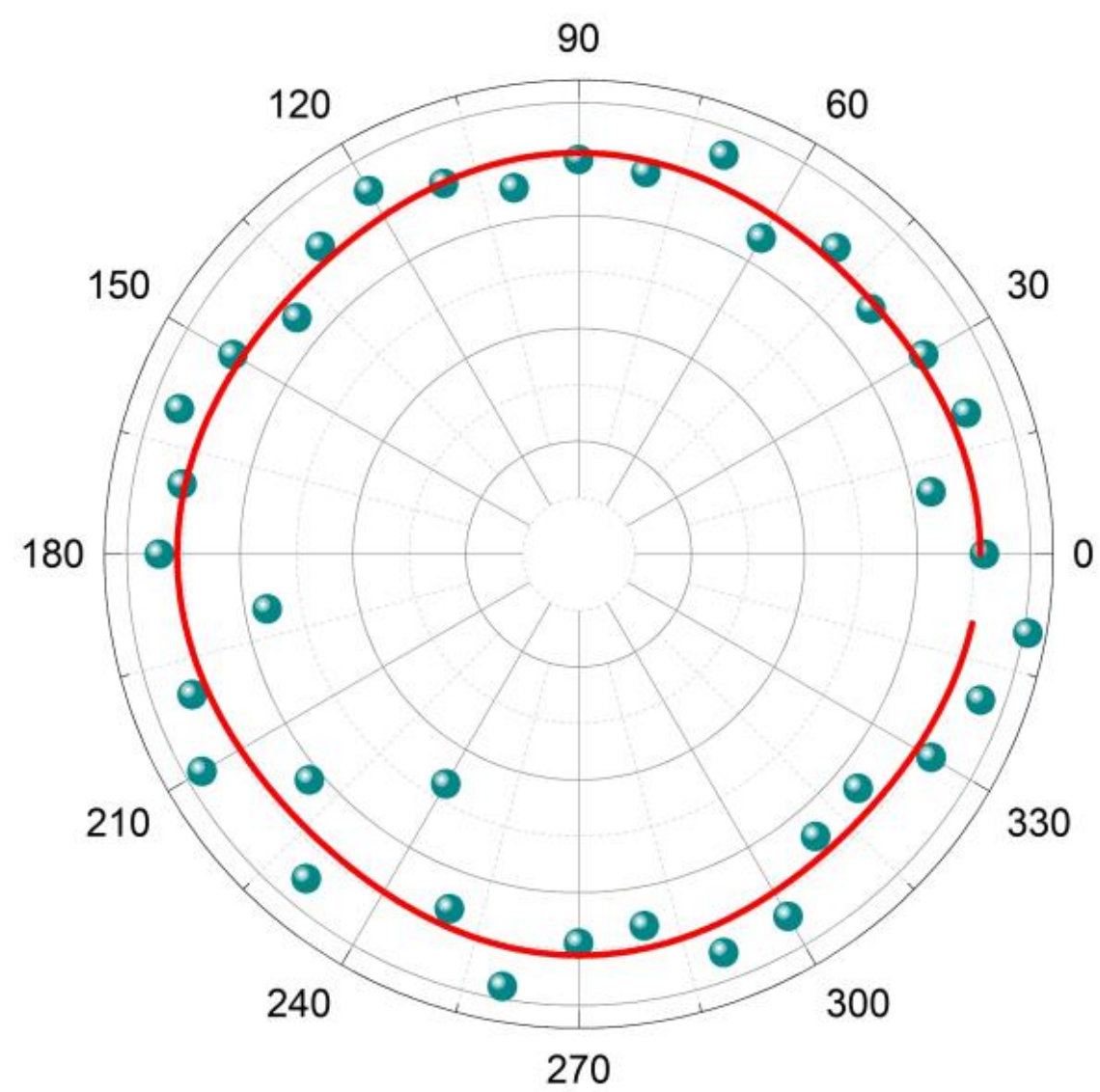

176 cm^{-1}

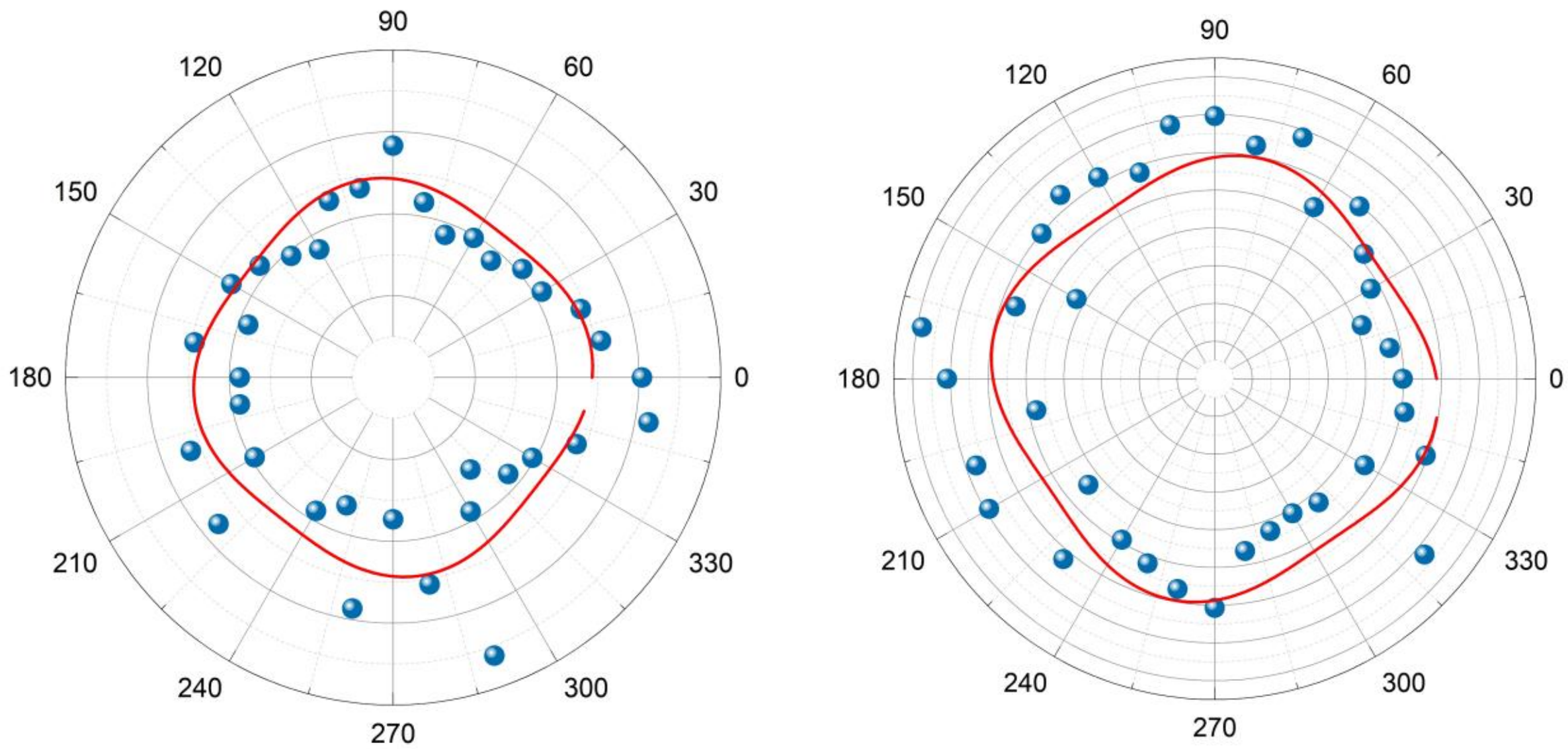

279 cm^{-1} 559 cm^{-1}

[Fig. R3: Polar plots of the Raman intensity for the B_g modes at 131, 176, 279, and 559 cm^{-1}, measured in the cross-polarization configuration. The experimental data are shown as symbols, while the solid red lines represent fits to the expected angular dependence.]

However, the Raman response of the A_g modes provide an important insight: the anisotropy of the phonon modes within the a–b plane is weak. As discussed above, the nearly angle-independent intensity in parallel polarization and the suppression of the cross-polarized signal indicate that the in-plane Raman tensor elements satisfy $R_{aa} \approx R_{bb}$, leading to an effectively isotropic response within the basal plane. This observation suggests that the phonon properties probed in our measurements exhibit only weak directional dependence in the a–b plane. Consequently, for surface phonon measurements, the exact in-plane orientation of the phonon wavevector q is of limited importance, and reliable information can be obtained without precise identification of its crystallographic direction.

To confirm the latter, we performed BLS experiments in which the phonon wavevector, *q*, was oriented perpendicular to each of the three cleaved crystal edges in each measurement (Fig. R4 (a)). The results show no significant variation in the phonon phase velocity along these directions (Fig. R4 (b)), further confirming the weak anisotropy in the *a*–*b* plane.

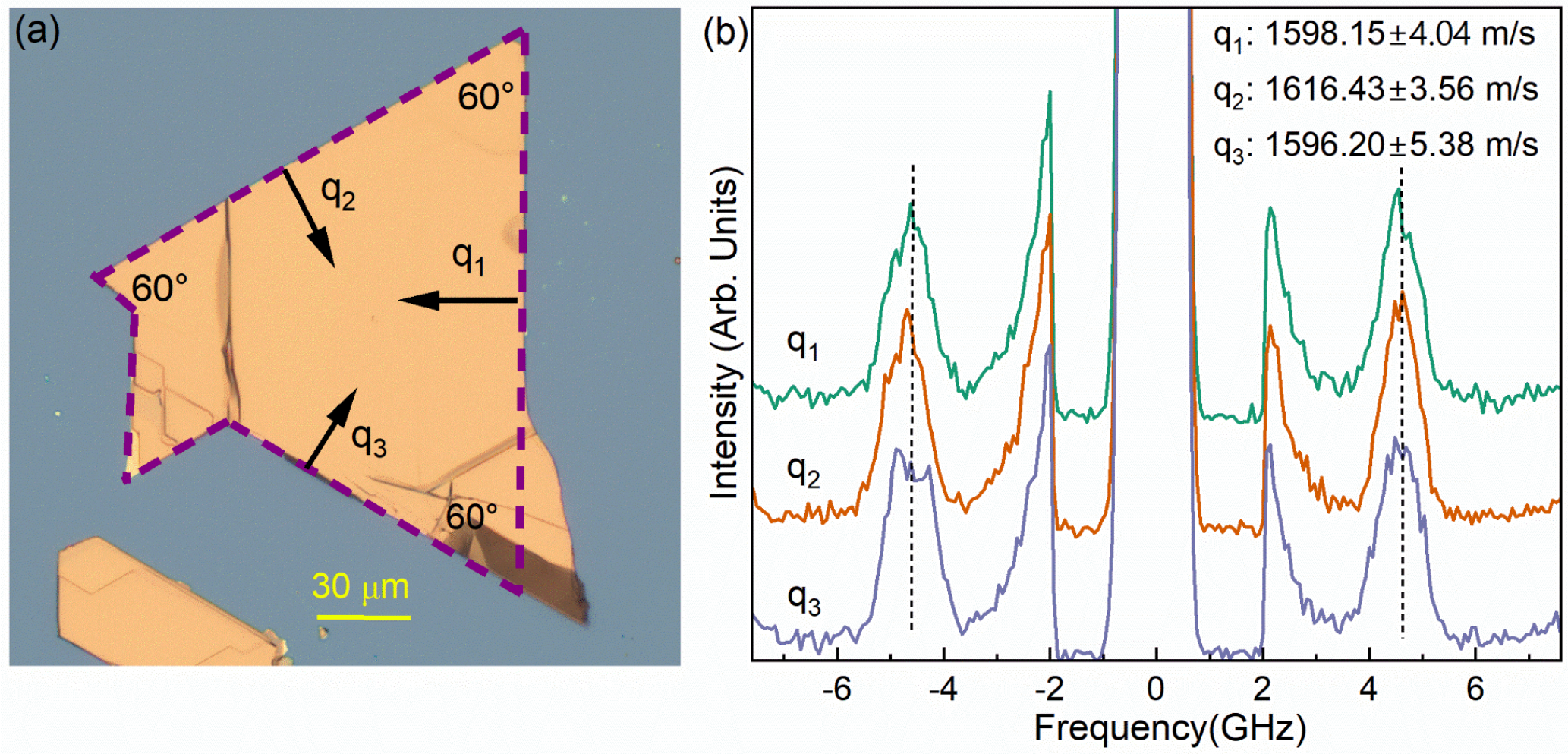

[Fig. R4: (a) Optical image of an exfoliated $NiPS_3$ flake showing three representative measurement directions (q_1, q_2, and q_3), each oriented perpendicular to different crystal edges separated by ~60°. (b) Corresponding BLS spectra for the three directions, with Rayleigh wave peaks highlighted (dashed lines). The extracted phase velocities are 1598.15 ± 4.04 m/s, 1616.43 ± 3.56 m/s, and 1596.20 ± 5.38 m/s along the q_1, q_2, and q_3, respectively.]

3. As noted in Fig. S4: "No significant deviation from the anharmonic trend is observed across TN, and mode splitting is not detected among different polarization configurations." This contradicts previous reports (Nat. Commun. 2019, 10, 345); please elaborate on this discrepancy.

Response: We thank the reviewer for raising this important point regarding the comparison with previous reports. Following the reviewer's suggestion, we performed additional, more rigorous analyses of our Raman data. In contrast to Ref. (Nat. Commun. 2019, 10, 345), we do not observe a clear splitting of the Raman mode at ~176 cm^{-1}. At room temperature, there is no splitting while the splitting below the transition is about 0.2 cm^{-1} (see Fig. R5, the solid and dashed lines correspond to the parallel and cross polarizations, respectively.)

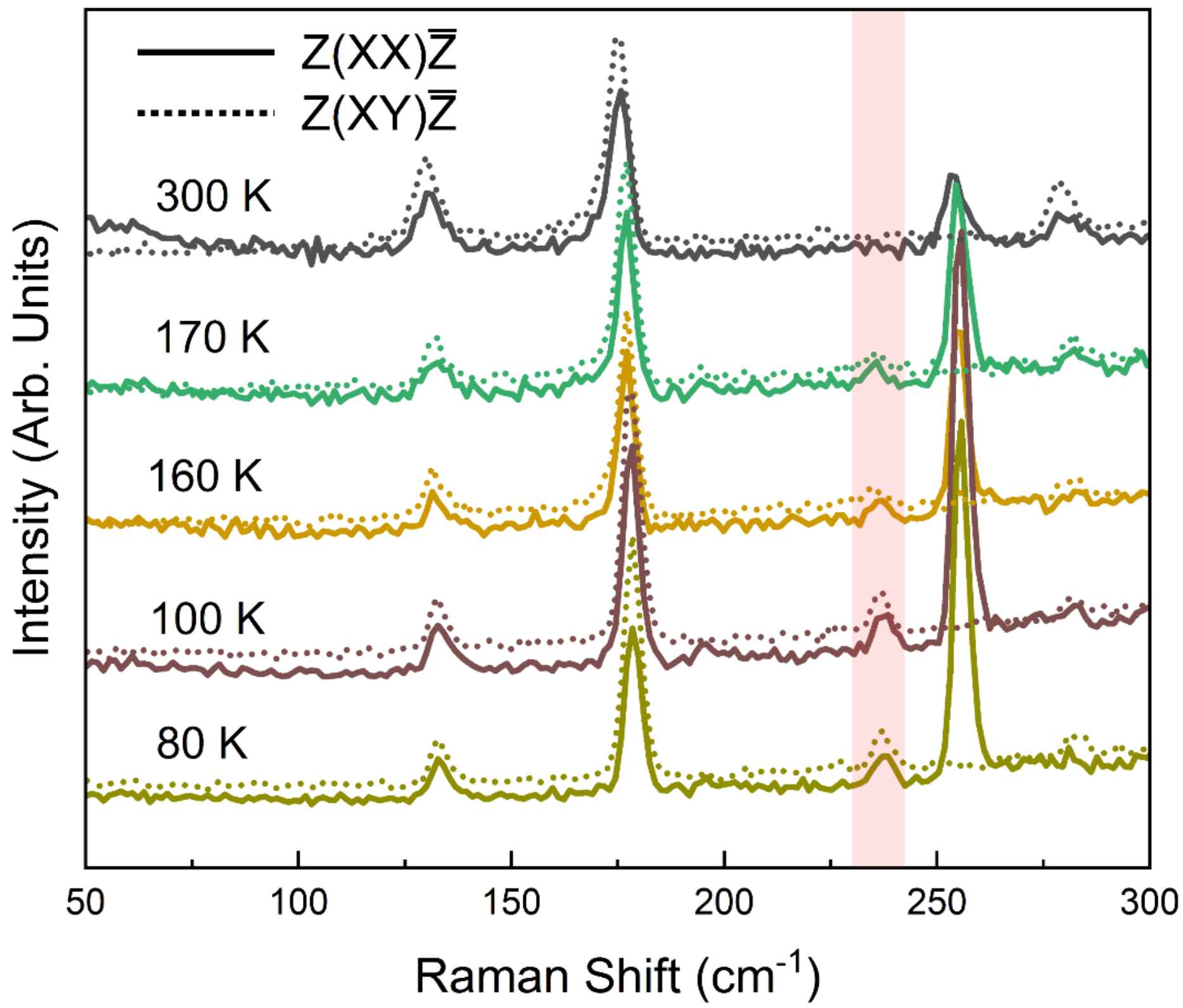

[Fig. R5: Temperature-dependent Raman spectra in the parallel (solid lines) and cross (dashed lines) polarization configurations. The Raman mode near ~176 cm^{-1} shows no observable splitting at room temperature, while only a very small splitting (~0.2 cm^{-1}) emerges below the transition temperature. This indicates that any potential mode splitting is minimal and remains close to the experimental resolution.]

We note that the spectral resolution of our Raman system is approximately ~1–2 cm^{-1}. In Ref. (Nat. Commun. 2019, 10, 345), the reported splitting is relatively small and becomes more pronounced at lower temperatures than those explored in our study (77 K to 300 K). As such, the absence of a clearly resolved splitting in our measurements may be attributed, in part, to the limited spectral resolution and temperature range. Furthermore, the visibility of such subtle splitting can depend sensitively on experimental factors, including sample thickness, residual strain, and stacking faults, all of which can influence symmetry breaking and the corresponding Raman response. Note that while our measurements do not resolve a clear mode splitting comparable to that reported previously, this does not necessarily contradict earlier findings. Rather, it suggests that the effect is subtle and may depend strongly on sample-specific and experimental conditions.

4. Fig. 3a reveals a pronounced discrepancy between the experimental data and fitting results, particularly for the 30° and 50° datasets, where the noise amplitude is comparable to the signal intensity. Furthermore, a fitted curve is still yielded at the 50° azimuthal angle, at which no distinct

sample-related peak is discernible, thereby raising critical concerns regarding the potential for excessively large fitting errors associated with this procedure.

Response: We thank the reviewer for carefully examining Fig. 3a and for raising this important point. We agree that, particularly at 50°, the signal-to-noise ratio is reduced due to the lower scattering intensity of the Rayleigh mode at these geometries. However, even in these cases, the peak position can still be reliably extracted within the fitting uncertainty of which error bars are included in Fig. 3b (see Fig. R6). To verify the robustness of the analysis, we repeated the BMS measurements on a new NiPS3 flake under the same experimental conditions. The results show consistent dispersion behavior and confirm the reproducibility of the extracted peak positions across different samples. Please see Figure R7 (panel (a), h=260 nm), where the peak at 50° is more clear. Note that the frequency, and hence the phase velocity of the Rayleigh peak, depends on the thickness of the sample as well. The small deviation of the new results from those submitted previously is due to the thickness difference of the new sample and the old one. Figure R7 (which is in the revised version represented as Figure 4) contains the new experimental data.

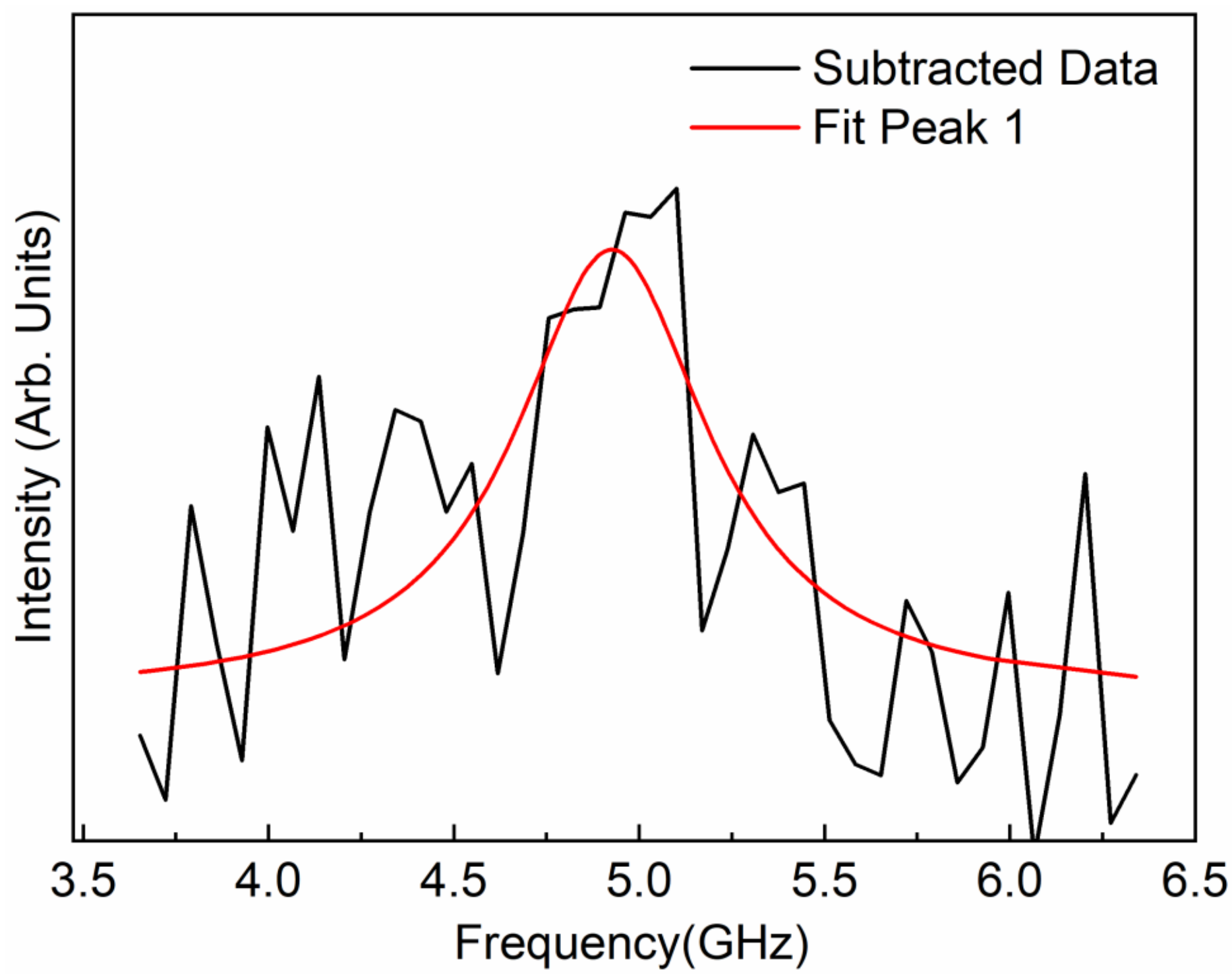

[Fig. R6: The subtracted data of BMS spectra of the exfoliated $NiPS_3$ with h = 220 nm collected at room temperature with incidence angles of 50°. The Lorentzian fitting is plotted in red curve.]

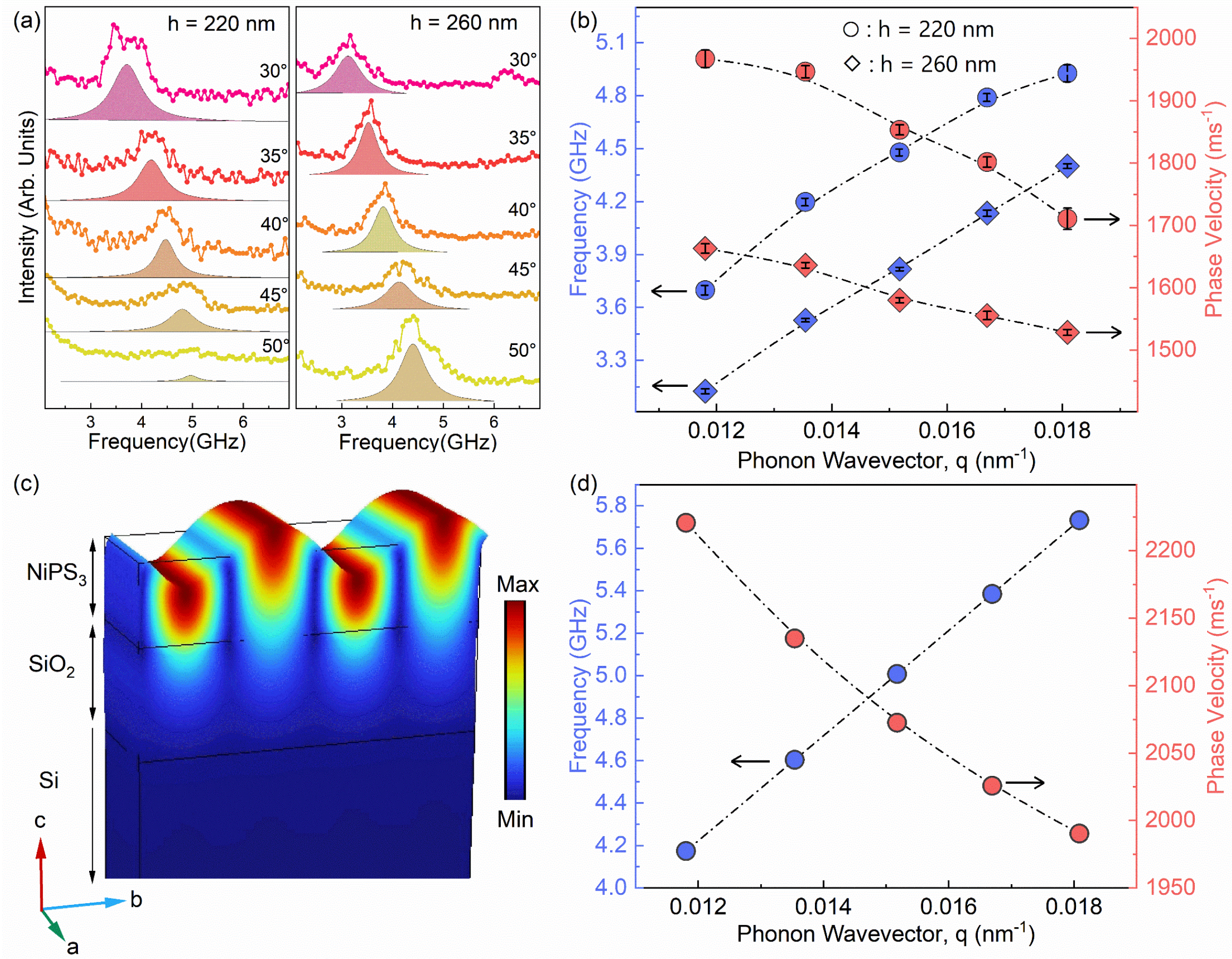

[Fig. R7: Experimental and theoretical Rayleigh-wave dispersion in $NiPS_3$. a) BMS spectra of the exfoliated $NiPS_3$ film with the thickness of ~ 220 nm and 260 nm collected at room temperature for incidence angles between 30° and 50°. A single dispersive peak is observed and assigned to the surface Rayleigh wave (RW). The shaded areas represent the individual Lorentzian fits used to extract the peak frequencies. (b) Spectral position, f, of the RW peak (blue circles) obtained by Lorentzian fitting, together with the corresponding phase velocity (red circles) as a function of wavevector, q. Circle symbols denote the 220 nm film, while diamond symbols represent the 260 nm film. The dashed line marks the trend of decreasing phase velocity with increasing q. (c) Displacement field of the RW mode calculated using the continuum elastic model at $q = 0.0135\ \mathrm{nm}^{-1}$ for the film with the thickness of 220 nm, showing surface-localized, elliptical motion predominantly within the $NiPS_3$ layer. (d) Simulated RW dispersion and phase velocity in the non-magnetic (NM) state, extracted from finite-element eigenmode analysis. The simulated trend shows good agreement with the experimental results in panel (b).]

5. The temperature points selected in Fig. 4 are inappropriate with an excessively large temperature step size, and insufficient data points are available near the phase transition point. This fails to provide adequate support for the conclusion that the phonon frequency reduction is associated with the antiferromagnetic order. In addition, the temperature-dependent variation trends of the phonon frequency and phase velocity are completely different, which precludes a direct validation of the

aforementioned conclusion. The authors are therefore requested to supplement more experimental data around the phase transition temperature and conduct a more comprehensive analysis to substantiate this conclusion.

Response: We thank the reviewer for this important comment regarding the temperature resolution and data density near the Néel temperature. To address this point, we have performed additional temperature-dependent BMS measurements on a new NiPS3 flake (the angle-dependent data for the same flake is presented as Figure 4 in the revised manuscript), with significantly dense data points. The new dataset (please see Figure R8), confirms the same qualitative behavior, namely a clear reduction of the Rayleigh-wave frequency and phase velocity at and below the Néel temperature, supporting our original conclusion regarding magnetoelastic softening associated with the onset of antiferromagnetic order. We also add the spectral position of the peaks in the revised figure.

In addition, while the original dataset was acquired at a fixed incidence angle of 35° corresponding to $q = 0.0135\ \text{nm}^{-1}$, the new measurements were performed at 40° corresponding to $q = 0.0152\ \text{nm}^{-1}$. The consistency between the two datasets demonstrates that the observed softening is robust and does not depend on the specific choice of the laser incident angle, further confirming that the effect is intrinsic to the material. The new data is presented as Figure 5 in the revised manuscript. We added the new dataset (panels c, d) as Fig.5 in the revised manuscript. Please see the attached Fig. R8 below.

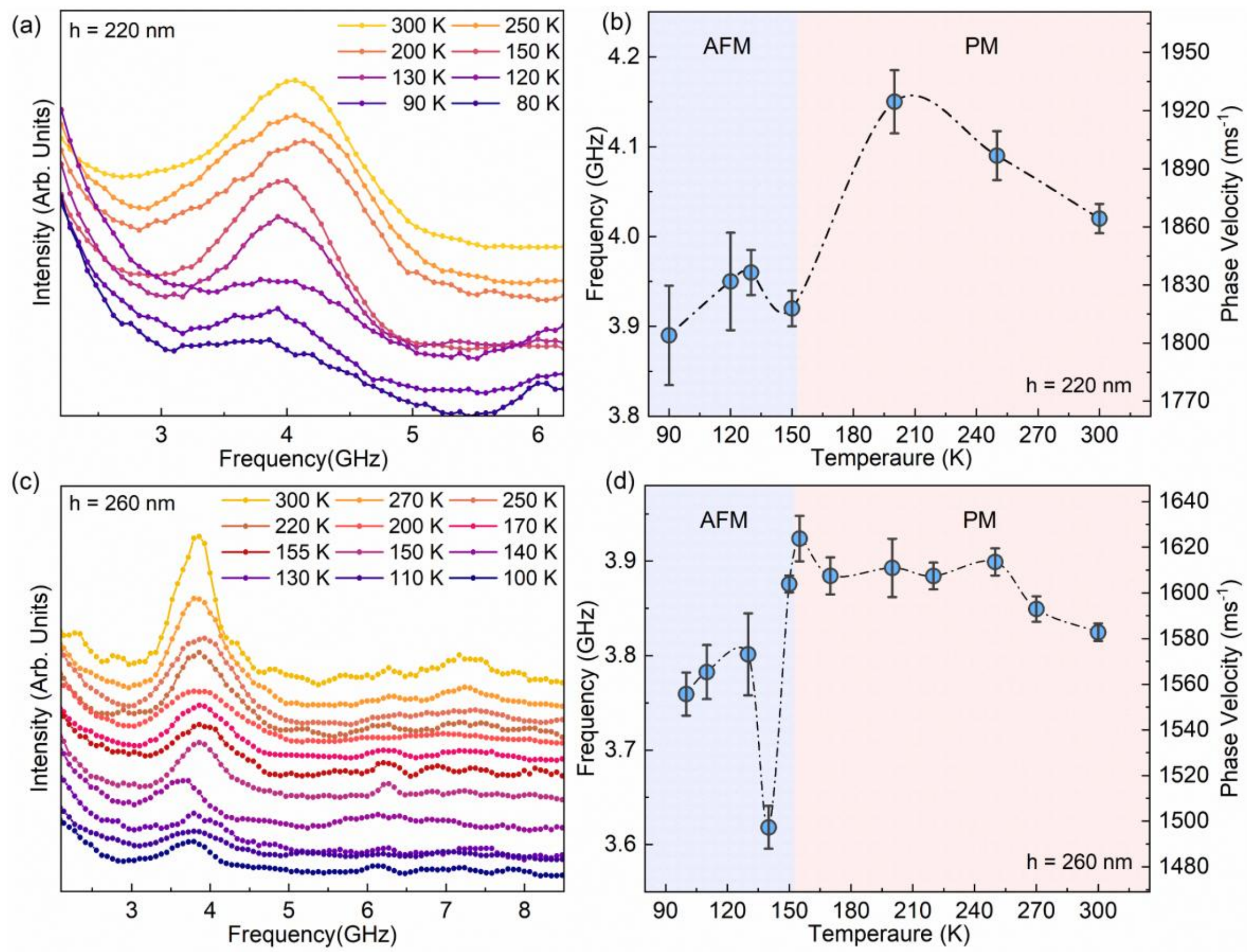

[**Fig. R8: Temperature dependence of the Rayleigh surface acoustic wave in $NiPS_3$.** (a) and (c) BMS spectra of $NiPS_3$ film exfoliated on SiO_2/Si substrate measured between 300 K and 80 K at constant wavevector $q = 0.0135\ \mathrm{nm}^{-1}$ and $q = 0.0152\ \mathrm{nm}^{-1}$ for film with a thickness of 220 nm and 260 nm, respectively. Upon cooling, the Rayleigh-wave frequency initially increases due to lattice stiffening, then exhibits a pronounced drop at 150 K near the Néel temperature (~155 K), followed by saturation at lower temperatures. (b) and (d) Phase velocity of the Rayleigh-wave as a function of temperature for film with a thickness of 220 nm and 260 nm, respectively. The velocity in the 220 nm film decreases from ~1920 ms^{-1} in the paramagnetic (PM) phase to ~1815 ms^{-1} in the antiferromagnetic AFM phase, corresponding to a ~6% reduction across the transition, while in 260 nm film it reduced by ~7.3% The shaded regions mark the PM and AFM phases. The anomaly in velocity reflects the magnetoelastic softening associated with the onset of zigzag AFM order in $NiPS_3$.]

6. In Fig. 4a, the authors only present data acquired at a fixed azimuthal angle of $\theta = 35°$. Why was this specific angle selected? Is it feasible to perform the BMS measurements along the a- and b-axes of the crystal? Given the strong in-plane spin anisotropy of NiPS3, supplementing additional experimental data at different azimuthal angles would likely reveal the anisotropic characteristics of the acoustic phonon mode, which is critical for a comprehensive understanding of the system.

Response: We believe there may be a misunderstanding regarding the definition of the incident angle. We emphasize that θ does not represent the azimuthal angle; rather, it denotes the angle between the incident laser wavevector (k_i) and the surface normal to the surface (N) (please see Figure R9). In the backscattering geometry used here, the scattered wavevector, k_s, is parallel to k_i. When probing surface acoustic waves, varying θ changes the probed phonon wavevector. For exfoliated $NiPS_3$ films with thickness (~220 nm) smaller than the laser wavelength (λ = 532 nm), the BMS spectra are dominated by surface acoustic modes. In this regime, the out-of-plane component of the phonon momentum is not uniquely defined, and only the in-plane component is conserved in the scattering process[1]. The in-plane phonon wavevector is given by $q = (4\pi/\lambda)\sin\theta$, where θ is the angle of incidence measured relative to the surface normal (please see Figure R9). Therefore, varying θ changes only the magnitude of the phonon wavevector q, not its direction. As a result, there is no qualitative difference between choosing θ = 35° or another angle; only the probed wavevector magnitude varies.

[Figure Redacted]

The measurements presented in Fig. 4a were performed at a fixed incidence angle of 35°, at which the Rayleigh mode exhibits strong scattering intensity and a well-defined peak, enabling reliable extraction of its temperature dependence. To further address this point and verify the robustness of our observations, we have performed additional temperature-dependent measurements on a new sample at a different incidence angle (40°), corresponding to a different phonon wavevector (Figure R8, panels c, d). The consistent softening behavior observed in both datasets confirms that the main conclusion – magnetoelastic renormalization of the Rayleigh mode across the magnetic transition – is not sensitive to the specific measurement geometry.

We thank Reviewer 1 for the constructive comments, which have helped improve the manuscript. We hope that we have addressed all points to the best of our experimental capabilities.

Reviewer #2 (Remarks to the Author):

Kargar, F., Balandin, A. A. et al report a detailed study of spin-lattice coupling in NiPS3 using Raman and Brillouin-Mandelstam scattering spectroscopies, which trigger renormalization of elastic tensor and thus a change in macroscopic mechanical properties of the antiferromagnet.

I find this work of excellent quality, which presents very valuable findings in spin-lattice coupling mechanisms in MPS3s, and which will help research communities related to 2D materials and magnetism to better understand experiments in spectroscopy, phase transition dynamics, magnetostriction and nanomechanics of MPS3 and other antiferromagnets. I believe the quality of experimental data and its interpretation, supported by DFT and analytical modeling, will be well received and appreciated by the broad readership of Nature Communications. I recommend publishing this work with a minor rework, which is mostly related to discussions and interpretations of the findings with respect to the literature.

Response: We thank the reviewer for finding that our manuscript presents "very valuable findings" and recommending that for publication with minor rework.

1. As authors mention in the main text, in NiPS3 near ~180 cm-1 the phonon frequency difference of two intertwined modes is known to exhibit a rather sharp feature (splitting) at TN, resembling an antiferromagnetic order parameter-like contribution. This splitting is often observed for MPS3 family [Nat Commun 10, 345 (2019), Phys Rev B 103, 235411 (2021)] and in case of NiPS3 is likely originating in a change in monoclinic stacking of the layers, i.e. there is a certain small

change in stacking/interlayer shear that is associated with the TN [Nat Commun 10, 345 (2019), Nat Commun 13, 6598 (2022)]. This spin-shear coupled phenomenon is also often found in case for other materials of the same family and similar spin configuration [Nat Commun 13, 6598 (2022), Nature 620, 988–993 (2023)]. The fact that authors do not observe this splitting is rather unexpected and quite worrying. Thus, more in-depth discussion should be provided that explains the possible reasons behind this absence, as one naively may think it may even be related to sample geometry that suppresses interlayer shear due to clamping (substrate-supported flakes vs bulk vs free-standing membranes, etc)

Response: We thank the reviewer for raising this important point regarding the comparison with previous reports. Following the reviewer's suggestion, we performed additional, more rigorous analyses of our Raman data (presented in Fig. R5). In contrast to Ref. (Nat. Commun. 2019, 10, 345), we do not observe a clear splitting of the Raman mode at ~176 cm^{-1}. At room temperature, there is no splitting while the splitting below the transition is about 0.2 cm^{-1}. In this Figure, the solid and dashed lines correspond to the parallel and cross polarizations, respectively.

We note that the spectral resolution of our Raman system is approximately ~1–2 cm^{-1}. In Ref. (Nat. Commun. 2019, 10, 345), the reported splitting is relatively small and becomes more pronounced at lower temperatures than those explored in our study (77 K to 300 K). As such, the absence of a clearly resolved splitting in our measurements may be attributed, in part, to the limited spectral resolution and temperature range. Furthermore, the visibility of such subtle splitting can depend sensitively on experimental factors, including sample thickness, residual strain, and stacking faults, all of which can influence symmetry breaking and the corresponding Raman response. Note that while our measurements do not resolve a clear mode splitting comparable to that reported previously, this does not necessarily contradict earlier findings. Rather, it suggests that the effect is subtle and may depend strongly on sample-specific and experimental conditions.

2. Although findings presented in Fig4 are rather trustful, I find it confusing in how Fig4 is presented. In particular, guide-to-an-eye dashed lines in Fig.4a and b suggest that the transition is continuous (expected from the second order nature of it). Normally this indeed would be the case for an antiferromagnetic-to-paramagnetic transition. However, there is an ongoing discussion in the community of whether these transitions in MPS3s (incl. NiPS3), which involve lattice deformations, can be considered purely of second-order (continuous) [Nat Commun 13, 6598 (2022), Nature 620, 988–993 (2023), Phys. Rev. B 107, L220407 (2023)] or have a first-order (discontinuous) component to it [J. Phys.: Condens. Matter 37 055801 (2025), Phys. Rev. 126, 104 (1962), Hyperfine Interact 16, 625–628 (1983)]. Thus, in case authors have any arguments towards whether this transition is continuous or discontinuous, it would be beneficial to make it clear in the manuscript or comment on that more explicitly.

Response: We thank the reviewer for raising this important point. In the manuscript we did not intend to assign the thermodynamic order of the AFM–PM transition in $NiPS_3$. The dashed lines

in Fig. 4 were included only as guides to the eye to track the evolution of the Rayleigh-wave frequency and phase velocity with temperature. Our Brillouin–Mandelstam measurements probe the elastic response of the system through the Rayleigh surface acoustic mode. The observed anomaly near the Néel temperature reflects a magnetoelastic renormalization of the elastic constants associated with the onset of antiferromagnetic order. However, the present data do not allow us to determine whether the magnetic transition itself is strictly second order or contains a weak first-order component. We removed the dashed line to avoid any possible confusions.

3. According to DFT calculations performed by authors, the largest variation in elastic constants is associated with c66 - the in-plane shear component, which also goes in-line with c11, c12 and c22 change. This is probably due to some small in-plane spontaneous magnetostriction in NiPS3 which was studied previously both experimentally and by DFT [Nat Commun 14, 8503 (2023)]. This effect does deform a lattice in-plane but, although measurable, is usually considered rather poorly pronounced, especially in comparison to CoPS3 or FePS3. The most interesting observation here I believe streams from the sensitivity analysis of elastic constants performed by the authors, which shows that the largest sensitivity to the Rayleigh-wave velocity is from coming from c33 and c44. This suggests a sizable shear- and out-of-plane elastic coefficient softening, which indeed may lead to the reduction in phase velocity, as it is connected to rich and pronounced shear-mode dynamics in NiPS3 - a significant mechanism that is known to couple spins to lattice in this material and even to produce a substantial critical slowdown in relaxation dynamics of both mechanical and magnetic parameters [Nat Commun 13, 6598 (2022)]. I kingly suggest authors to discuss further on these arguments and possibly expand the analysis further on whether these phenomena, i.e. spin-shear deformation and elastic softening (and thus reduction of phase velocity), are “two sides of the same coin”.

Response: We thank the reviewer for this insightful comment and for pointing out the connection between our results and the spin–shear dynamics. Indeed, our DFT calculations indicate that the largest relative change of the elastic tensor across the magnetic transition occurs in the in-plane shear component c66. At the same time, the sensitivity analysis of the Rayleigh-wave velocity shows that the acoustic mode measured in the BMS experiment is most strongly influenced by c33 and c44, which correspond primarily to out-of-plane and shear elastic responses. This apparent difference arises because the Rayleigh surface acoustic wave involves a mixed displacement field combining in-plane longitudinal and out-of-plane transverse components, such that its phase velocity depends on a combination of elastic coefficients rather than a single stiffness component. As a result, even moderate changes in shear-related elastic constants can produce a pronounced effect on the Rayleigh-wave velocity.

We agree with the reviewer that the observed elastic softening and the previously reported spin–shear deformation in NiPS3 reflect closely related manifestations of magnetoelastic coupling. In particular, prior studies have identified interlayer shear as a primary structural degree of freedom that couples directly to the magnetic order parameter and exhibits critical behavior near T_N. In this

framework, the magnetically driven shear distortions modify the elastic tensor, which in turn renormalizes the propagation of long-wavelength acoustic modes such as the Rayleigh wave measured in our BMS experiment. Therefore, the previously reported spin–shear deformation and the elastic softening inferred here can be viewed as two complementary manifestations of the same underlying spin–lattice interaction, probed at different length and time scales. We have revised the manuscript accordingly. The revised paragraph now reads as follows:

"The experimentally observed ~6 % drop in phase velocity at T_N therefore implies a significant reduction in the shear- and out-of-plane-related elastic coefficients, arising from magnetoelastic renormalization triggered by the emergence of zigzag antiferromagnetic order. The observation is consistent with the previously reported spin-shear deformation in $NiPS_3$ [Ref. 50]. Recent studies have identified interlayer shear as a primary structural degree of freedom that couples directly to the magnetic order parameter and exhibits critical behavior near T_N. In this framework, the elastic softening inferred from our measurements can be understood as a continuum-scale manifestation of this spin–shear coupling: the same interaction that drives shear distortions and dynamical critical behavior also renormalizes the effective elastic constants governing long-wavelength acoustic modes. Thus, the previously reported spin–shear deformation and the elastic softening observed here represent two complementary aspects of the same underlying spin–lattice interaction. In this picture, the magnetically driven shear distortions modify the elastic tensor, which in turn renormalizes the velocity of long-wavelength acoustic modes such as the Rayleigh wave measured in our BMS experiment."

4. In the second-to-last sentence of the discussion section authors state that "NiPS3 exhibits an exceptionally large spin-phonon coupling constant" which "places NiPS3 among the strongest spin-phonon-coupled materials", surpassing even FePS3, CrBr3, or CGT by an order of magnitude. I believe this statement cannot be correct: lattice constants of NiPS3 exhibit only minor anomalies near TN, while its in-plane thermal expansion coefficient show a more-or-less normal behavior as a function of temperature, somewhat resembling the trend set by the temperature dependence of its specific heat [Nat Commun 11, 2698 (2020)]. In contrast, both CrBr3 and CGT exhibit extraordinally large anomalies in their thermal expansion coefficients, even turning it negative below Tc [npj Quantum Mater. 6, 19 (2021), Phys. Rev. Research 4, L022040 (2020)]. Also, even if we only consider optical evidence, in case of FePS3 almost the whole low-frequency part of the Raman spectrum is nenormalised below TN, which suggests extremely high spin-phonon coupling [Nano Lett. 16, 12, 7433–7438 (2016), Phys. Rev. Lett. 127, 097401 (2021)]. All this suggests that spins are coupled much weaker to lattice (and thus phonons) in NiPS3 than in the named compounds. Authors may kindly consider to either clarify/rephrase or remove this statement.

Response: We thank the reviewer for this insightful comment and for bringing these important references to our attention. We agree that the strength of spin–lattice coupling depends on the physical observable used to probe it. After discussions with coauthors, we agree that our previous

statement comparing $NiPS_3$ to those aforementioned materials in terms of "order-of-magnitude stronger" spin–phonon coupling could be misleading. Our intention was not to establish a universal ranking across different materials and measurement techniques, but rather to emphasize that $NiPS_3$ exhibits a pronounced magnetoelastic response in the long-wavelength acoustic regime, as directly revealed by the significant softening of the Rayleigh-wave velocity observed in this work. To avoid confusion, we have revised the manuscript to remove the comparative statement and instead clarify that our results highlight a distinct manifestation of spin–lattice coupling in the continuum (elastic) limit, which is complementary to previously reported optical-phonon renormalization, magnon–phonon hybridization, and thermal-expansion anomalies in other van der Waals magnets.

We truly thank the reviewer for pointing out these valuable works, which have now been incorporated and cited in the revised manuscript to provide a more comprehensive context for spin–lattice coupling across different material systems and experimental probes. We revised the manuscript as follows:

"The experimentally observed ∼6 % drop in phase velocity at T_N therefore implies a significant reduction in the shear- and out-of-plane-related elastic coefficients arising from magnetoelastic renormalization triggered by the emergence of zigzag antiferromagnetic order. The observation is consistent with the previously reported spin-shear deformation in $NiPS_3$ [Ref. [3]]. In this picture, the magnetically driven shear distortions modify the elastic tensor, which in turn renormalizes the velocity of long-wavelength acoustic modes such as the Rayleigh wave measured in our BMS experiment. These results show that the spin-driven reconfiguration of the elastic tensor in $NiPS_3$ is sufficiently large to measurably alter the propagation of surface acoustic phonons, revealing pronounced spin–lattice interaction at long wavelengths. Materials such as $FePS_3$, $CrBr_3$, and $Cr_2Ge_2Te_6$ have been shown to exhibit strong spin–lattice coupling manifested through optical phonon renormalization, magnon–phonon hybridization, and pronounced thermal-expansion anomalies[4–10]. In contrast, the Rayleigh-wave anomaly observed here highlights a complementary manifestation of spin–lattice coupling in $NiPS_3$ in the long-wavelength acoustic (continuum) regime. The Rayleigh-wave anomaly observed here provides a direct long-wavelength spectroscopic manifestation of spin–phonon coupling, establishing $NiPS_3$ as a promising platform for studying magnetoelastic interactions and phonon-mediated functionalities in low-dimensional antiferromagnets."

We thank Reviewer 2 for the constructive comments, valuable references, and insightful discussion. We sincerely appreciate these suggestions and believe that they have significantly strengthened the manuscript.

At the end, we sincerely thank both Reviewer 1 and Reviewer 2 for their constructive feedback and insightful comments. We hope that the revised manuscript satisfactorily addresses all concerns.

REFERENCES

1. Kargar, F. & Balandin, A. A. Advances in Brillouin–Mandelstam light-scattering spectroscopy. *Nat. Photonics* **15**, 720–731 (2021).

2. Ran, J. *et al.* $NiPS_3$ ultrathin nanosheets as versatile platform advancing highly active photocatalytic H_2 production. *Nat. Commun. 2022 131* **13**, 4600- (2022).

3. Zhou, F. *et al.* Dynamical criticality of spin-shear coupling in van der Waals antiferromagnets. *Nat. Commun. 2022 131* **13**, 6598- (2022).

4. Liu, Y., Liu, Y., Zhao, J., & Jiang, X. Spin-Phonon Coupling on 2D $NiPS_3$ Nanosheets: Implications for Antiferromagnetic Spintronic and Spin Caloritronic Devices. *ACS Appl. Nano Mater* ***8**,* 2291-2298 (2025).

5. Ghosh, A. *et al.* Spin-phonon coupling and magnon scattering in few-layer antiferromagnetic $FePS_3$. *Phys. Rev. B* **103**, 064431 (2021).

6. Kozlenko, D. P. *et al.* Spin-induced negative thermal expansion and spin–phonon coupling in van der Waals material $CrBr_3$. *npj Quantum Mater.* **6**, 1–5 (2021).

7. Zhang, B. H., Hou, Y. S., Wang, Z. & Wu, R. Q. First-principles studies of spin-phonon coupling in monolayer $Cr_2Ge_2Te_6$. *Phys. Rev. B* **100**, 224427 (2019).

8. Spachmann, S. *et al.* Strong effects of uniaxial pressure and short-range correlations in $Cr_2Ge_2Te_6$. *Phys. Rev. Res.* **4**, L022040 (2022).

9. Lee, J. U. *et al.* Ising-Type Magnetic Ordering in Atomically Thin $FePS_3$. *Nano Lett.* **16**, 7433–7438 (2016).

10. Liu, S. *et al.* Direct Observation of Magnon-Phonon Strong Coupling in Two-Dimensional Antiferromagnet at High Magnetic Fields. *Phys. Rev. Lett.* **127**, 097401 (2021).

REVIEWER COMMENTS

Reviewer #1 (Remarks to the Author):

The authors have addressed most of my concerns in a substantive manner. In particular, they added measurements on an additional NiPS3 flake with a different thickness, provided denser temperature-dependent BMS data near the Néel temperature, and clarified the scattering geometry and the meaning of the incidence angle θ. These additions significantly improve the systematicity and reproducibility of the experimental evidence supporting Rayleigh-wave softening across the magnetic transition.

The authors have also supplemented the crystallographic and directional analyses using TEM, angle-resolved Raman, and BMS measurements along different edge-related directions. The additional TEM measurements reassign the sample edges from the a-axis to the b-axis. Although the revised results still do not identify which of the three edge-related directions corresponds to the b-axis, the authors have removed the previous statement implying direct identification of the crystallographic axis. Given that this remaining ambiguity appears to have limited impact on the main BMS results, I consider this issue acceptable. The added analyses and more cautious wording improve the rigor of the paper.

Response: We sincerely thank the reviewer for the positive assessment of our revised manuscript and for recognizing that the additional measurements and analyses "significantly improve the systematicity and reproducibility" of our results and "improve the rigor of the paper." We also appreciate the reviewer's thoughtful acknowledgment that the remaining crystallographic ambiguity has "limited impact on the main BMS results" and is therefore an "acceptable" issue.

One remaining point concerns the comparison between the two temperature-dependent datasets. The additional measurements on the 260 nm flake are appreciated and clearly expand the dataset. However, the detailed temperature evolution of the anomaly does not appear entirely identical in the two samples. In the 220 nm sample, the frequency initially follows the expected lattice-stiffening trend upon cooling from room temperature to approximately 200 K, followed by a distinct drop near TN and remaining nearly constant at lower temperatures. In contrast, the 260 nm sample exhibits a much sharper and more localized anomaly around TN, rather than a gradual evolution over a broader temperature range. While the manuscript treats the two datasets as demonstrating the same qualitative behavior, the apparent differences in the evolution and character of the anomaly deserve some discussion. Since both the sample thickness and phonon wavevector differ between the two measurements, it remains unclear whether these distinctions arise from thickness effects, wavevector dependence, sample variation, or different manifestations of the underlying magnetoelastic response.

Response: We agree that the two datasets appear somewhat different at first glance; however, we believe that this impression is primarily a consequence of the different density of temperature

sampling and the guide-to-the-eye lines used to connect the experimental points, rather than an intrinsic difference in the magnetoelastic response. Indeed, both datasets exhibit the same overall behavior: (i) phonon hardening upon cooling in the paramagnetic phase, (ii) pronounced magnetoelastic softening in the vicinity of the magnetic transition, and (iii) recovery to a nearly temperature-independent behavior deep in the antiferromagnetic phase (0.05-0.07 GHz changes). Quantitatively, the magnitude of the phonon hardening above the transition is very similar in the two samples (approximately 0.13 GHz for the 220 nm flake and approximately 0.10 GHz for the 260 nm flake), while the softening associated with the transition is likewise comparable (approximately 0.23 GHz and 0.30 GHz, respectively). The apparent "gradual" anomaly in the 220 nm sample versus the seemingly "sharper" anomaly in the 260 nm sample is therefore largely a visual effect arising from the different temperature spacing of the measured data points. The 260 nm sample was measured with a much denser temperature grid around the transition, whereas the 220 nm dataset contains fewer temperature points, causing the guide-to-the-eye line to suggest a broader evolution than is experimentally resolved. Note that the absolute Rayleigh-wave frequencies differ between the two samples, with the thinner (220 nm) flake exhibiting higher frequencies than the 260 nm flake. This difference is expected because the measured Rayleigh-like guided surface acoustic mode depends on the flake thickness, and therefore the absolute frequency is thickness-dependent. Consequently, while the baseline frequencies differ, the relative magnetoelastic softening across the magnetic transition is remarkably consistent between the two independent samples, supporting the same underlying magnetoelastic mechanism.

Given the extremely time-intensive nature of these Brillouin measurements, we believe that the agreement between the two independent datasets is remarkably good and supports the same underlying magnetoelastic mechanism. To clarify this point for the reader, we have added a discussion to the revised manuscript explaining that the apparent differences in the shape of the anomaly primarily reflect the different temperature sampling density rather than a fundamental difference in physical behavior. The corresponding paragraph has been revised as follows (new text shown in red for clarity):

"The Rayleigh surface acoustic wave is governed by the elastic constants of the crystal, making its frequency highly sensitive to changes in lattice stiffness associated with magnetic ordering[45,46]. Therefore, the temperature dependence of the Rayleigh mode provides a direct probe of magnetoelastic coupling across the AFM–PM transition. To track the Rayleigh wave evolution with temperature, BMS measurements were conducted from 300 K to 80 K while maintaining a constant in-plane wavevector, q, for each sample. Due to the highly time-intensive nature of temperature-dependent BMS measurements, we first investigated the 220 nm-thick $NiPS_3$ flake using relatively large temperature intervals. The obtained spectra, accumulated at a constant incident angle of $\theta = 35°$, corresponding to the wavevector $q = 0.0135\ \mathrm{nm}^{-1}$, are shown in Fig. 5a. These measurements revealed a pronounced softening of the Rayleigh mode in the vicinity of the Néel temperature at ~150 K. To verify the reproducibility of this behavior and resolve the

transition with higher precision, we repeated the measurements on a 260 nm-thick flake using a much denser temperature grid around the magnetic transition. The measurements were conducted at $\theta = 40°$ corresponding to $q = 0.0152\ \mathrm{nm}^{-1}$. The obtained spectra are presented in Fig. 5 (c). Each spectrum in Figs. 5 (a) and (c) was fit using a single Lorentzian function. As seen, the spectral position of the Rayleigh peak increases gradually upon cooling from RT to ~200 K, followed by a distinct drop at around T_N, and then remains nearly constant at lower temperatures. The initial increase reflects the expected reduction of anharmonic lattice vibrations upon cooling, which leads to lattice stiffening and thus a higher acoustic phase velocity. The drop in frequency at ~150 K is associated with the onset of AFM order, where spin rearrangement modifies the elastic constants through magnetoelastic coupling, resulting in a measurable softening. Note that although the two datasets may appear somewhat different at first glance, this primarily reflects the different temperature intervals used during data acquisition rather than an intrinsic difference in the underlying behavior. The more sparsely sampled 220 nm dataset gives the impression of a broader anomaly, whereas the finer temperature resolution of the 260 nm measurements resolves the same feature as a sharper transition. Nevertheless, closer inspection shows that the two datasets exhibit remarkably similar quantitative behavior**,** with phonon hardening above T_N of approximately 0.13 GHz for the 220 nm and 0.10 GHz for the 260 nm flake and magnetoelastic softening across the transition of approximately 0.23 GHz and 0.30 GHz for the 220 nm and 260 nm films, respectively. Both samples show nearly temperature-independent frequencies deep in the antiferromagnetic phase with only ~0.05–0.07 GHz variation. The similar softening of the Rayleigh mode is quantitatively reflected in the phase velocity of the Rayleigh waves, plotted in Figs. 5(b) and (d), which decreases from ~1920 ms^{-1} in the PM state to ~1815 ms^{-1} in the AFM state, corresponding to a ~6 % reduction across the transition for the 220 nm film. The phase velocity for 260 nm film reduces from 1610 ms^{-1} to 1500 ms^{-1} which corresponds to ~ 7.3 % decrease in phase velocity. Once the magnetic order is established, the elastic constants stabilize, resulting in a nearly temperature-independent frequency at low temperatures. These results further support the weak anisotropy nature of the basal plane, as the phonon wavevector – probed perpendicular to random crystal edges in the two films – yields phonon velocity softening that varies by only ~1%."

Reviewer #2 (Remarks to the Author):

I believe the authors have significantly improved the manuscript following this round of revisions. Their responses to my comments are clear, thorough, and convincing, and they have adequately addressed all of my concerns. I have no further objections and recommend this work for publication.

Response: We thank the reviewer for the thorough assessment of our revised manuscript and for the valuable comments and suggestions provided during the review process. We appreciate the positive evaluation of the revised work and the recommendation for publication.
